# Supplementary figures and images for: Questionable research practices in competitive grant funding: A survey
Source: PLoS One. 2023 Nov 2;18(11):e0293310. doi: 10.1371/journal.pone.0293310 (PMC10621923; doi:10.1371/journal.pone.0293310)

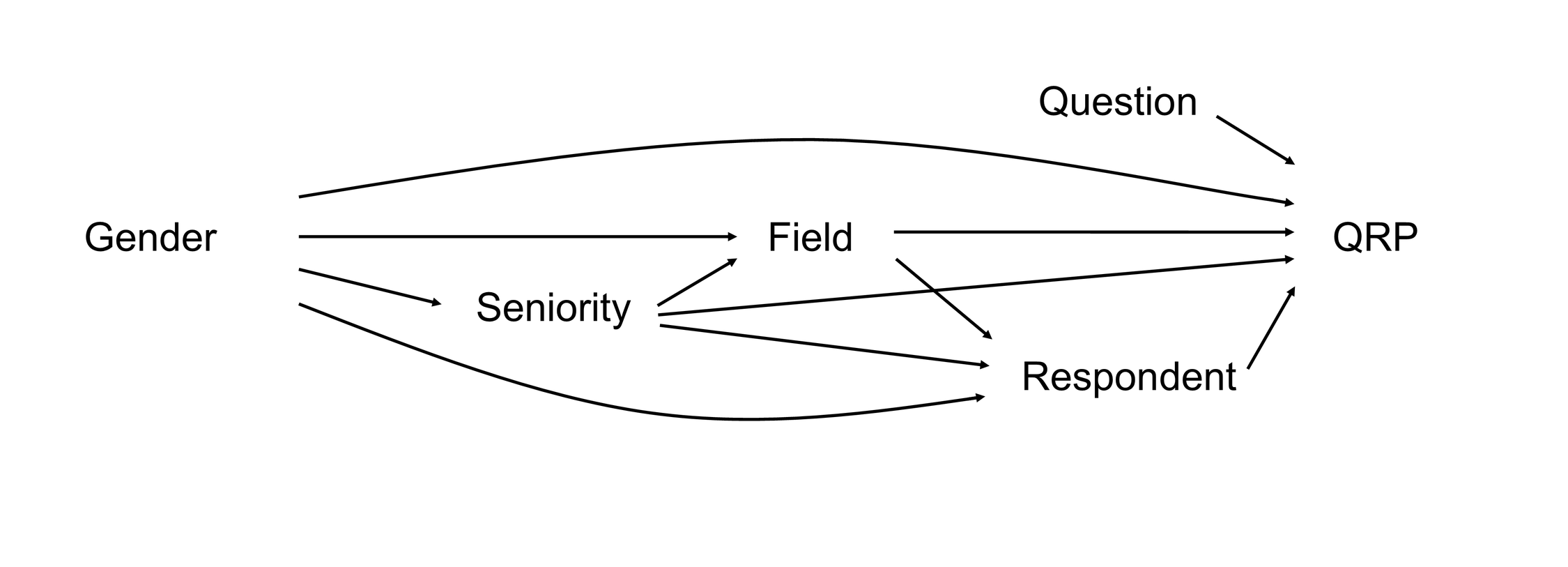

Supplement: S1 Fig — Directed acyclic graph that expresses our causal assumptions for the gender hypothesis tests. For the hypothesis tests with aggregated variables, the ‘Respondent’ and ‘Question’ variables and the edges connected to them should be removed. (TIF) [file pone.0293310.s001.tif]

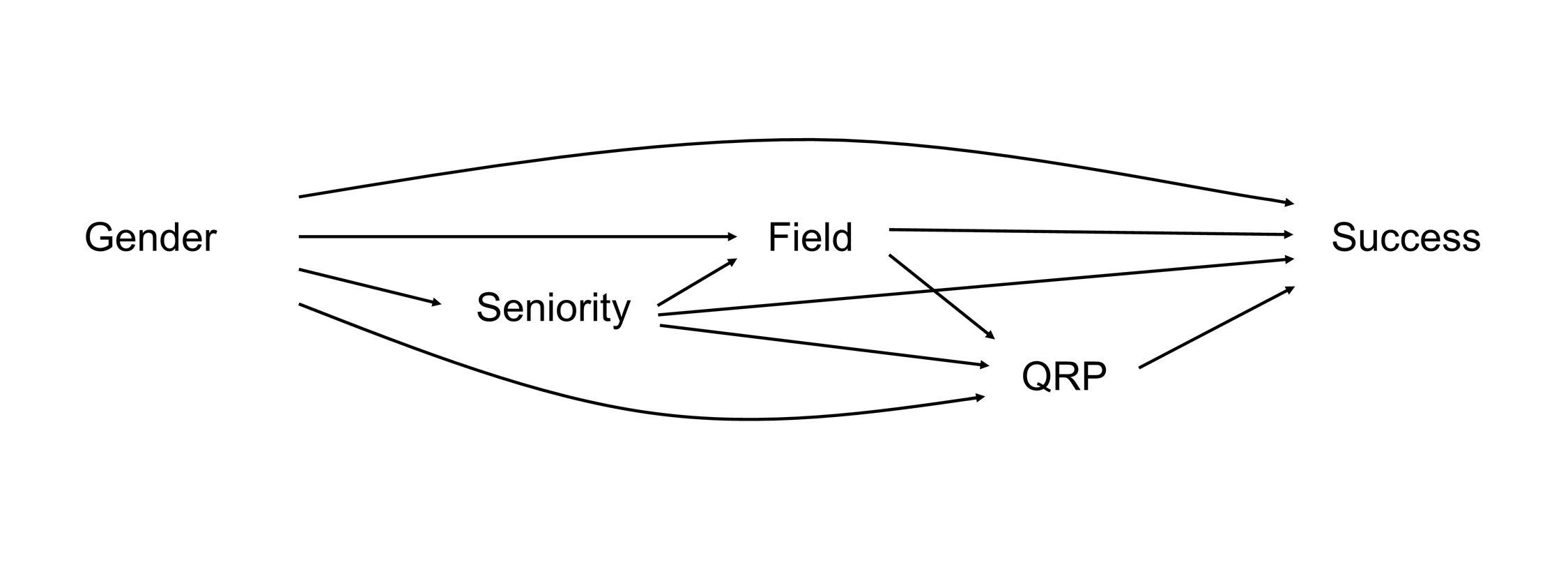

Supplement: S2 Fig — Directed acyclic graph that expresses our causal assumptions for the success hypothesis tests. (TIF) [file pone.0293310.s002.tif]

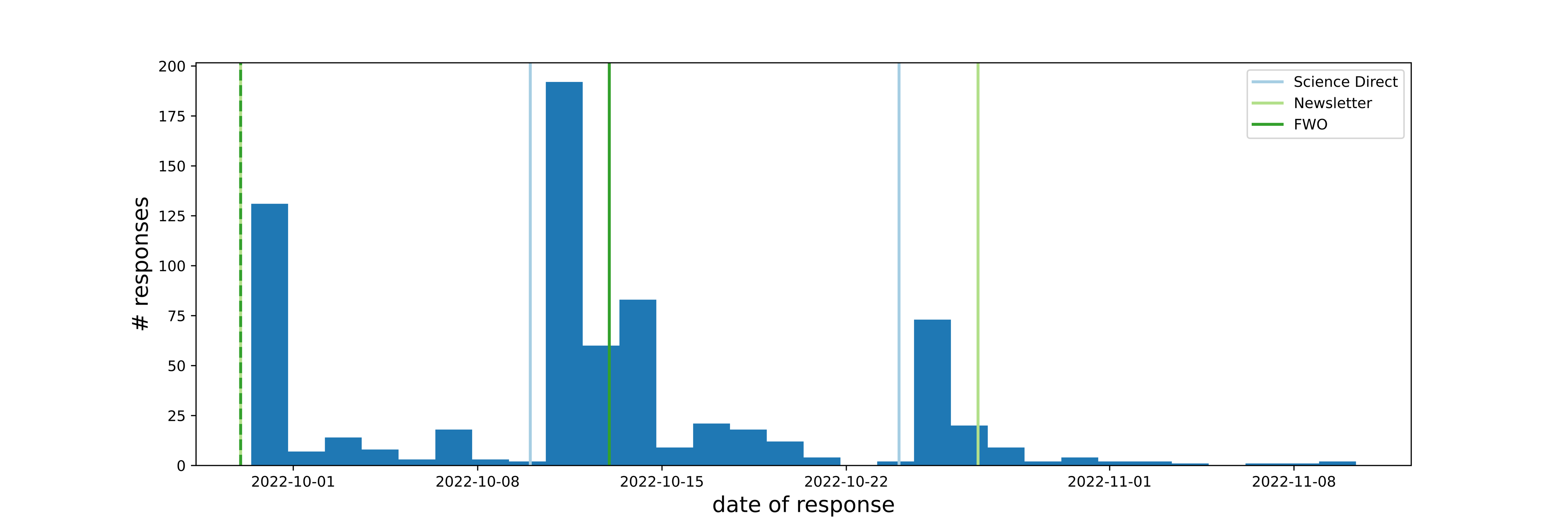

Supplement: S3 Fig — (TIF) [file pone.0293310.s003.tif]

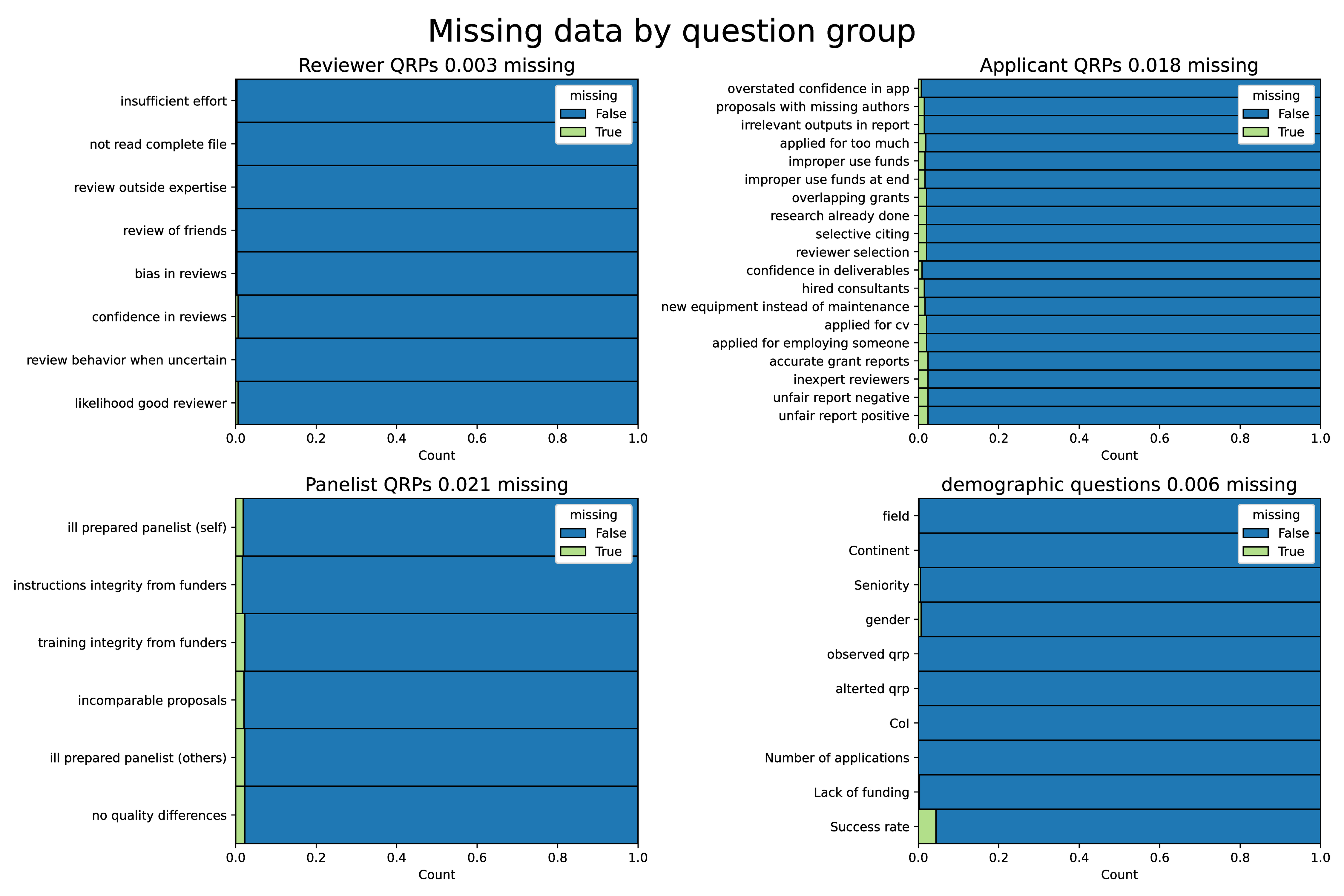

Supplement: S4 Fig — Proportion of respondents that were asked the question but did not answer it. Hence, this does not include questions for roles that the respondents did not indicated they had played. (TIF) [file pone.0293310.s004.tif]

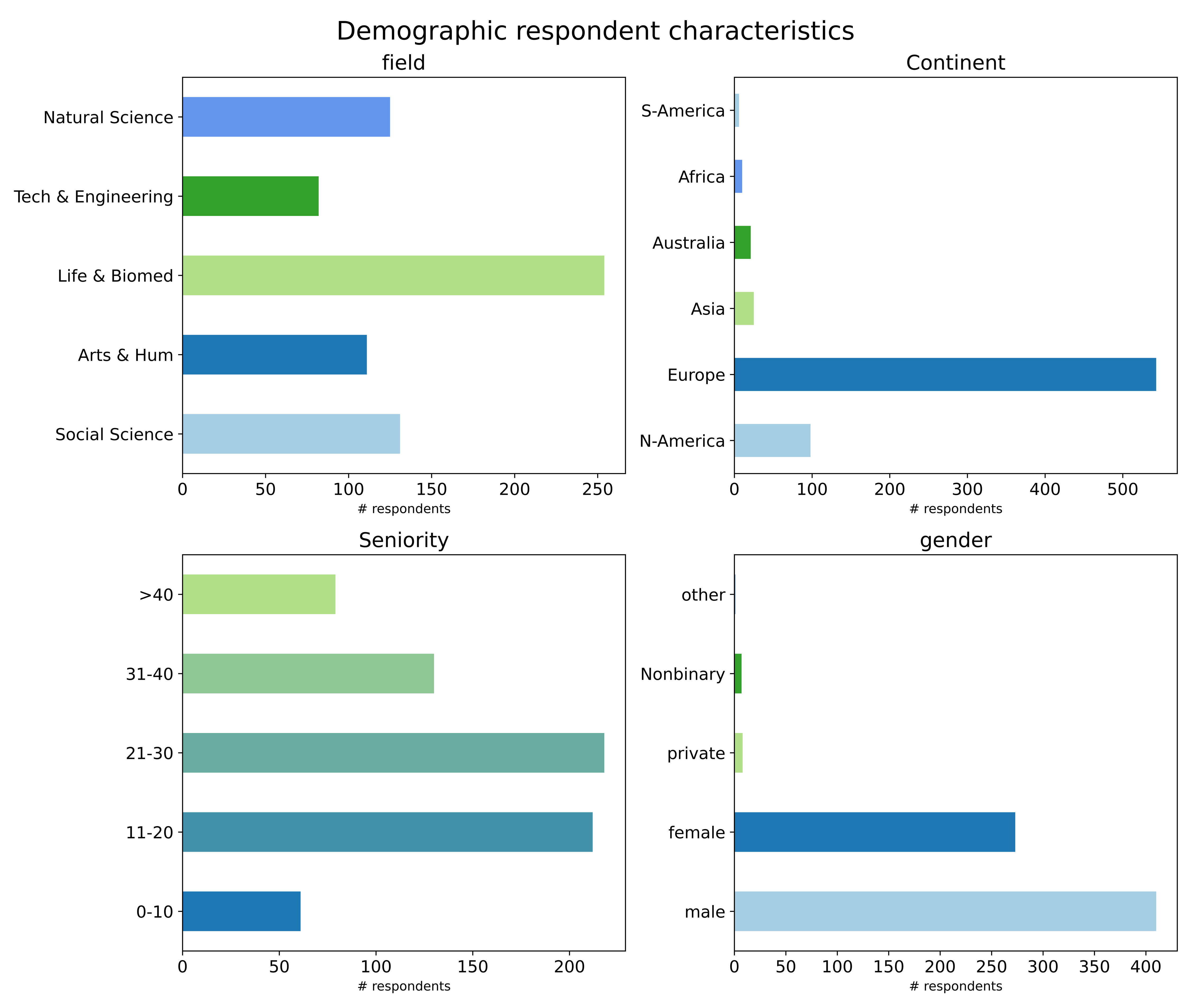

Supplement: S5 Fig — (TIF) [file pone.0293310.s005.tif]

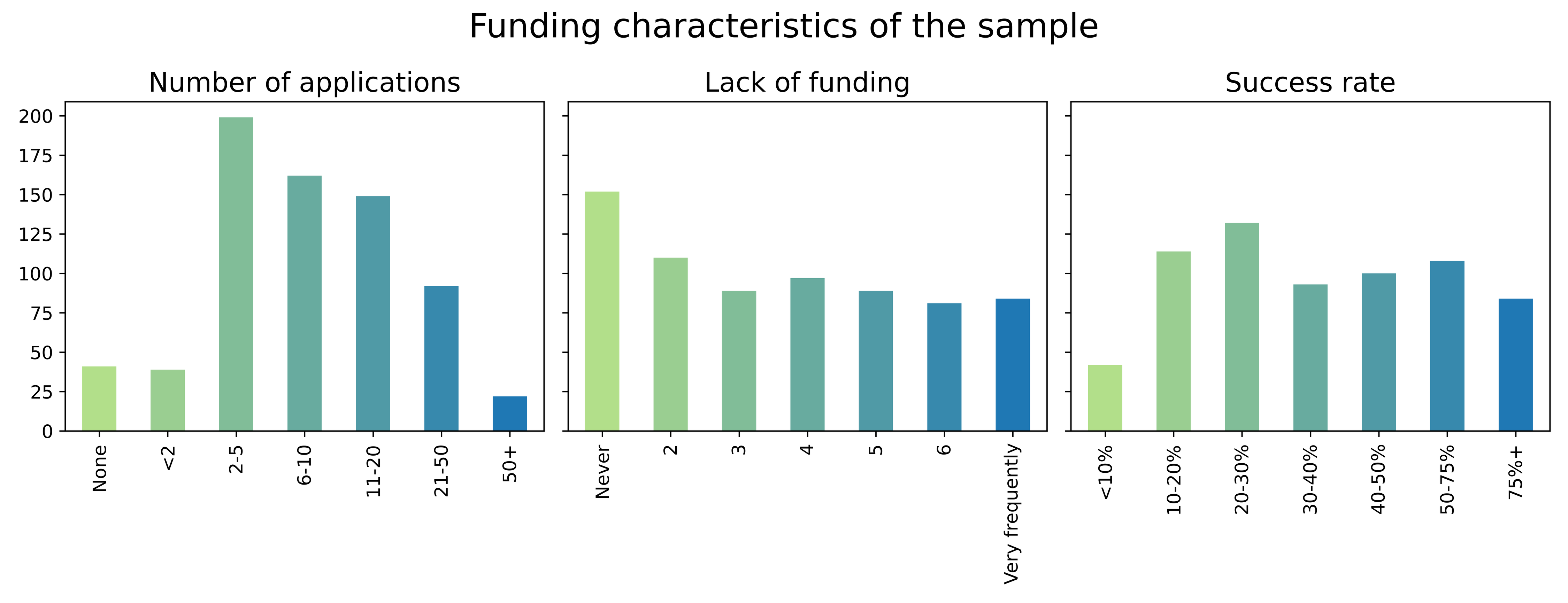

Supplement: S6 Fig — (TIF) [file pone.0293310.s006.tif]

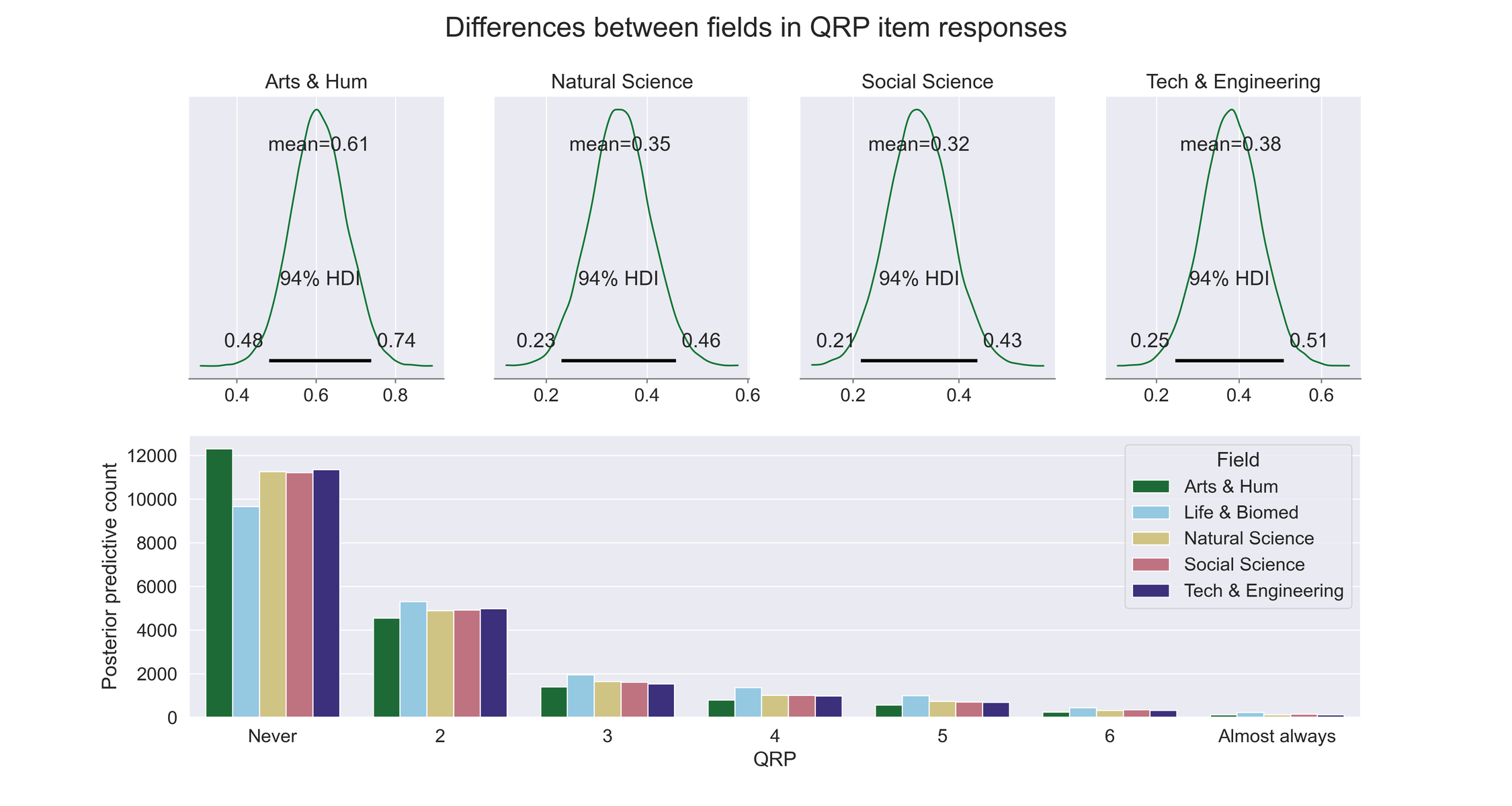

Supplement: S7 Fig — The top row shows the posterior distribution of the differences in coefficients between ‘Life and Biomedical Sciences’ and the other fields. The bottom row shows the distribution of QRP item response scores for 20000 posterior predictive samples drawn from the study population but setting their field in turn to each of the fields. (TIF) [file pone.0293310.s007.tif]

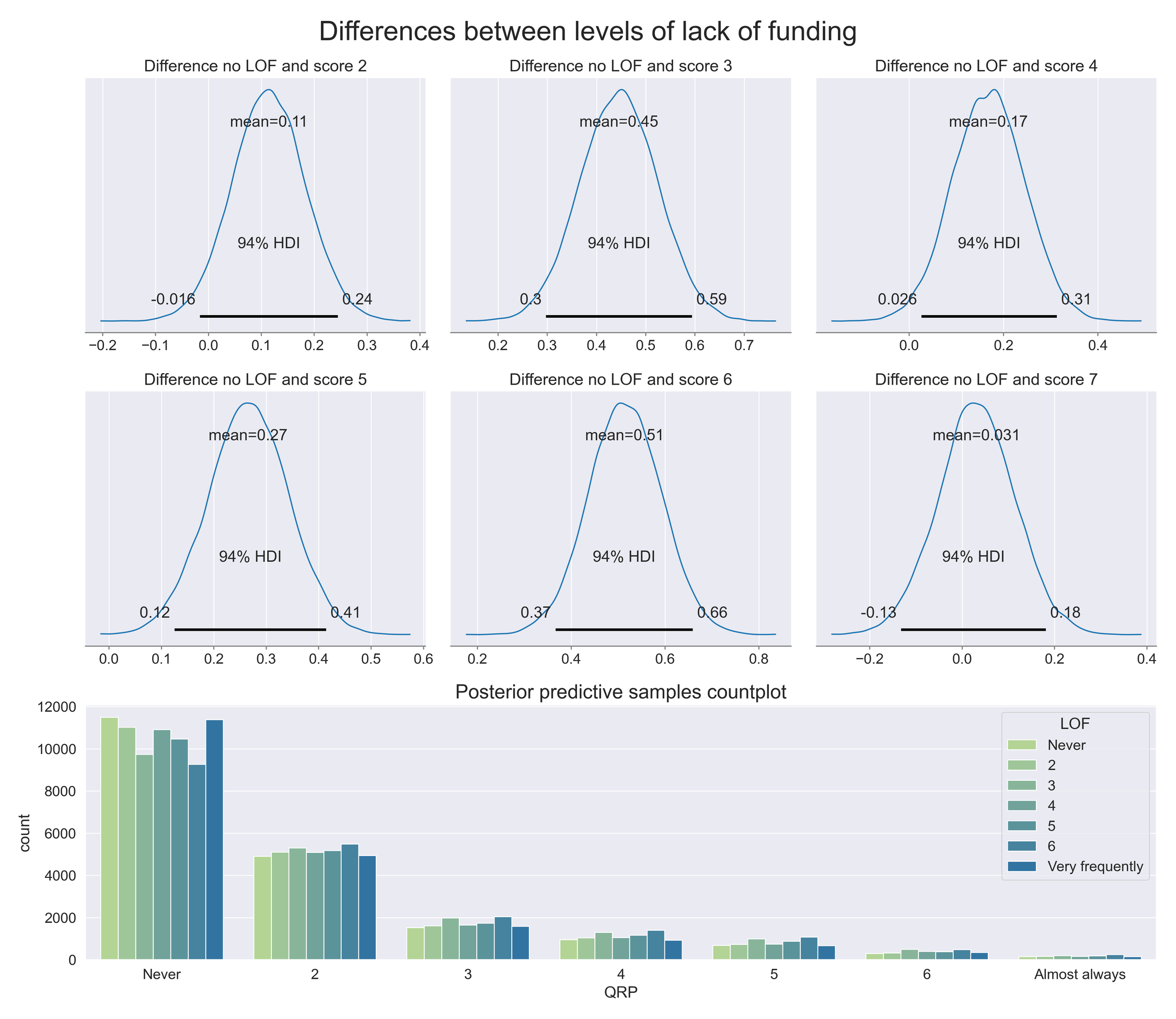

Supplement: S8 Fig — The two top row shows the posterior distribution of the differences in coefficients between ‘No lack of funding’ and the other levels. The bottom row shows the distribution of QRP item response scores for 20000 posterior predictive samples drawn from the study population but setting their field in turn to each of the levels of lack of funding. (TIF) [file pone.0293310.s008.tif]

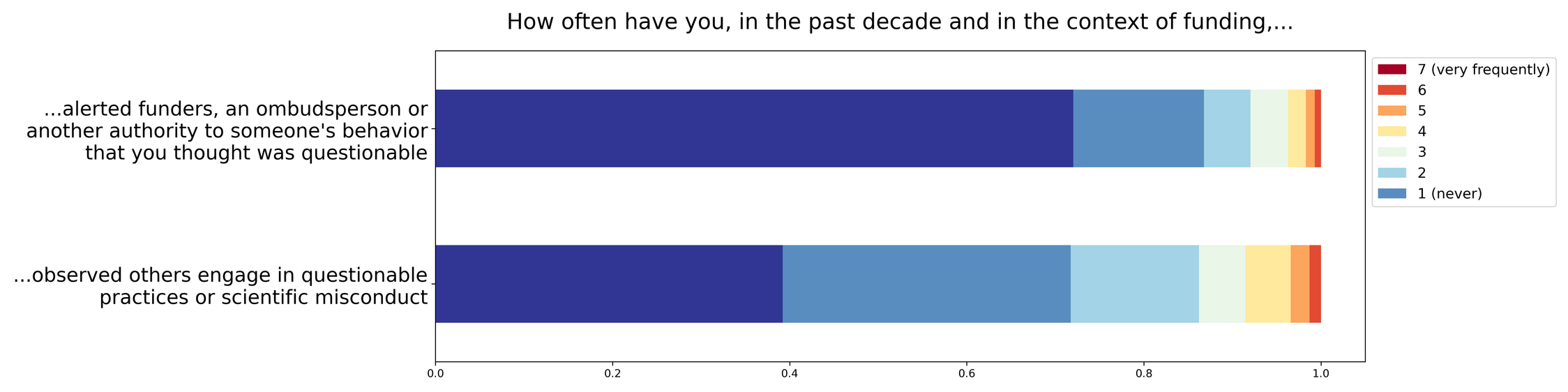

Supplement: S9 Fig — (TIF) [file pone.0293310.s009.tif]

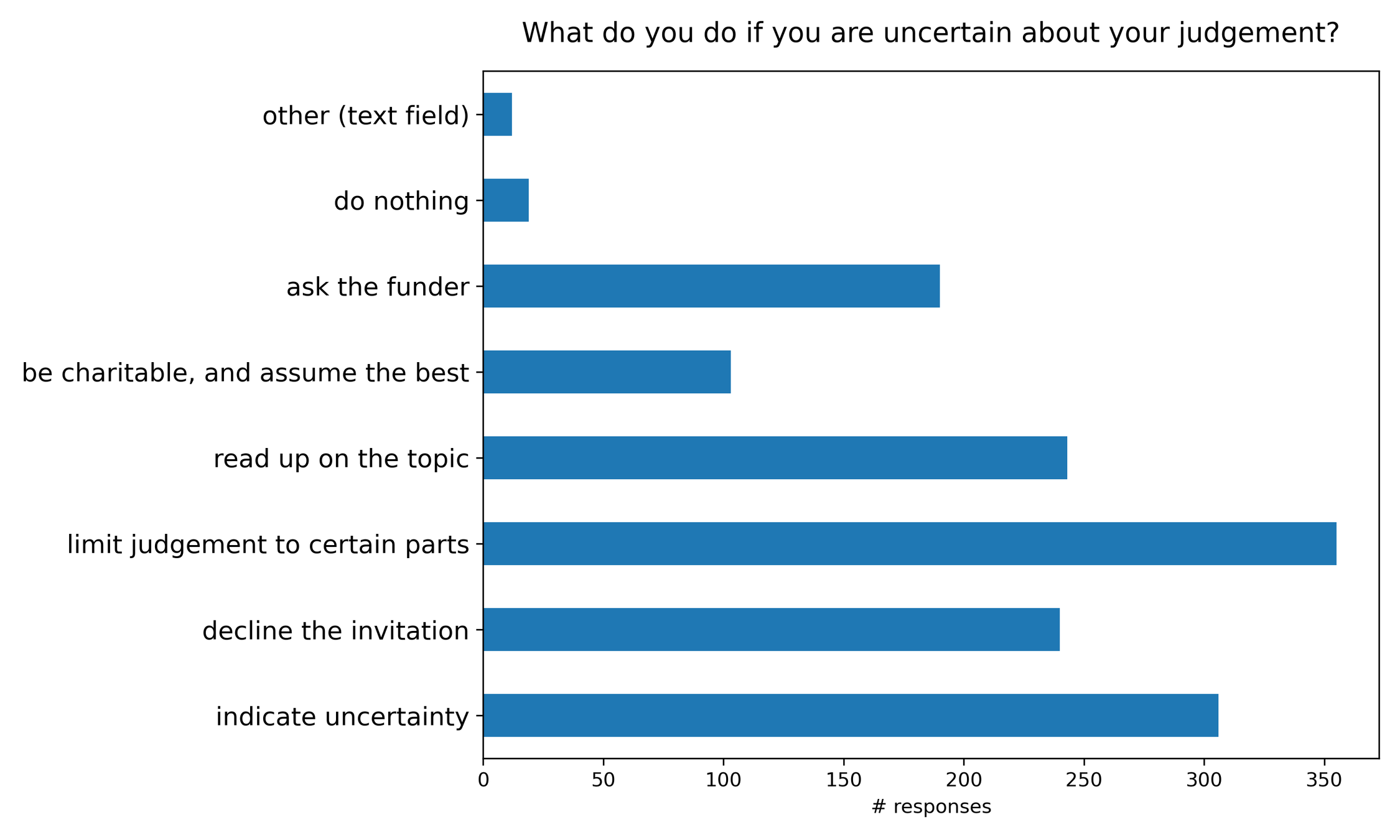

Supplement: S10 Fig — (TIF) [file pone.0293310.s010.tif]
