# Supplementary material for: Questionable research practices in competitive grant funding: A survey
Source: PLoS One. 2023 Nov 2;18(11):e0293310. doi: 10.1371/journal.pone.0293310 (PMC10621923; doi:10.1371/journal.pone.0293310)
Supplement: S1 Table — (DOCX) [file pone.0293310.s011.docx]

**S1 Table. Seniority and field by gender of the respondents.**

|  | **Field** | | | | | **Seniority** | | | | | **Total** |
| --- | --- | --- | --- | --- | --- | --- | --- | --- | --- | --- | --- |
|  | **Arts & Humanities** (N = 130) | **Life & Biomededical sciences** (N = 109) | **Natural Sciences** (N = 124) | **Social Sciences** (N = 82) | **Technology & Engineering** (N = 253) | **0-10** (N = 58) | **11-20** (N = 211) | **21-30** (N = 217) | **31-40** (N = 130) | **>40** (N = 79) |  |
| **Female** | 7.88% | 6.59% | 6.59% | 4.30% | 13.61% | 3.60% | 12.81% | 11.65% | 6.33% | 4.75% | 39.14% (N = 272) |
| **Male** | 9.89% | 8.60% | 10.89% | 7.16% | 22.21% | 4.32% | 16.55% | 19.14% | 12.23% | 6.33% | 58.56% (N = 410) |
| **Other** | 0.86% | 0.43% | 0.29% | 0.29% | 0.43% | 0.43% | 1.01% | 0.43% | 0.14% | 0.29% | 2.30% (N = 16) |
| **Total** | 18.62% | 15.62% | 17.77% | 11.75% | 36.25% | 8.35% | 30.36% | 31.22% | 18.71% | 11.37% | 100% (N = 695^a^) |

Note: The number of participants included in this table (695) is lower than the total responses retained (704) due to a number of respondent characteristic questions that were left open and removed only for this table.
