# Supplementary material for: Questionable research practices in competitive grant funding: A survey
Source: PLoS One. 2023 Nov 2;18(11):e0293310. doi: 10.1371/journal.pone.0293310 (PMC10621923; doi:10.1371/journal.pone.0293310)
Supplement: S2 Table — (DOCX) [file pone.0293310.s012.docx]

**Table S2. Expected and observed proportion of responses by field.**

|  | **Applications at FWO** | **Expected FWO** | **Expected Science Direct** | **Expected total** | **Observed** | **Expected proportion** |
| --- | --- | --- | --- | --- | --- | --- |
| **Life & Biomedical** | 43.85% | 312 | 395 | 707 | 36.10% | 40.54% |
| **Arts & Humanities** | 8.64% | 120 | 144 | 264 | 15.80% | 15.14% |
| **Social Sciences** | 19.67% | 96 | 176 | 272 | 18.60% | 15.60% |
| **Natural Sciences** | 12.05% | 120 | 133 | 253 | 17.80% | 14.51% |
| **Technology & Engineering** | 12.05% | 96 | 152 | 248 | 11.70% | 14.22% |
| **Sum** | 96.26% | 744 | 1000 | 1744 | 100% | 100.00% |

Note: ‘Applications at FWO’ is gathered from the annual reports of FWO 2017 – 2021 (<https://www.vlaanderen.be/publicaties/jaarverslag-fonds-wetenschappelijk-onderzoek-fwo>). Expected SD (Science Direct) is the breakdown by field of the reviewers that Science Direct sent the survey to. ‘Observed’ are the proportions of the 5 fields in the observed sample. The file with all data relevant to the breakdown of the sample by field is uploaded to the OSF page of the project (<https://osf.io/jk6wd/files/osfstorage>) under the name ERF_sample_data_FWO.
