# Supplementary material for: Questionable research practices in competitive grant funding: A survey
Source: PLoS One. 2023 Nov 2;18(11):e0293310. doi: 10.1371/journal.pone.0293310 (PMC10621923; doi:10.1371/journal.pone.0293310)
Supplement: S1 File — The questionnaire exported from Qualtrics into a.docx file. (DOCX) [file pone.0293310.s013.docx]

Ethics of Funding – FWO/NWO

Survey Flow

Standard: Landing page (1 Question)

Block: Respondent Characteristics (10 Questions)

Branch: New Branch

If

If In the past decade, in which of the following capacities have you participated in the research fu... None of the above Is Selected

EndSurvey:

Branch: New Branch

If

If In the past decade, in which of the following capacities have you participated in the research fu... <strong>(Co-)author</strong> of, <strong>or contributor</strong> to, a research-project proposal submitted as part of an application for research funding Is Selected

And In the past decade, in which of the following capacities have you participated in the research fu... <strong>Member of a panel</strong>&nbsp;evaluating research-project proposals submitted as part of an application for research funding Is Not Selected

And In the past decade, in which of the following capacities have you participated in the research fu... <strong>Reviewer of project proposals</strong> that were submitted as part of an application for research funding Is Not Selected

Block: Applicants (26 Questions)

EndSurvey:

Branch: New Branch

If

If In the past decade, in which of the following capacities have you participated in the research fu... <strong>Reviewer of project proposals</strong> that were submitted as part of an application for research funding Is Selected

And In the past decade, in which of the following capacities have you participated in the research fu... <strong>Member of a panel</strong>&nbsp;evaluating research-project proposals submitted as part of an application for research funding Is Not Selected

And In the past decade, in which of the following capacities have you participated in the research fu... <strong>(Co-)author</strong> of, <strong>or contributor</strong> to, a research-project proposal submitted as part of an application for research funding Is Not Selected

Block: Reviewers (11 Questions)

EndSurvey:

Branch: New Branch

If

If In the past decade, in which of the following capacities have you participated in the research fu... <strong>Member of a panel</strong>&nbsp;evaluating research-project proposals submitted as part of an application for research funding Is Selected

And In the past decade, in which of the following capacities have you participated in the research fu... <strong>Reviewer of project proposals</strong> that were submitted as part of an application for research funding Is Not Selected

And In the past decade, in which of the following capacities have you participated in the research fu... <strong>(Co-)author</strong> of, <strong>or contributor</strong> to, a research-project proposal submitted as part of an application for research funding Is Not Selected

Block: Panellists (8 Questions)

Standard: CoI (2 Questions)

EndSurvey:

Branch: New Branch

If

If In the past decade, in which of the following capacities have you participated in the research fu... <strong>Member of a panel</strong>&nbsp;evaluating research-project proposals submitted as part of an application for research funding Is Not Selected

And In the past decade, in which of the following capacities have you participated in the research fu... <strong>Reviewer of project proposals</strong> that were submitted as part of an application for research funding Is Selected

And In the past decade, in which of the following capacities have you participated in the research fu... <strong>(Co-)author</strong> of, <strong>or contributor</strong> to, a research-project proposal submitted as part of an application for research funding Is Selected

BlockRandomizer: 2 - Evenly Present Elements

Block: Reviewers (11 Questions)

Block: Applicants (26 Questions)

EndSurvey:

Branch: New Branch

If

If In the past decade, in which of the following capacities have you participated in the research fu... <strong>Member of a panel</strong>&nbsp;evaluating research-project proposals submitted as part of an application for research funding Is Selected

And In the past decade, in which of the following capacities have you participated in the research fu... <strong>(Co-)author</strong> of, <strong>or contributor</strong> to, a research-project proposal submitted as part of an application for research funding Is Selected

And In the past decade, in which of the following capacities have you participated in the research fu... <strong>Reviewer of project proposals</strong> that were submitted as part of an application for research funding Is Not Selected

BlockRandomizer: 2 - Evenly Present Elements

Block: Panellists (8 Questions)

Block: Applicants (26 Questions)

EndSurvey:

Branch: New Branch

If

If In the past decade, in which of the following capacities have you participated in the research fu... <strong>Member of a panel</strong>&nbsp;evaluating research-project proposals submitted as part of an application for research funding Is Selected

And In the past decade, in which of the following capacities have you participated in the research fu... <strong>Reviewer of project proposals</strong> that were submitted as part of an application for research funding Is Selected

And In the past decade, in which of the following capacities have you participated in the research fu... <strong>(Co-)author</strong> of, <strong>or contributor</strong> to, a research-project proposal submitted as part of an application for research funding Is Not Selected

BlockRandomizer: 2 - Evenly Present Elements

Block: Panellists (8 Questions)

Block: Reviewers (11 Questions)

EndSurvey:

Branch: New Branch

If

If In the past decade, in which of the following capacities have you participated in the research fu... <strong>Member of a panel</strong>&nbsp;evaluating research-project proposals submitted as part of an application for research funding Is Selected

And In the past decade, in which of the following capacities have you participated in the research fu... <strong>Reviewer of project proposals</strong> that were submitted as part of an application for research funding Is Selected

And In the past decade, in which of the following capacities have you participated in the research fu... <strong>Reviewer of project proposals</strong> that were submitted as part of an application for research funding Is Selected

BlockRandomizer: 3 - Evenly Present Elements

Block: Panellists (8 Questions)

Block: Reviewers (11 Questions)

Block: Applicants (26 Questions)

Block: CoI (2 Questions)

EndSurvey:

| Page Break |  |
| --- | --- |

Start of Block: Landing page

Intro Welcome to this survey concerning the ethical aspects of competitive research-funding. Its purpose is to explore the practices and processes involved in applying for competitive grants as well as in the review of such applications. Depending on the capacity in which you may have participated in such processes – viz. applicant, reviewer and/or panelist – you will be asked to answer specific questions. These would also include some demographical questions. The survey will take 10-15 minutes to complete.

 **Confidentiality** ***All information obtained from your session would be anonymous and confidential***. The researchers cannot identify you. The data collected for this study are stored for at least 10 years. Collected data may be made available to researchers via accessible data-repositories and may possibly be used for novel academic purposes.
   **Voluntary Nature of Participation** Your decision to participate in this study is entirely voluntary. Should you choose not to participate, you are free to opt out at any time without any consequences. In order to terminate your participation at any point, you need only to close the browser window.
   **Risks/Discomforts** There is a minor risk that, due to the nature of the questions you will be asked, you may become uncomfortable. Should this occur at any point during the survey, you may elect to withdraw your consent to participate without any consequences for you. Simply close the browser window. You are also free to contact the lead investigator with any questions or concerns regarding your discomfort, using the email address listed at the bottom of this consent form.
   **Benefits** As a small incentive and token of our appreciation of your time, we will donate €1 to the [Malaria Consortium](https://www.givewell.org/charities/malaria-consortium) for each full survey response we get. These donations will be used to buy medicine to prevent malaria, which [givewell.org](https://givewell.org/) currently lists as one of the most effective charities. For more information on why we chose this charity, visit [givewell](https://www.givewell.org/) or the [effective altruism webpage](https://www.effectivealtruism.org/).
   **Contact Information** Should you have any queries or concerns regarding the study, please feel free to email Dr. Stijn Conix ([stijn.conix@kuleuven.be](mailto:stijn.conix@kuleuven.be)) in confidence. The study has been approved by the [Social and Societal Ethics Committee of KU Leuven](https://www.kuleuven.be/english/research/ethics/committees/smec) (file G-2021-4529-R2(MIN)). If you are concerned or wish to complain about an ethical aspect of the study, you may contact the aforementioned committee via [smec@kuleuven.be](mailto:smec@kuleuven.be).
 *By clicking the ‘Continue’ button below, you are providing informed consent for the collection, storage, processing and analysis of your responses*.

End of Block: Landing page

Start of Block: Respondent Characteristics

C1 Over the last decade, what has been your main field of study?

- Arts and Humanities (e.g. History, Philosophy, Literature) (2)
- Life Sciences & Biomedical Sciences (e.g. Biology, Medicine, Dentistry) (5)
- Natural Sciences (e.g. Physics, Chemistry) (3)
- Social Sciences (e.g. Sociology, Psychology, Educational Sciences) (1)
- Technology & Engineering (e.g. Computer Science, Materials Science, Robotics) (4)

| 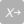 |
| --- |

C2 What was your main place of professional activity in the past decade?

- Africa (1)
- Asia (2)
- Australia (3)
- Europe (4)
- North America (5)
- Central or South America (6)

| Page Break |  |
| --- | --- |

C3 How long has it been since you obtained your PhD?

Display This Choice:

If Welcome to this survey concerning the ethical aspects of competitive research-funding. Its purpos... Is Displayed

- Less than 5 years (1)
- 0-10 years (2)
- 11-20 years (3)
- 21-30 years (4)
- 31-40 years (5)
- More than 40 years (6)

C4 The gender you identify with (multiple answers possible)

- Woman (1)
- Man (2)
- Nonbinary/Genderqueer (3)
- Prefer to self-identify(Specify if desired) (4) __________________________________________________
- I prefer not to say (5)

C5 In the past decade, in which of the following capacities have you participated in the research funding process? (*Check all that apply.*)

- **Reviewer of project proposals** that were submitted as part of an application for research funding (1)
- **Member of a panel** evaluating research-project proposals submitted as part of an application for research funding (2)
- **(Co-)author** of, **or contributor** to, a research-project proposal submitted as part of an application for research funding (3)
- ⊗None of the above (4)

| Page Break |  |
| --- | --- |

C6 Approximately, on how many research-grant applications were you **listed as PI or co-PI**in the **last decade**?

- None (4)
- Lessthan 2 (5)
- Between2 and 5 (6)
- Between6 and 10 (7)
- Between11 and 20 (8)
- Between21 and 50 (9)
- Morethan 50 (10)

| 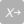 |
| --- |

C7 In the past decade and specifically in the context of grant writing or grant reviewing (i.e. not in the context of *conducting* research), how often have you observed others engage in **questionable practices or scientific misconduct**?

- Never (1)
- (2)
- (3)
- (4)
- (5)
- (6)
- Veryfrequently (7)

| 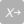 |
| --- |

C8 In the past decade and in the context of research funding, have you **alerted funders, an ombudsperson or another authority** to someone's behavior that you thought was questionable?

- Never (1)
- (2)
- (3)
- (4)
- (5)
- (6)
- Veryfrequently (7)

| 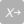 |
| --- |

C6_bis Have you, over the past 10 years, ever had too little funding to do meaningful research?

- Never (1)
- (2)
- (3)
- (4)
- (5)
- (6)
- Veryfrequently (7)

| Page Break |  |
| --- | --- |

A20
In the past decade, what was your **success rate** in securing grant funding (in terms of percentage)?

- Less than 10% (17)
- 10% - 20% (18)
- 20% - 30% (19)
- 30% - 40% (20)
- 40% - 50% (21)
- 50% - 75% (22)
- More than 75% (23)
- Not applicable (24)

End of Block: Respondent Characteristics

Start of Block: Applicants

A_intro The following questions concern your experiences (with the grant-funding process) as an **applicant for funding**.
 *(There are 20 questions in this section, grouped onto 7 screens)*

| 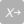 |
| --- |

A1 With regard to the **most recent funding application** you submitted, how **confident** were you that the project deliverables would be achieved if the funding were to be granted?

- Not at all (1)
- (2)
- (3)
- (4)
- (5)
- (6)
- Completely (7)

A2 In the past decade, how often have you **intentionally overstated your confidence** in your **predictions** (about, e.g., milestones, study results, impact) in a grant proposal?

- Never (1)
- (2)
- (3)
- (4)
- (5)
- (6)
- Almostalways (7)

| Page Break |  |
| --- | --- |

Tekst
Your experiences as an applicant - screen 2/7

| 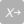 |
| --- |

A3
In the past decade, how often have you contributed to a project proposal that **did not list as an author** all those who contributed substantially, for example, because they were not eligible to apply for that grant?

- Never (1)
- (2)
- (3)
- (4)
- (5)
- (6)
- Almostalways (7)

A4 In the past decade, how often have you **hired external grant-writing consultants** to assist you in the preparation of your proposal?

- Never (1)
- (2)
- (3)
- (4)
- (5)
- (6)
- Almostalways (7)

| 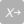 |
| --- |

A5 In the past decade, how often have you, in the final report of a funded project, included publications or other forms of output **not directly related** to the project?

- Never (1)
- (2)
- (3)
- (4)
- (5)
- (6)
- Almostalways (7)
- I did not have funded projectsin the past decade (8)

| Page Break |  |
| --- | --- |

Tekst
Your experiences as an applicant - screen 3/7

A6
In the past decade, how often have you requested **substantially more funding than what was required** for the research proposed in the grant application?

- Never (1)
- (2)
- (3)
- (4)
- (5)
- (6)
- Almostalways (7)

A7 In the past decade, how often have you requested **funding for new equipment** because it is more easily granted than funding for **maintenance**?

- Never (1)
- (2)
- (3)
- (4)
- (5)
- (6)
- Almostalways (7)
- I do not workwith expensiveequipment (8)

A8 In the past decade, how often have you **used project funding** for **purposes other than the research project** for which you received the funds?

- Never (1)
- (2)
- (3)
- (4)
- (5)
- (6)
- Almostalways (7)
- I did not have funded projectsin the past decade (8)

| 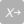 |
| --- |

A9 In the past decade, when there were **unused resources** at the end of a grant-funded research project, how often have you **spent these resources** on things that were **not related to** the project?

- Never (1)
- (2)
- (3)
- (4)
- (5)
- (6)
- Almostalways (7)
- I did not have funded projectsin the past decade (8)

| Page Break |  |
| --- | --- |

Tekst
Your experiences as an applicant - screen 4/7

| 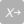 |
| --- |

A10
In the past decade, how often have you received funding for two separate grant proposals whose elements of planned research overlapped at least partially?

- Never (1)
- (2)
- (3)
- (4)
- (5)
- (6)
- Almostalways (7)

| 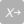 |
| --- |

A11 In the past decade and while preparing grant proposals, how often have you stated certain research to be **'planned for the future'**, when that research was, at least partially, **already in progress or finished**?
 (*Note*: this does not include pilot studies that are indicated as such in the application)

- Never (1)
- (2)
- (3)
- (4)
- (5)
- (6)
- Almostalways (7)

A12 In the past decade, how often have you applied for funding **primarily** to **improve your CV** or to **meet tenure or promotion criteria**?

- Never (1)
- (2)
- (3)
- (4)
- (5)
- (6)
- Almostalways (7)

A13 In the past decade, how often have you applied for funding **primarily** to **extend** the **employment** of **fellow researchers**?

- Never (1)
- (2)
- (3)
- (4)
- (5)
- (6)
- Almostalways (7)

| Page Break |  |
| --- | --- |

Tekst
Your experiences as an applicant - screen 5/7

A14
In the past decade, how often have you **intentionally cited works of potential referees** in order to improve your chances of securing a grant?

- Never (1)
- (2)
- (3)
- (4)
- (5)
- (6)
- Almostalways (7)

A15 In the past decade, how often have you **supplied the names of close colleagues or friends as suggested referees**?

- Never (1)
- (2)
- (3)
- (4)
- (5)
- (6)
- Almostalways (7)
- I did not applyfor such funding (8)

| Page Break |  |
| --- | --- |

Tekst
Your experiences as an applicant - screen 6/7

| 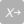 |
| --- |

A16
In the past decade, how often have you received a grant-review report which you thought **accurately reflected the quality of your grant proposal**?

- Never (1)
- (2)
- (3)
- (4)
- (5)
- (6)
- Almostalways (7)

A17 In the past decade, how often have you received a grant-review report that gave you the impression that the reviewer(s) did **not have the appropriate expertise** to evaluate the grant proposal?

- Never (1)
- (2)
- (3)
- (4)
- (5)
- (6)
- Almostalways (7)

A18 In the past decade, how often have you received a grant-review report which you thought was **unfair or biased** to your **disadvantage**?

- Never (1)
- (2)
- (3)
- (4)
- (5)
- (6)
- Almostalways (7)

A19 In the past decade, how often have you received a grant-review report which you thought was **unfair or biased** in your **favor**?

- Never (1)
- (2)
- (3)
- (4)
- (5)
- (6)
- Almostalways (7)

| Page Break |  |
| --- | --- |

Tekst
Your experiences as an applicant - screen 7/7

End of Block: Applicants

Start of Block: Reviewers

R_intro The following questions concern your experiences (with the grant-funding process) as a **reviewer of applications**.
 *(There are 8 questions in this section, grouped onto 3 screens)*

| 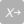 |
| --- |

R1 **On average**, how **confident** are you about the **reliability of your evaluations** of grant proposals?

- Not at all (1)
- (2)
- (3)
- (4)
- (5)
- (6)
- Completely (7)

| 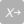 |
| --- |

R2 What do you do if, after accepting to review a proposal, you notice that you are **highly uncertain** about some or all of the judgements you have to make?

- You explicitly indicate this uncertainty in your review report (1)
- You decline to review the proposal (2)
- You only make judgements that you feel sufficiently certain about (3)
- You try to decrease uncertainty, e.g. by reading up about parts of the proposal you are not an expert in (4)
- You give the applicant the benefit of the doubt whenever you are uncertain (6)
- I inform the agency about this and ask for instructions (9)
- I do nothing in particular, high levels of uncertainty are simply part of the process (7)
- Other (please specify) (8) __________________________________________________

R3 In your field, how **likely** is it that your funding application would be reviewed by **reviewers** who have **both** the required **expertise** as well as **no conflicts of interest** with you or any other relevant stakeholder?

- Not at all (1)
- (2)
- (3)
- (4)
- (5)
- (6)
- Certain (7)

| Page Break |  |
| --- | --- |

Tekst
Your experiences as a reviewer - screen 2/3

R4
In the past decade, how often have you put **less time and effort** in reviewing a proposal than you thought **was required** to evaluate it?

- Never (1)
- (2)
- (3)
- (4)
- (5)
- (6)
- Almostalways (7)

R5 In the past decade, how often have you reviewed a proposal **without reading the entire application file**?

- Never (1)
- (2)
- (3)
- (4)
- (5)
- (6)
- Almostalways (7)

R6 In the past decade, how often have you **agreed to review** a project for which you **lacked the expertise** required to evaluate it?

- Never (1)
- (2)
- (3)
- (4)
- (5)
- (6)
- Almostalways (7)

| Page Break |  |
| --- | --- |

Tekst
Your experiences as a reviewer - screen 3/3

| 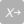 |
| --- |

R7
In the past decade, how often have you **reviewed** project proposals of either **close colleagues or friends**?

- Never (1)
- (2)
- (3)
- (4)
- (5)
- (6)
- Almostalways (7)

| 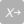 |
| --- |

R8
In the past decade, how often did you **intentionally** let **personal relationships** with, or **preferences** for or against, certain applicants **influence** your review reports of project proposals?

- Never (1)
- (2)
- (3)
- (4)
- (5)
- (6)
- Almostalways (7)

End of Block: Reviewers

Start of Block: Panellists

P_intro The following questions concern your experiences (with the grant-funding process) **as a panelist**.
 *(There are 6 questions in this section, grouped onto 2 screens)*

| 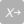 |
| --- |

P1 Did any of the funding organization(s) for which you were a panelist provide **instructions** related to **research integrity** in the context of grant writing and reviewing?

- None (1)
- (2)
- (3)
- (4)
- (5)
- (6)
- All of them (7)

| 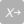 |
| --- |

P2 Did any of the funding organization(s) for which you were a panelist provide **training** in **research integrity** in the context of grant writing and reviewing?

- None (1)
- (2)
- (3)
- (4)
- (5)
- (6)
- All of them (7)

P3 In the past decade, how often were you obliged to **compare reviews or track records from different disciplines** without there being a clear common ground for such a comparison?

- Never (1)
- (2)
- (3)
- (4)
- (5)
- (6)
- Almostalways (7)

| Page Break |  |
| --- | --- |

Tekst
Your experiences as a panelist - screen 2/2

P4 In the past decade, how often did you go to panel meetings **less prepared** than you thought **was required** for the evaluation process?

- Never (1)
- (2)
- (3)
- (4)
- (5)
- (6)
- Almostalways (7)

P5 In the past decade, how often did you attend a panel meeting in which at least some of the panelists were **insufficiently prepared** or **lacked the expertise** required for the evaluation process?

- Never (1)
- (2)
- (3)
- (4)
- (5)
- (6)
- Almostalways (7)

P6 In the past decade, how often did you get the impression that there was **no substantial quality difference** between the bottom 10% of projects that were **selected** by your panel and the top 10% of projects that were **not selected**?

- Never (1)
- (2)
- (3)
- (4)
- (5)
- (6)
- Almostalways (7)

End of Block: Panellists

Start of Block: CoI

CoI One final question: in which of the following cases do you think there is a **potential conflict of interest** that should rule you out as a reviewer? (*Tick all that apply*)

- You are asked to review a proposal of a researcher who you know informally (1)
- You are asked to review a proposal of a researcher who is close friends with people who you know well (2)
- You are asked to review a proposal of a researcher with whom you have published in the past (3)
- You are asked to review a proposal of a researcher who used to be your colleague but is now at another institution (4)
- You are asked to review a proposal of a researcher you meet regularly at conferences (5)
- You are asked to review a proposal of a researcher you dislike as a person (6)
- You are asked to review a proposal of a researcher whose superior you know very well (7)

| Page Break |  |
| --- | --- |

Q57 You have now reached the end of the survey. Thank you very much for your input!

Is there anything else you'd like to add? Feel free to write your comments in the field below.

________________________________________________________________

________________________________________________________________

________________________________________________________________

________________________________________________________________

________________________________________________________________

End of Block: CoI
