# Supplementary material for: Questionable research practices in competitive grant funding: A survey
Source: PLoS One. 2023 Nov 2;18(11):e0293310. doi: 10.1371/journal.pone.0293310 (PMC10621923; doi:10.1371/journal.pone.0293310)
Supplement: S2 File — Email sent out by FWO to invite researchers to participate in the survey. (DOCX) [file pone.0293310.s014.docx]

**S2 File. Invitation messages for participation in the survey**

1. FWO newsletter (Dutch)

**Steun een goed doel met deze bevraging over wetenschappelijke integriteit**

Er is de laatste jaren veel onderzoek gedaan naar wetenschappelijke integriteit, inbreuken op integriteit, en wat deze inbreuken veroorzaakt. Dit onderzoek richt zich vooral op integriteit in de context van het ontwerpen, interpreteren en publiceren van wetenschappelijk onderzoek. Hoewel de financiering van onderzoek een cruciaal deel vormt van het wetenschappelijk proces, is er weinig bekend over wetenschappelijke integriteit in die context. Klik [hier](https://kuleuven.eu.qualtrics.com/jfe/form/SV_09w6QK504VT5ds2) om deel te nemen aan een korte (mediaan pilot: 11 minuten), volledig anonieme bevraging over wetenschappelijke integriteit in de context van het schrijven en reviewen van onderzoeksprojecten. Help hiermee de wetenschap vooruit, maar ook de strijd tegen malaria: per volledig ingevulde bevraging storten we €1 aan [‘Malaria Consortium’](https://www.malariaconsortium.org/) voor de aankoop van medicijnen die malaria voorkomen.

1. FWO newsletter (English)

**Support charity with this survey about research integrity**

There has been a surge of interest in research integrity over the last decade. This research primarily focuses on research integrity in the context of designing, interpreting and publishing scientific research. Even though research funding is a crucial part of the research process, very little is known about research integrity in that context. Click [here](https://kuleuven.eu.qualtrics.com/jfe/form/SV_09w6QK504VT5ds2) to participate in a brief (pilot median: 11 minutes) survey assessing the prevalence of unethical practices related to research funding and some of their drivers, targeted to the role(s) you have taken up (i.e., applicant, reviewer, and/or panelist). By participating you’ll help grow our understanding of research integrity as well as reduce the worldwide impact of malaria: for each completed survey, **we will donate €1** to the [Malaria Consortium](https://www.malariaconsortium.org/).

1. FWO/Science Direct email

Dear researcher

There has been a surge of interest in **research integrity** over the last decade. Research on this topic typically focuses on research design, data collection and publication practices. **Funding and grant-reviewing practices**, on the other hand, have remained largely understudied. This is unfortunate, as these constitute an important part of researchers' activities.

Some tentative evidence indicates that both applicants and reviewers do not always act in line with commonly accepted codes of conduct ([Anderson et al. 2007](https://journals.lww.com/academicmedicine/Fulltext/2007/09000/What_Do_Mentoring_and_Training_in_the_Responsible.7.aspx); [Bouter et al. 2016](https://researchintegrityjournal.biomedcentral.com/articles/10.1186/s41073-016-0024-5); see also [Conix, De Block, and Vaesen 2021](https://f1000research.com/articles/10-1126)). Grants may be written by postdocs but submitted by PIs; funding may be requested based on work already done. Similarly, peer reviewers may not disclose potential conflicts of interest, review grants outside their area of expertise, or take ideas from the grants they review.

With this email we kindly invite you to participate in **a fully anonymous survey** assessing the prevalence of unethical practices related to research funding and some of their drivers, targeted to the role(s) you have taken up (i.e., applicant, reviewer, and/or panelist). It should take you around 10 minutes to answer the questions.

By participating you’ll help grow our understanding of research integrity as well as reduce the worldwide impact of malaria: for each completed survey, **we will donate €1** to the [Malaria Consortium](https://www.malariaconsortium.org/), which [givewell.org](https://www.givewell.org/) currently lists as one of the most effective charities..

Click the following link to start the survey: [Take the Survey](https://kuleuven.eu.qualtrics.com/jfe/form/SV_09w6QK504VT5ds2)

Or paste this link into your browser: <https://kuleuven.eu.qualtrics.com/jfe/form/SV_09w6QK504VT5ds2>

Should you have any queries or concerns regarding the study, please feel free to email Dr. Stijn Conix ([stijn.conix@kuleuven.be](mailto:stijn.conix@kuleuven.be)) in confidence. The study has been approved by the [Social and Societal Ethics Committee of KU Leuven](https://www.kuleuven.be/english/research/ethics/committees/smec) (file G-2021-4529-R2(MIN)).

With kind regards,

Prof. Andreas De Block, KU Leuven

Prof. Krist Vaesen, Eindhoven University of Technology

Dr. Stijn Conix, KU Leuven

Dr. Steven De Peuter, KU Leuven
