# Supplementary material for: Questionable research practices in competitive grant funding: A survey
Source: PLoS One. 2023 Nov 2;18(11):e0293310. doi: 10.1371/journal.pone.0293310 (PMC10621923; doi:10.1371/journal.pone.0293310)
Supplement: S3 File — The code can also be accessed through the OSF page of the project (https://osf.io/jk6wd/). (ZIP) [file pone.0293310.s015.zip › S13 File/ERF_field_exploration_upload.html]

ERF\_field\_exploration\_upload


In [3]:

```
import pandas as pd
import numpy as np

# for the stats
import pymc as pm
import bambi as bmb
from scipy.special import expit as logistic
# import aesara.tensor as at

# for plotting
import seaborn as sns
import matplotlib.pyplot as plt
import arviz as az

# for DAGs

import collections.abc
#causalgraphicalmodels needs the four following aliases to be done manually.
collections.Iterable = collections.abc.Iterable
collections.Mapping = collections.abc.Mapping
collections.MutableSet = collections.abc.MutableSet
collections.MutableMapping = collections.abc.MutableMapping
from causalgraphicalmodels import CausalGraphicalModel
import daft
```

In [4]:

```
# versions

print('\n'.join(f'{m.__name__}=={m.__version__}' for m in globals().values() if getattr(m, '__version__', None)))
```

```
pandas==1.5.3
numpy==1.24.2
pymc==5.1.2
bambi==0.10.0
seaborn==0.12.2
arviz==0.15.1
daft==0.1.2
```

In [5]:

```
sns.set_palette("Dark2")
sns.set_style("darkgrid")
sns.set_context("paper", font_scale=1.5)
```

In [6]:

```
# seed

SEED = 2808

np.random.seed(SEED)
```

# Hypothesis¶

Preious work finds that QRPs are more prevalent in the biomedical sciences. We check whether that is also the case in our sample

# DAG¶

In [7]:

```
dag_field = CausalGraphicalModel(nodes=["QRP", "G", "Age",'Field', 'Ind','Q'], 
                              edges=[("Age", "QRP"), ("Age", "QRP"), ("Field", "QRP"), 
                                     ('G','Age'),('G','QRP'),('G','Field'), ('Age','Field'),
                                    ('G','Ind'),('Field','Ind'),('Age','Ind'), ('Ind','QRP'),
                                    ('Q','QRP')])
dag_field.draw()
```

Out[7]:

Q

Q


QRP

QRP


Q->QRP


Age

Age


Age->QRP


Field

Field


Age->Field


Ind

Ind


Age->Ind


Field->QRP


Field->Ind


Ind->QRP


G

G


G->QRP


G->Age


G->Field


G->Ind

In [6]:

```
def backdoor(dag, predictor, outcome):
    all_adjustment_sets = dag.get_all_backdoor_adjustment_sets(predictor, outcome)
    for s in all_adjustment_sets:
        if all(not t.issubset(s) for t in all_adjustment_sets if t != s):
            if s != {"U"}:
                print(s)

                
# For gender

backdoor(dag_field,'Field','QRP')
```

```
frozenset({'G', 'Age'})
```

note: We include gender and seniority as controls, but also add question type as competing cause.

# Indices & Data¶

In [7]:

```
# data

real_data = pd.read_csv('...')

# qrp columns
r_qrp = ['R4', 'R5', 'R6','R7','R8']
a_qrp = ['A2','A3','A5', 'A6','A8', 'A9', 'A10', 'A11','A14','A15']
p_qrp = ['P4']
demo = ['C1', 'C3', 'C4']

# select relevant columns

df = real_data[r_qrp + p_qrp + a_qrp + demo].reset_index().copy()

# put in long form

df = df.melt(id_vars= demo + ['index'])
df.columns = ['field','seniority','gender','ind','question','score']

# drop other genders

other_genders = len((df.loc[(df.gender != 'male') & (df.gender != 'female')]))
df = df.loc[(df.gender == 'male') | (df.gender == 'female')]

print(f'number of responses removed: {other_genders}')

# let score start at 0, replace NA by 0, and make dtype categorical
df['score'] = df.score - 1
df[['field','seniority','gender','ind','question']] = df[['field','seniority','gender','ind','question']].astype('category')
df = df.replace({7:0})

#complete case analyses: dropna

predrop = len(df)
df = df.dropna()
postdrop = len(df)
print(f'rows with nans removed: {predrop - postdrop}')
print(f'total responses: {postdrop}')


df.head()
```

```
number of responses removed: 336
rows with nans removed: 2088
total responses: 8840
```

Out[7]:

|  | field | seniority | gender | ind | question | score |
| --- | --- | --- | --- | --- | --- | --- |
| 1 | Arts & Hum | 11-20 | female | 1 | R4 | 1.0 |
| 2 | Life & Biomed | 21-30 | female | 2 | R4 | 1.0 |
| 3 | Arts & Hum | 21-30 | female | 3 | R4 | 0.0 |
| 4 | Arts & Hum | 21-30 | male | 4 | R4 | 0.0 |
| 5 | Social Science | 11-20 | male | 5 | R4 | 1.0 |

In [9]:

```
# data from how many respondents

len(df.ind.unique())
```

Out[9]:

```
678
```

In [11]:

```
# data for pymc model

q_idx = df.question.cat.codes.values
s_idx = df.seniority.cat.codes.values
g_idx = df.gender.cat.codes.values
i_idx = df.ind.cat.codes.values
f_idx = df.field.cat.codes.values


# coordinates
q_codes = df.question.cat.categories.values
s_codes = df.seniority.cat.categories.values
g_codes = df.gender.cat.categories.values
i_codes = df.ind.cat.categories.values
f_codes = df.field.cat.categories.values
cutpoint_codes = np.array(['cutpoint_1','cutpoint_2','cutpoint_3','cutpoint_4','cutpoint_5','cutpoint_6'])


coords = {'q_n':q_codes, 's_n':s_codes,'g_n':g_codes,'i_n':i_codes,
          'f_n':f_codes, 'c_n':cutpoint_codes}
```

# Total field effect¶

In [17]:

```
with pm.Model(coords=coords) as field_check:

    # data

    G = pm.MutableData("G", g_idx)
    F = pm.MutableData("F", f_idx)
    S = pm.MutableData("S", s_idx)
    Q = pm.MutableData("Q", q_idx)

    # fixed hyperpriors for field, seniority and participant
    
    s_field = pm.Uniform('s_field', 0,3)
    s_seniority = pm.Uniform('s_seniority',0,3)
    
    # prior for the cutpoints, one set of cutpoints per question
    
    cutpoints = pm.Normal('cutpoints',
                           mu=[0,1,2,3,4,5],
                           sigma=1,
                           transform=pm.distributions.transforms.univariate_ordered,
                           dims = ('q_n','c_n')
                           )

    # variable priors for the demographic predictors
    # non-centered to make sampling easier
    
    gender = pm.Normal("gender", 0.0, 1, dims = 'g_n')
    
    z_field = pm.Normal("z_field", 0.0, 1, dims = 'f_n')
    field = pm.Deterministic("field", z_field * s_field, dims = 'f_n')
    
    z_seniority = pm.Normal("z_seniority", 0.0, 1.0, dims = 's_n')
    seniority = pm.Deterministic("seniority", z_seniority * s_seniority, dims = 's_n')

    phi = gender[G] + field[F] + seniority[S] # 

    y = pm.OrderedLogistic("y", phi, cutpoints[Q], observed=df.score,compute_p = False)
    
    pr = pm.sample_prior_predictive()
```

```
Sampling: [cutpoints, gender, s_field, s_seniority, y, z_field, z_seniority]
```

In [18]:

```
# plot the priors

variables = ['cutpoints','gender','s_field','s_seniority','field','seniority']
fig, axs = plt.subplots(2,3,figsize = (15,7))

for ax, var in zip(axs.flat, variables):
    if len(pr.prior[var].shape) > 2:
        az.plot_posterior(pr.prior[var][:,:,0], ax=ax)
    else:
        az.plot_posterior(pr.prior[var][:,:], ax=ax)
```

In [19]:

```
# sample from the posterior

with field_check:
    trace = pm.sample(5000,
                      tune = 1000,
                      return_inferencedata = True,
                      idata_kwargs={"log_likelihood": True},
                      random_seed = SEED,
                      target_accept = 0.99)
```

```
Auto-assigning NUTS sampler...
Initializing NUTS using jitter+adapt_diag...
Multiprocess sampling (4 chains in 4 jobs)
NUTS: [s_field, s_seniority, cutpoints, gender, z_field, z_seniority]
```

100.00% [24000/24000 1:48:56<00:00 Sampling 4 chains, 0 divergences]

```
Sampling 4 chains for 1_000 tune and 5_000 draw iterations (4_000 + 20_000 draws total) took 6585 seconds.
```

In [8]:

```
# save the trace for later use

trace.to_netcdf('...')
```

In [27]:

```
az.summary(trace, var_names = ['gender','s_field','s_seniority','field','seniority'])
```

Out[27]:

|  | mean | sd | hdi\_3% | hdi\_97% | mcse\_mean | mcse\_sd | ess\_bulk | ess\_tail | r\_hat |
| --- | --- | --- | --- | --- | --- | --- | --- | --- | --- |
| gender[female] | -0.210 | 0.209 | -0.583 | 0.201 | 0.003 | 0.002 | 3865.0 | 6849.0 | 1.0 |
| gender[male] | -0.266 | 0.209 | -0.669 | 0.111 | 0.003 | 0.002 | 3851.0 | 6839.0 | 1.0 |
| s\_field | 0.346 | 0.210 | 0.108 | 0.695 | 0.003 | 0.002 | 6355.0 | 7635.0 | 1.0 |
| s\_seniority | 0.134 | 0.104 | 0.022 | 0.290 | 0.001 | 0.001 | 6837.0 | 9701.0 | 1.0 |
| field[Arts & Hum] | -0.288 | 0.173 | -0.631 | 0.025 | 0.002 | 0.002 | 6640.0 | 8375.0 | 1.0 |
| field[Life & Biomed] | 0.319 | 0.168 | 0.007 | 0.644 | 0.002 | 0.002 | 6508.0 | 7450.0 | 1.0 |
| field[Natural Science] | -0.027 | 0.171 | -0.353 | 0.292 | 0.002 | 0.002 | 6694.0 | 7643.0 | 1.0 |
| field[Social Science] | -0.005 | 0.170 | -0.319 | 0.327 | 0.002 | 0.002 | 6618.0 | 7625.0 | 1.0 |
| field[Tech & Engineering] | -0.061 | 0.173 | -0.383 | 0.271 | 0.002 | 0.002 | 6724.0 | 7786.0 | 1.0 |
| seniority[0-10] | -0.054 | 0.091 | -0.228 | 0.103 | 0.001 | 0.001 | 14402.0 | 13886.0 | 1.0 |
| seniority[11-20] | 0.126 | 0.085 | -0.010 | 0.286 | 0.001 | 0.001 | 12607.0 | 11768.0 | 1.0 |
| seniority[21-30] | -0.044 | 0.082 | -0.197 | 0.101 | 0.001 | 0.001 | 13135.0 | 13038.0 | 1.0 |
| seniority[31-40] | -0.025 | 0.084 | -0.184 | 0.120 | 0.001 | 0.001 | 13893.0 | 13462.0 | 1.0 |
| seniority[>40] | -0.012 | 0.089 | -0.167 | 0.155 | 0.001 | 0.001 | 14811.0 | 13074.0 | 1.0 |

## sampling stats¶

In [28]:

```
#R-hat (we used three chains) and ESS

az.summary(trace, var_names = ['gender','field','seniority', 's_field', 's_seniority'])[['r_hat', 'ess_bulk']].T
```

Out[28]:

|  | gender[female] | gender[male] | field[Arts & Hum] | field[Life & Biomed] | field[Natural Science] | field[Social Science] | field[Tech & Engineering] | seniority[0-10] | seniority[11-20] | seniority[21-30] | seniority[31-40] | seniority[>40] | s\_field | s\_seniority |
| --- | --- | --- | --- | --- | --- | --- | --- | --- | --- | --- | --- | --- | --- | --- |
| r\_hat | 1.0 | 1.0 | 1.0 | 1.0 | 1.0 | 1.0 | 1.0 | 1.0 | 1.0 | 1.0 | 1.0 | 1.0 | 1.0 | 1.0 |
| ess\_bulk | 3865.0 | 3851.0 | 6640.0 | 6508.0 | 6694.0 | 6618.0 | 6724.0 | 14402.0 | 12607.0 | 13135.0 | 13893.0 | 14811.0 | 6355.0 | 6837.0 |

In [29]:

```
#see here: https://docs.pymc.io/en/v3/pymc-examples/examples/diagnostics_and_criticism/sampler-stats.html

#print number of divergences, ideally 0
print(f'divergences: {trace.sample_stats["diverging"].values.sum()}')

#print the acceptance rate
print(f'mean acceptance rate: {trace.sample_stats["acceptance_rate"].values.mean()}')

#compare the overall distribution of the energy levels with the change of energy between successive samples. Ideally, they should be very similar
az.plot_energy(trace, figsize=(6, 4));
```

```
divergences: 0
mean acceptance rate: 0.9863283519483408
```

## Results¶

In [30]:

```
az.plot_forest(trace, var_names = 'field', combined = True)
```

Out[30]:

```
array([<Axes: title={'center': '94.0% HDI'}>], dtype=object)
```

In [26]:

```
# plot difference between coefficients for biomedical sciences and the other fields
fig, axs = plt.subplot_mosaic("ABCD;EEEE",figsize = (15,8))

fields = list(trace.posterior['f_n'].values)
fields.remove('Life & Biomed')
biomed = trace.posterior['field'].loc[:,:,'Life & Biomed']

# for ax, field in zip(axs.flat[-1], fields):
az.plot_posterior(biomed - trace.posterior['field'].loc[:,:,'Arts & Hum'], ax=axs['A'])
axs['A'].set_title('Arts & Hum')

az.plot_posterior(biomed - trace.posterior['field'].loc[:,:,'Natural Science'], ax=axs['B'])
axs['B'].set_title('Natural Science')

az.plot_posterior(biomed - trace.posterior['field'].loc[:,:,'Social Science'], ax=axs['C'])
axs['C'].set_title('Social Science')

az.plot_posterior(biomed - trace.posterior['field'].loc[:,:,'Tech & Engineering'], ax=axs['D'])
axs['D'].set_title('Tech & Engineering')

sns.countplot(data = ppcdf.stack().reset_index().rename(columns = {0:'QRP', 'level_1':'Field'}),
              x = 'QRP',
              hue = 'Field',
             ax=axs['E'])

axs['E'].set_xticklabels(['Never',2,3,4,5,6,'Almost always'])
axs['E'].set_ylabel('Posterior predictive count')

plt.suptitle('Differences between fields in QRP item responses', fontsize = 20)
    

plt.savefig('...', dpi = 300)

plt.show()
```

In [22]:

```
# generate posterior predictive samples
# we generate them for the entire sample set to each of the fields (keeping other variables intact)

ppcs = {}
with field_check:
    for i in range(5):
        pm.set_data({"F": np.repeat(i, len(df))})
        ppc = pm.sample_posterior_predictive(trace, progressbar = False)
        ppcs[i] = ppc
```

```
Sampling: [y]
Sampling: [y]
Sampling: [y]
Sampling: [y]
Sampling: [y]
```

In [25]:

```
#posterior predictive counts of the various qrp response options

# I take a random chain and draws

fields = list(trace.posterior['f_n'].values)
              
ppcs_dct = {fields[i]:np.random.choice(np.ravel(ppcs[i].posterior_predictive['y'].values),size = 20000, replace=True) for i in range(5)}

ppcdf = pd.DataFrame(list(ppcs_dct.values()), index = fields).T

sns.countplot(data = ppcdf.stack().reset_index().rename(columns = {0:'QRP', 'level_1':'Field'}),
              x = 'QRP',
              hue = 'Field')

plt.show()
```

In [ ]:

```

```
