# Supplementary material for: Questionable research practices in competitive grant funding: A survey
Source: PLoS One. 2023 Nov 2;18(11):e0293310. doi: 10.1371/journal.pone.0293310 (PMC10621923; doi:10.1371/journal.pone.0293310)
Supplement: S3 File — The code can also be accessed through the OSF page of the project (https://osf.io/jk6wd/). (ZIP) [file pone.0293310.s015.zip › S13 File/ERF_gender_hypothesis1_upload.html]

ERF\_gender\_hypothesis1\_upload


In [1]:

```
import pandas as pd
import numpy as np
import pickle

# for the stats
import pymc as pm
import bambi as bmb
from scipy.special import expit as logistic
# import aesara.tensor as at

# for plotting
import seaborn as sns
import matplotlib.pyplot as plt
import arviz as az

# for DAGs

import collections.abc
#causalgraphicalmodels needs the four following aliases to be done manually.
collections.Iterable = collections.abc.Iterable
collections.Mapping = collections.abc.Mapping
collections.MutableSet = collections.abc.MutableSet
collections.MutableMapping = collections.abc.MutableMapping
from causalgraphicalmodels import CausalGraphicalModel
import daft
```

In [2]:

```
# versions

print('\n'.join(f'{m.__name__}=={m.__version__}' for m in globals().values() if getattr(m, '__version__', None)))
```

```
pandas==1.5.3
numpy==1.24.2
pymc==5.1.2
bambi==0.10.0
seaborn==0.12.2
arviz==0.15.1
daft==0.1.2
```

In [3]:

```
sns.set_palette("Dark2")
sns.set_style("darkgrid")
sns.set_context("paper", font_scale=1.5)
```

In [4]:

```
# seed

SEED = 2808

np.random.seed(SEED)
```

# Hypothesis¶

## Description¶

**GENDER HYPOTHESIS 1: Responses to individual qrp questions tend to be higher (on a scale 1 - 7) if the responder identifies as male than if the respondent identies as female'.**

note: In the preregistration we had written that we would leave panelist qrps out of this analysis as it would force us to omit from the data any respondent that hasn't filled in the 'panelist' questions. However, this is not true, as this is an item-level outcome variable. Hence, we have included all responses, regardless of which roles the respondents filled in. This has not affected the result in a substantial way.

## Reporting¶

In the preregistered analysis code, we had written that we would report model 1 in the paper. However, seeing that, like in the pilot, the influence of question type and individual is much larger than that of gender, we think it better to report the model that includes these in the paper. The other models do not give different results with respect to the hypothesis (i.e. no effect for gender), and are still accessible through the OSF page of the project.

## Justification¶

We expected higher qrp levels for those who identify as male on the basis of:

- the pilot study (see results below, generally a weak effect of 'male' increasing the probability of higher ordinal responses.
- existing research: Fang et al. 2013 doi: 10.1128/mBio.00640-12 find that in reports of scientific misconduct (fraud, fabrication, and plagiarism) the proportion of males is higher than the proportion of males in the relevant disciplines. In line with this, Gopalakrishna et al 2022 doi: 10.1371/journal.pone.0263023 find that being a male increased the odds of various qrps. While one other study did not find this in an analysis of 120 cases of wrongdoing (of which 40 FFP and 40 other research misconduct), we think the evidence in favour of this hypothesis is stronger, in particular due to the much larger sample size and scope of the Gopalakrishna study, and the fact that the results are weakly confirmed by our pilot study.
- We choose to compare 'male' with 'female' instead of 'non-male' (which includes nonbinary and other) because potential explanations involve societal roles of men/women and levels of testosterone. These explanations are easier to evaluate if the hypothesis focuses on men and women.
- field and career stage are included in the DAG because previous research suggests they might play a role. We did not include 'continent' because we have no reason (neither pilot results nor previous research) to think it is relevant. In addition, our samples will be mostly european, meaning that we'll have too little data from other continents for meaningfull comparison.

# DAG¶

In [5]:

```
# draw the dag that expresses our causal assumptions

dag_gender = CausalGraphicalModel(nodes=["QRP", "Gender", "Seniority",'Field', 'Respondent','Question'], 
                              edges=[("Seniority", "QRP"), ("Seniority", "QRP"), ("Field", "QRP"), 
                                     ('Gender','Seniority'),('Gender','QRP'),('Gender','Field'), ('Seniority','Field'),
                                    ('Gender','Respondent'),('Field','Respondent'),('Seniority','Respondent'), ('Respondent','QRP'),
                                    ('Question','QRP')])
dag_gender.draw()
```

Out[5]:

Seniority

Seniority


Respondent

Respondent


Seniority->Respondent


QRP

QRP


Seniority->QRP


Field

Field


Seniority->Field


Respondent->QRP


Question

Question


Question->QRP


Gender

Gender


Gender->Seniority


Gender->Respondent


Gender->QRP


Gender->Field


Field->Respondent


Field->QRP

In [6]:

```
# draw the same dag for the paper, now in better quality

def draw_dag(dag, coordinates):
    pgm = daft.PGM(grid_unit = 3, node_unit = 3, aspect = 2, dpi = 1200)
    for node in dag.dag.nodes:
        pgm.add_node(node, node, *coordinates[node], fontsize = 30)
    for edge in dag.dag.edges:
        pgm.add_edge(*edge)
    pgm.render()
    plt.savefig('...', dpi = 1200)


coordinates = {"QRP": (4, 2), "Question": (6, 4), "Respondent": (-4,2), 
               "Field": (-4, 6),'Seniority':(1,5), 'Gender':(2,8)}

draw_dag(dag_gender, coordinates)
```

In [7]:

```
# a function to identify backdoor paths, and check for gender (our variable of interest)

def backdoor(dag, predictor, outcome):
    all_adjustment_sets = dag.get_all_backdoor_adjustment_sets(predictor, outcome)
    for s in all_adjustment_sets:
        if all(not t.issubset(s) for t in all_adjustment_sets if t != s):
            if s != {"U"}:
                print(s)

                
# For gender

backdoor(dag_gender,'Gender','QRP')
```

```
frozenset()
```

note: We are interested in the direct effect of gender. Hence, we will include seniority, field, individual as controls and gender variable of interest. We will also include question as a competing cause. This model will give us an estimate of the direct effect of gender, the direct effect of individuals, and the effect of questions.

# Indices & Data¶

In [8]:

```
# data has been processed already in ERF_get_data.
# Now make it into the right shape for this test.

real_data = pd.read_csv('...')

# lists with columns that are QRP questions

r_qrp = ['R4', 'R5', 'R6','R7','R8']
a_qrp = ['A2','A3','A5', 'A6','A8', 'A9', 'A10', 'A11','A14','A15']
p_qrp = ['P4']
demo = ['C1', 'C3', 'C4']

# select the columns that we need

df = real_data[r_qrp + p_qrp + a_qrp + demo].reset_index().copy()

df = df.melt(id_vars= demo + ['index'])
df.columns = ['field','seniority','gender','ind','question','score']

# drop other genders

other_genders = len((df.loc[(df.gender != 'male') & (df.gender != 'female')]))
df = df.loc[(df.gender == 'male') | (df.gender == 'female')]

print(f'number of responses removed: {other_genders}')
df['score'] = df.score - 1
df[['field','seniority','gender','ind','question']] = df[['field','seniority','gender','ind','question']].astype('category')
df = df.replace({7:0})

#complete case analyses: dropna

predrop = len(df)
df = df.dropna()
postdrop = len(df)
print(f'rows with nans removed: {predrop - postdrop}')
print(f'total responses: {postdrop}')


df.head()
```

```
number of responses removed: 336
rows with nans removed: 2088
total responses: 8840
```

Out[8]:

|  | field | seniority | gender | ind | question | score |
| --- | --- | --- | --- | --- | --- | --- |
| 1 | Arts & Hum | 11-20 | female | 1 | R4 | 1.0 |
| 2 | Life & Biomed | 21-30 | female | 2 | R4 | 1.0 |
| 3 | Arts & Hum | 21-30 | female | 3 | R4 | 0.0 |
| 4 | Arts & Hum | 21-30 | male | 4 | R4 | 0.0 |
| 5 | Social Science | 11-20 | male | 5 | R4 | 1.0 |

In [9]:

```
# data from how many respondents

len(df.ind.unique())
```

Out[9]:

```
678
```

In [10]:

```
#  data for pymc model
q_idx = df.question.cat.codes.values
s_idx = df.seniority.cat.codes.values
g_idx = df.gender.cat.codes.values
i_idx = df.ind.cat.codes.values
f_idx = df.field.cat.codes.values

# coordinates for the pymc model
q_codes = df.question.cat.categories.values
s_codes = df.seniority.cat.categories.values
g_codes = df.gender.cat.categories.values
i_codes = df.ind.cat.categories.values
f_codes = df.field.cat.categories.values
cutpoint_codes = np.array(['cutpoint_1','cutpoint_2','cutpoint_3','cutpoint_4','cutpoint_5','cutpoint_6'])


# dict with coordinates to put into the model
coords = {'q_n':q_codes, 's_n':s_codes,'g_n':g_codes,'i_n':i_codes,
          'f_n':f_codes, 'c_n':cutpoint_codes}
```

# Total effect of gender¶

This is not preregistered, and we will not report it in the paper, but just for interest: the total effect of gender.

In [22]:

```
with pm.Model(coords=coords) as gender_hyp1_total:

    # data

    G = pm.MutableData("G", g_idx)
    Q = pm.MutableData("Q", q_idx)

    
    # prior for the cutpoints, one set of cutpoints per question
    
    cutpoints = pm.Normal('cutpoints',
                           mu=[0,1,2,3,4,5],
                           sigma=1,
                           transform=pm.distributions.transforms.univariate_ordered,
                           dims = ('q_n','c_n')
                           )
    
    # prior for gender
    
    gender = pm.Normal("gender", 0.0, 1, dims = 'g_n')
    
  

    phi = gender[G] 
    
    # don't compute and store in the trace the inferred probabilities of each categories, based on the cutpoints’ values
    # it takes too much memory; set compute_p to False to store them.

    y = pm.OrderedLogistic("y", phi, cutpoints[Q], observed=df.score,compute_p = False)
    
    pr = pm.sample_prior_predictive()
```

```
Sampling: [cutpoints, gender, y]
```

In [23]:

```
# get the shape of the posterior by sampling from it

with gender_hyp1_total:
    trace_total = pm.sample(10000,
                            tune = 1000,
                            return_inferencedata = True,
                            random_seed = SEED,
                            target_accept = 0.8)
```

```
Auto-assigning NUTS sampler...
Initializing NUTS using jitter+adapt_diag...
Multiprocess sampling (4 chains in 4 jobs)
NUTS: [cutpoints, gender]
```

100.00% [44000/44000 20:30<00:00 Sampling 4 chains, 0 divergences]

```
Sampling 4 chains for 1_000 tune and 10_000 draw iterations (4_000 + 40_000 draws total) took 1252 seconds.
```

In [11]:

```
# save the trace for later use

# trace_total.to_netcdf('...')

# to load

trace_total = az.from_netcdf('...')
```

In [24]:

```
az.summary(trace_total)
```

Out[24]:

|  | mean | sd | hdi\_3% | hdi\_97% | mcse\_mean | mcse\_sd | ess\_bulk | ess\_tail | r\_hat |
| --- | --- | --- | --- | --- | --- | --- | --- | --- | --- |
| gender[female] | -0.123 | 0.111 | -0.335 | 0.083 | 0.001 | 0.001 | 7062.0 | 13968.0 | 1.0 |
| gender[male] | -0.168 | 0.110 | -0.373 | 0.037 | 0.001 | 0.001 | 6987.0 | 14003.0 | 1.0 |
| cutpoints[A10, cutpoint\_1] | -0.081 | 0.137 | -0.343 | 0.173 | 0.001 | 0.001 | 9959.0 | 19756.0 | 1.0 |
| cutpoints[A10, cutpoint\_2] | 1.248 | 0.150 | 0.971 | 1.535 | 0.001 | 0.001 | 12020.0 | 23414.0 | 1.0 |
| cutpoints[A10, cutpoint\_3] | 1.942 | 0.169 | 1.622 | 2.256 | 0.001 | 0.001 | 14940.0 | 25714.0 | 1.0 |
| ... | ... | ... | ... | ... | ... | ... | ... | ... | ... |
| cutpoints[R8, cutpoint\_2] | 2.784 | 0.196 | 2.425 | 3.158 | 0.001 | 0.001 | 20987.0 | 27757.0 | 1.0 |
| cutpoints[R8, cutpoint\_3] | 3.703 | 0.259 | 3.226 | 4.198 | 0.001 | 0.001 | 34745.0 | 31024.0 | 1.0 |
| cutpoints[R8, cutpoint\_4] | 4.800 | 0.354 | 4.155 | 5.483 | 0.002 | 0.001 | 54995.0 | 33116.0 | 1.0 |
| cutpoints[R8, cutpoint\_5] | 6.118 | 0.481 | 5.223 | 7.021 | 0.002 | 0.001 | 62873.0 | 33868.0 | 1.0 |
| cutpoints[R8, cutpoint\_6] | 8.006 | 0.682 | 6.745 | 9.288 | 0.003 | 0.002 | 65395.0 | 32353.0 | 1.0 |

98 rows × 9 columns

In [26]:

```
# plot difference between coefficients for female and male

fig, ax = plt.subplots(figsize = (6,3))

male = trace_total.posterior['gender'].loc[:,:,'male']
female = trace_total.posterior['gender'].loc[:,:,'female']

az.plot_posterior(female-male, ax=ax)
ax.set_title('Difference in coefficients for female and male')

plt.show()
```

In [30]:

```
# generate posterior predictive samples
# we generate them for the entire sample set to male (other characteristics intact) and female (idem)

ppcs = {}
with gender_hyp1_total:
    for i in range(2):
        pm.set_data({"G": np.repeat(i, len(df))})
#         thinned_trace = trace_total.sel(draw=slice(None, None, 5))
        ppc = pm.sample_posterior_predictive(trace_total, progressbar = True)
        ppcs[i] = ppc
```

```
Sampling: [y]
```

100.00% [40000/40000 45:01<00:00]

```
Sampling: [y]
```

100.00% [40000/40000 39:02<00:00]

In [31]:

```
#posterior predictive counts of the various success categories for qrp vs no_qrp

# randomly draw 20k samples from the ones  generated

female = np.random.choice(np.ravel(ppcs[0].posterior_predictive['y'].values),
                          size = 20000,
                          replace=True)

male = np.random.choice(np.ravel(ppcs[1].posterior_predictive['y'].values),
                        size = 20000,
                        replace=True)

ppcdf = pd.DataFrame([female, male], index = ['female', 'male']).T

sns.countplot(data = ppcdf.stack().reset_index().rename(columns = {0:'QRP', 'level_1':'Gender'}),
              x = 'QRP',
              hue = 'Gender')

plt.show()
```

# Direct effect of gender¶

Prior for the variation between individuals a bit wider, as we saw way more variation there than between fields or levels of seniority in the pilot.

In [13]:

```
with pm.Model(coords=coords) as gender_hyp1:

    # data

    G = pm.MutableData("G", g_idx)
    F = pm.MutableData("F", f_idx)
    S = pm.MutableData("S", s_idx)
    Q = pm.MutableData("Q", q_idx)
    I = pm.MutableData("I", i_idx)

    # fixed hyperpriors for field, seniority and participant
    
    s_ind = pm.Uniform('s_ind', 0,3)
    s_field = pm.Uniform('s_field', 0,2)
    s_seniority = pm.Uniform('s_seniority',0,2)
    
    # prior for the cutpoints, one set of cutpoints per question
    
    cutpoints = pm.Normal('cutpoints',
                           mu=[0,1,2,3,4,5],
                           sigma=1,
                           transform=pm.distributions.transforms.univariate_ordered,
                           dims = ('q_n','c_n')
                           )

    # variable priors for the demographic predictors
    # non-centered to make sampling easier
    
    gender = pm.Normal("gender", 0.0, 1, dims = 'g_n')
    
    z_field = pm.Normal("z_field", 0.0, 1, dims = 'f_n')
    field = pm.Deterministic("field", z_field * s_field, dims = 'f_n')
    
    z_seniority = pm.Normal("z_seniority", 0.0, 1.0, dims = 's_n')
    seniority = pm.Deterministic("seniority", z_seniority * s_seniority, dims = 's_n')
    
    z_ind = pm.Normal("z_ind", 0.0, 1, dims = 'i_n')
    ind = pm.Deterministic("ind", z_ind * s_ind, dims = 'i_n')

    phi =  gender[G] +  ind[I] + field[F] + seniority[S] 
    
    # again, don't compute and store the probabilities for each cutpoint for each category

    y = pm.OrderedLogistic("y", phi, cutpoints[Q], observed=df.score,compute_p = False)
    
    pr = pm.sample_prior_predictive()
```

```
Sampling: [cutpoints, gender, s_field, s_ind, s_seniority, y, z_field, z_ind, z_seniority]
```

In [14]:

```
# plot the priors

variables = ['cutpoints','gender','s_field','s_ind','s_seniority','ind','field','seniority']
fig, axs = plt.subplots(2,4,figsize = (15,7))

for ax, var in zip(axs.flat, variables):
    if len(pr.prior[var].shape) > 2:
        az.plot_posterior(pr.prior[var][:,:,0], ax=ax)
    else:
        az.plot_posterior(pr.prior[var][:,:], ax=ax)
```

In [ ]:

```
# sample from the posterior
# 10k, 4 chains
# high target accept to avoid divergences

with gender_hyp1:
    trace = pm.sample(10000,
                      tune = 2000,
                      return_inferencedata = True,
                      idata_kwargs={"log_likelihood": True},
                      random_seed = SEED,
                      target_accept = 0.99)
```

```
Auto-assigning NUTS sampler...
Initializing NUTS using jitter+adapt_diag...
Multiprocess sampling (4 chains in 4 jobs)
NUTS: [s_ind, s_field, s_seniority, s_cutpoints, cutpoints, gender, z_field, z_seniority, z_ind]
```

77.91% [37399/48000 2:29:47<42:27 Sampling 4 chains, 0 divergences]

In [15]:

```
# save the trace for later use

# trace.to_netcdf('...')

# load

trace = az.from_netcdf('...')
```

In [16]:

```
# dont print cutpoints and individuals cause too many

az.summary(trace, var_names = ['gender','field','seniority', 's_ind','s_field', 's_seniority'])
```

Out[16]:

|  | mean | sd | hdi\_3% | hdi\_97% | mcse\_mean | mcse\_sd | ess\_bulk | ess\_tail | r\_hat |
| --- | --- | --- | --- | --- | --- | --- | --- | --- | --- |
| gender[female] | -0.614 | 0.242 | -1.055 | -0.147 | 0.003 | 0.002 | 6469.0 | 8807.0 | 1.0 |
| gender[male] | -0.644 | 0.237 | -1.074 | -0.186 | 0.003 | 0.002 | 6813.0 | 8800.0 | 1.0 |
| field[Arts & Hum] | -0.327 | 0.232 | -0.767 | 0.085 | 0.003 | 0.002 | 8377.0 | 10183.0 | 1.0 |
| field[Life & Biomed] | 0.306 | 0.208 | -0.061 | 0.722 | 0.002 | 0.002 | 9124.0 | 9627.0 | 1.0 |
| field[Natural Science] | -0.092 | 0.219 | -0.520 | 0.306 | 0.003 | 0.002 | 9002.0 | 9354.0 | 1.0 |
| field[Social Science] | -0.054 | 0.219 | -0.474 | 0.349 | 0.003 | 0.002 | 8659.0 | 9622.0 | 1.0 |
| field[Tech & Engineering] | -0.067 | 0.226 | -0.507 | 0.349 | 0.002 | 0.002 | 9655.0 | 10058.0 | 1.0 |
| seniority[0-10] | -0.010 | 0.117 | -0.247 | 0.206 | 0.001 | 0.001 | 18738.0 | 17561.0 | 1.0 |
| seniority[11-20] | 0.065 | 0.109 | -0.101 | 0.294 | 0.001 | 0.001 | 11148.0 | 16218.0 | 1.0 |
| seniority[21-30] | -0.038 | 0.107 | -0.244 | 0.146 | 0.001 | 0.001 | 14247.0 | 15619.0 | 1.0 |
| seniority[31-40] | -0.034 | 0.110 | -0.255 | 0.158 | 0.001 | 0.001 | 14269.0 | 15397.0 | 1.0 |
| seniority[>40] | -0.022 | 0.115 | -0.253 | 0.189 | 0.001 | 0.001 | 17558.0 | 15961.0 | 1.0 |
| s\_ind | 1.209 | 0.048 | 1.119 | 1.300 | 0.000 | 0.000 | 10968.0 | 19889.0 | 1.0 |
| s\_field | 0.387 | 0.241 | 0.094 | 0.824 | 0.003 | 0.002 | 7303.0 | 8633.0 | 1.0 |
| s\_seniority | 0.128 | 0.131 | 0.000 | 0.328 | 0.002 | 0.001 | 6296.0 | 13942.0 | 1.0 |

In [18]:

```
# cutpoints

pd.set_option('display.max_rows', 850)


az.summary(trace, var_names = ['cutpoints'])
```

Out[18]:

|  | mean | sd | hdi\_3% | hdi\_97% | mcse\_mean | mcse\_sd | ess\_bulk | ess\_tail | r\_hat |
| --- | --- | --- | --- | --- | --- | --- | --- | --- | --- |
| cutpoints[A10, cutpoint\_1] | -0.423 | 0.145 | -0.702 | -0.159 | 0.002 | 0.001 | 7411.0 | 16380.0 | 1.0 |
| cutpoints[A10, cutpoint\_2] | 1.178 | 0.156 | 0.885 | 1.471 | 0.002 | 0.001 | 9566.0 | 19556.0 | 1.0 |
| cutpoints[A10, cutpoint\_3] | 1.985 | 0.175 | 1.666 | 2.324 | 0.002 | 0.001 | 12266.0 | 21868.0 | 1.0 |
| cutpoints[A10, cutpoint\_4] | 3.019 | 0.219 | 2.607 | 3.429 | 0.002 | 0.001 | 19651.0 | 28256.0 | 1.0 |
| cutpoints[A10, cutpoint\_5] | 4.750 | 0.362 | 4.101 | 5.452 | 0.002 | 0.001 | 38469.0 | 31480.0 | 1.0 |
| cutpoints[A10, cutpoint\_6] | 7.068 | 0.638 | 5.888 | 8.272 | 0.002 | 0.002 | 70913.0 | 30884.0 | 1.0 |
| cutpoints[A11, cutpoint\_1] | -0.630 | 0.146 | -0.899 | -0.347 | 0.002 | 0.001 | 7212.0 | 16222.0 | 1.0 |
| cutpoints[A11, cutpoint\_2] | 0.948 | 0.154 | 0.670 | 1.250 | 0.002 | 0.001 | 9013.0 | 18856.0 | 1.0 |
| cutpoints[A11, cutpoint\_3] | 1.724 | 0.169 | 1.409 | 2.045 | 0.002 | 0.001 | 11591.0 | 21715.0 | 1.0 |
| cutpoints[A11, cutpoint\_4] | 2.545 | 0.194 | 2.174 | 2.904 | 0.002 | 0.001 | 15963.0 | 25976.0 | 1.0 |
| cutpoints[A11, cutpoint\_5] | 3.770 | 0.263 | 3.267 | 4.250 | 0.002 | 0.001 | 26521.0 | 31527.0 | 1.0 |
| cutpoints[A11, cutpoint\_6] | 5.036 | 0.382 | 4.317 | 5.751 | 0.002 | 0.001 | 42895.0 | 31172.0 | 1.0 |
| cutpoints[A14, cutpoint\_1] | 0.234 | 0.147 | -0.034 | 0.518 | 0.002 | 0.001 | 7708.0 | 16618.0 | 1.0 |
| cutpoints[A14, cutpoint\_2] | 1.425 | 0.161 | 1.121 | 1.724 | 0.002 | 0.001 | 9789.0 | 19519.0 | 1.0 |
| cutpoints[A14, cutpoint\_3] | 1.991 | 0.175 | 1.659 | 2.313 | 0.002 | 0.001 | 11892.0 | 21963.0 | 1.0 |
| cutpoints[A14, cutpoint\_4] | 2.696 | 0.201 | 2.324 | 3.076 | 0.002 | 0.001 | 16095.0 | 26208.0 | 1.0 |
| cutpoints[A14, cutpoint\_5] | 3.845 | 0.269 | 3.337 | 4.346 | 0.002 | 0.001 | 28468.0 | 26726.0 | 1.0 |
| cutpoints[A14, cutpoint\_6] | 5.048 | 0.384 | 4.317 | 5.759 | 0.002 | 0.001 | 43407.0 | 28741.0 | 1.0 |
| cutpoints[A15, cutpoint\_1] | -0.154 | 0.146 | -0.434 | 0.118 | 0.002 | 0.001 | 7410.0 | 15591.0 | 1.0 |
| cutpoints[A15, cutpoint\_2] | 0.929 | 0.154 | 0.640 | 1.216 | 0.002 | 0.001 | 8607.0 | 17759.0 | 1.0 |
| cutpoints[A15, cutpoint\_3] | 1.615 | 0.166 | 1.311 | 1.936 | 0.002 | 0.001 | 10486.0 | 20498.0 | 1.0 |
| cutpoints[A15, cutpoint\_4] | 2.464 | 0.193 | 2.101 | 2.825 | 0.002 | 0.001 | 14436.0 | 25028.0 | 1.0 |
| cutpoints[A15, cutpoint\_5] | 3.585 | 0.254 | 3.116 | 4.075 | 0.002 | 0.001 | 22832.0 | 28201.0 | 1.0 |
| cutpoints[A15, cutpoint\_6] | 5.266 | 0.421 | 4.482 | 6.053 | 0.002 | 0.001 | 47949.0 | 30821.0 | 1.0 |
| cutpoints[A2, cutpoint\_1] | -1.959 | 0.158 | -2.250 | -1.657 | 0.002 | 0.001 | 7710.0 | 15758.0 | 1.0 |
| cutpoints[A2, cutpoint\_2] | -0.075 | 0.145 | -0.344 | 0.204 | 0.002 | 0.001 | 7972.0 | 16446.0 | 1.0 |
| cutpoints[A2, cutpoint\_3] | 0.785 | 0.150 | 0.502 | 1.066 | 0.002 | 0.001 | 8846.0 | 18545.0 | 1.0 |
| cutpoints[A2, cutpoint\_4] | 1.589 | 0.163 | 1.292 | 1.901 | 0.002 | 0.001 | 10597.0 | 20781.0 | 1.0 |
| cutpoints[A2, cutpoint\_5] | 2.825 | 0.204 | 2.444 | 3.211 | 0.002 | 0.001 | 16709.0 | 25939.0 | 1.0 |
| cutpoints[A2, cutpoint\_6] | 4.152 | 0.297 | 3.605 | 4.722 | 0.002 | 0.001 | 27805.0 | 27295.0 | 1.0 |
| cutpoints[A3, cutpoint\_1] | 0.228 | 0.147 | -0.043 | 0.511 | 0.002 | 0.001 | 7786.0 | 16870.0 | 1.0 |
| cutpoints[A3, cutpoint\_2] | 1.165 | 0.156 | 0.870 | 1.453 | 0.002 | 0.001 | 9202.0 | 17797.0 | 1.0 |
| cutpoints[A3, cutpoint\_3] | 1.747 | 0.168 | 1.419 | 2.049 | 0.002 | 0.001 | 11128.0 | 21096.0 | 1.0 |
| cutpoints[A3, cutpoint\_4] | 2.435 | 0.190 | 2.074 | 2.789 | 0.002 | 0.001 | 14399.0 | 24571.0 | 1.0 |
| cutpoints[A3, cutpoint\_5] | 3.564 | 0.252 | 3.100 | 4.044 | 0.002 | 0.001 | 23690.0 | 28392.0 | 1.0 |
| cutpoints[A3, cutpoint\_6] | 5.588 | 0.471 | 4.710 | 6.470 | 0.002 | 0.002 | 41253.0 | 30491.0 | 1.0 |
| cutpoints[A5, cutpoint\_1] | -0.524 | 0.146 | -0.809 | -0.262 | 0.002 | 0.001 | 7146.0 | 14780.0 | 1.0 |
| cutpoints[A5, cutpoint\_2] | 0.856 | 0.152 | 0.576 | 1.146 | 0.002 | 0.001 | 8641.0 | 17499.0 | 1.0 |
| cutpoints[A5, cutpoint\_3] | 1.665 | 0.167 | 1.350 | 1.977 | 0.002 | 0.001 | 10594.0 | 20861.0 | 1.0 |
| cutpoints[A5, cutpoint\_4] | 2.272 | 0.184 | 1.932 | 2.620 | 0.002 | 0.001 | 13658.0 | 22772.0 | 1.0 |
| cutpoints[A5, cutpoint\_5] | 2.968 | 0.211 | 2.568 | 3.361 | 0.002 | 0.001 | 17542.0 | 24268.0 | 1.0 |
| cutpoints[A5, cutpoint\_6] | 3.991 | 0.278 | 3.490 | 4.532 | 0.002 | 0.001 | 27909.0 | 28443.0 | 1.0 |
| cutpoints[A6, cutpoint\_1] | 0.352 | 0.147 | 0.080 | 0.632 | 0.002 | 0.001 | 7838.0 | 16943.0 | 1.0 |
| cutpoints[A6, cutpoint\_2] | 1.643 | 0.165 | 1.331 | 1.951 | 0.002 | 0.001 | 10543.0 | 20508.0 | 1.0 |
| cutpoints[A6, cutpoint\_3] | 2.362 | 0.184 | 2.020 | 2.711 | 0.002 | 0.001 | 14322.0 | 24024.0 | 1.0 |
| cutpoints[A6, cutpoint\_4] | 3.325 | 0.231 | 2.904 | 3.773 | 0.002 | 0.001 | 23069.0 | 28143.0 | 1.0 |
| cutpoints[A6, cutpoint\_5] | 4.316 | 0.304 | 3.753 | 4.896 | 0.002 | 0.001 | 36606.0 | 29130.0 | 1.0 |
| cutpoints[A6, cutpoint\_6] | 5.120 | 0.380 | 4.437 | 5.858 | 0.002 | 0.001 | 47695.0 | 29922.0 | 1.0 |
| cutpoints[A8, cutpoint\_1] | -0.601 | 0.145 | -0.870 | -0.328 | 0.002 | 0.001 | 7280.0 | 15787.0 | 1.0 |
| cutpoints[A8, cutpoint\_2] | 0.758 | 0.150 | 0.478 | 1.040 | 0.002 | 0.001 | 8477.0 | 19266.0 | 1.0 |
| cutpoints[A8, cutpoint\_3] | 1.460 | 0.160 | 1.154 | 1.754 | 0.002 | 0.001 | 10083.0 | 20655.0 | 1.0 |
| cutpoints[A8, cutpoint\_4] | 1.983 | 0.173 | 1.648 | 2.295 | 0.002 | 0.001 | 11815.0 | 21693.0 | 1.0 |
| cutpoints[A8, cutpoint\_5] | 2.911 | 0.208 | 2.519 | 3.303 | 0.002 | 0.001 | 16145.0 | 24693.0 | 1.0 |
| cutpoints[A8, cutpoint\_6] | 4.209 | 0.297 | 3.664 | 4.777 | 0.002 | 0.001 | 29677.0 | 30720.0 | 1.0 |
| cutpoints[A9, cutpoint\_1] | -0.917 | 0.147 | -1.192 | -0.640 | 0.002 | 0.001 | 7492.0 | 15644.0 | 1.0 |
| cutpoints[A9, cutpoint\_2] | 0.505 | 0.148 | 0.227 | 0.783 | 0.002 | 0.001 | 8187.0 | 17550.0 | 1.0 |
| cutpoints[A9, cutpoint\_3] | 1.276 | 0.157 | 0.982 | 1.568 | 0.002 | 0.001 | 9284.0 | 18721.0 | 1.0 |
| cutpoints[A9, cutpoint\_4] | 1.809 | 0.167 | 1.499 | 2.128 | 0.002 | 0.001 | 10982.0 | 21550.0 | 1.0 |
| cutpoints[A9, cutpoint\_5] | 2.479 | 0.187 | 2.129 | 2.832 | 0.002 | 0.001 | 13483.0 | 24256.0 | 1.0 |
| cutpoints[A9, cutpoint\_6] | 3.569 | 0.244 | 3.123 | 4.039 | 0.002 | 0.001 | 22140.0 | 26550.0 | 1.0 |
| cutpoints[P4, cutpoint\_1] | -1.358 | 0.155 | -1.648 | -1.065 | 0.002 | 0.001 | 7597.0 | 15548.0 | 1.0 |
| cutpoints[P4, cutpoint\_2] | 0.728 | 0.155 | 0.441 | 1.025 | 0.002 | 0.001 | 8811.0 | 18401.0 | 1.0 |
| cutpoints[P4, cutpoint\_3] | 1.759 | 0.175 | 1.434 | 2.090 | 0.002 | 0.001 | 11532.0 | 22305.0 | 1.0 |
| cutpoints[P4, cutpoint\_4] | 2.804 | 0.216 | 2.402 | 3.214 | 0.002 | 0.001 | 17693.0 | 26526.0 | 1.0 |
| cutpoints[P4, cutpoint\_5] | 4.068 | 0.305 | 3.509 | 4.658 | 0.002 | 0.001 | 35594.0 | 31463.0 | 1.0 |
| cutpoints[P4, cutpoint\_6] | 5.183 | 0.414 | 4.420 | 5.967 | 0.002 | 0.001 | 47366.0 | 30244.0 | 1.0 |
| cutpoints[R4, cutpoint\_1] | -1.754 | 0.147 | -2.029 | -1.477 | 0.002 | 0.001 | 7309.0 | 15158.0 | 1.0 |
| cutpoints[R4, cutpoint\_2] | 0.433 | 0.142 | 0.168 | 0.701 | 0.002 | 0.001 | 7462.0 | 16794.0 | 1.0 |
| cutpoints[R4, cutpoint\_3] | 1.254 | 0.150 | 0.960 | 1.527 | 0.002 | 0.001 | 8419.0 | 18264.0 | 1.0 |
| cutpoints[R4, cutpoint\_4] | 2.325 | 0.175 | 1.999 | 2.653 | 0.002 | 0.001 | 11432.0 | 22209.0 | 1.0 |
| cutpoints[R4, cutpoint\_5] | 3.730 | 0.245 | 3.278 | 4.195 | 0.002 | 0.001 | 20967.0 | 28484.0 | 1.0 |
| cutpoints[R4, cutpoint\_6] | 5.739 | 0.465 | 4.896 | 6.639 | 0.002 | 0.002 | 45899.0 | 28707.0 | 1.0 |
| cutpoints[R5, cutpoint\_1] | 0.897 | 0.146 | 0.624 | 1.169 | 0.002 | 0.001 | 7784.0 | 16312.0 | 1.0 |
| cutpoints[R5, cutpoint\_2] | 2.134 | 0.170 | 1.815 | 2.454 | 0.002 | 0.001 | 11530.0 | 21303.0 | 1.0 |
| cutpoints[R5, cutpoint\_3] | 2.851 | 0.198 | 2.473 | 3.216 | 0.002 | 0.001 | 15960.0 | 22542.0 | 1.0 |
| cutpoints[R5, cutpoint\_4] | 3.555 | 0.238 | 3.102 | 3.995 | 0.002 | 0.001 | 22920.0 | 26418.0 | 1.0 |
| cutpoints[R5, cutpoint\_5] | 4.612 | 0.316 | 4.023 | 5.202 | 0.002 | 0.001 | 36188.0 | 29506.0 | 1.0 |
| cutpoints[R5, cutpoint\_6] | 5.694 | 0.424 | 4.928 | 6.512 | 0.002 | 0.001 | 50740.0 | 30285.0 | 1.0 |
| cutpoints[R6, cutpoint\_1] | -0.466 | 0.141 | -0.731 | -0.206 | 0.002 | 0.001 | 6898.0 | 14137.0 | 1.0 |
| cutpoints[R6, cutpoint\_2] | 1.429 | 0.154 | 1.141 | 1.721 | 0.002 | 0.001 | 9095.0 | 19081.0 | 1.0 |
| cutpoints[R6, cutpoint\_3] | 2.472 | 0.182 | 2.132 | 2.817 | 0.002 | 0.001 | 12641.0 | 22526.0 | 1.0 |
| cutpoints[R6, cutpoint\_4] | 3.523 | 0.233 | 3.093 | 3.966 | 0.002 | 0.001 | 21077.0 | 29625.0 | 1.0 |
| cutpoints[R6, cutpoint\_5] | 4.889 | 0.349 | 4.233 | 5.541 | 0.002 | 0.001 | 39426.0 | 30096.0 | 1.0 |
| cutpoints[R6, cutpoint\_6] | 6.833 | 0.586 | 5.768 | 7.961 | 0.002 | 0.002 | 59910.0 | 28077.0 | 1.0 |
| cutpoints[R7, cutpoint\_1] | 0.295 | 0.142 | 0.027 | 0.559 | 0.002 | 0.001 | 7154.0 | 15111.0 | 1.0 |
| cutpoints[R7, cutpoint\_2] | 1.978 | 0.166 | 1.666 | 2.289 | 0.002 | 0.001 | 11301.0 | 21895.0 | 1.0 |
| cutpoints[R7, cutpoint\_3] | 2.893 | 0.200 | 2.510 | 3.260 | 0.002 | 0.001 | 16831.0 | 27227.0 | 1.0 |
| cutpoints[R7, cutpoint\_4] | 3.520 | 0.235 | 3.085 | 3.969 | 0.002 | 0.001 | 23404.0 | 27266.0 | 1.0 |
| cutpoints[R7, cutpoint\_5] | 5.099 | 0.374 | 4.397 | 5.794 | 0.002 | 0.001 | 45558.0 | 31496.0 | 1.0 |
| cutpoints[R7, cutpoint\_6] | 7.236 | 0.626 | 6.075 | 8.406 | 0.003 | 0.002 | 58555.0 | 29395.0 | 1.0 |
| cutpoints[R8, cutpoint\_1] | 1.019 | 0.148 | 0.745 | 1.300 | 0.002 | 0.001 | 7821.0 | 17579.0 | 1.0 |
| cutpoints[R8, cutpoint\_2] | 2.935 | 0.200 | 2.557 | 3.311 | 0.002 | 0.001 | 15176.0 | 25289.0 | 1.0 |
| cutpoints[R8, cutpoint\_3] | 3.892 | 0.257 | 3.420 | 4.386 | 0.002 | 0.001 | 26725.0 | 27415.0 | 1.0 |
| cutpoints[R8, cutpoint\_4] | 4.976 | 0.345 | 4.345 | 5.635 | 0.002 | 0.001 | 41384.0 | 31850.0 | 1.0 |
| cutpoints[R8, cutpoint\_5] | 6.252 | 0.471 | 5.372 | 7.134 | 0.002 | 0.001 | 59672.0 | 29773.0 | 1.0 |
| cutpoints[R8, cutpoint\_6] | 8.096 | 0.674 | 6.859 | 9.380 | 0.003 | 0.002 | 69256.0 | 29770.0 | 1.0 |

In [19]:

```
# individuals

az.summary(trace, var_names = ['ind'])
```

Out[19]:

|  | mean | sd | hdi\_3% | hdi\_97% | mcse\_mean | mcse\_sd | ess\_bulk | ess\_tail | r\_hat |
| --- | --- | --- | --- | --- | --- | --- | --- | --- | --- |
| ind[0] | 0.994 | 0.450 | 0.154 | 1.854 | 0.002 | 0.002 | 36593.0 | 28005.0 | 1.0 |
| ind[1] | 1.284 | 0.487 | 0.368 | 2.201 | 0.002 | 0.002 | 39489.0 | 28865.0 | 1.0 |
| ind[2] | 0.346 | 0.475 | -0.550 | 1.233 | 0.002 | 0.002 | 46468.0 | 30518.0 | 1.0 |
| ind[3] | -1.426 | 0.874 | -3.099 | 0.187 | 0.004 | 0.003 | 53183.0 | 29705.0 | 1.0 |
| ind[4] | 0.485 | 0.535 | -0.523 | 1.489 | 0.003 | 0.002 | 45389.0 | 27952.0 | 1.0 |
| ind[5] | 1.666 | 0.489 | 0.732 | 2.573 | 0.003 | 0.002 | 37255.0 | 29816.0 | 1.0 |
| ind[6] | 1.539 | 0.408 | 0.752 | 2.280 | 0.002 | 0.002 | 36496.0 | 27296.0 | 1.0 |
| ind[8] | -1.441 | 0.873 | -3.076 | 0.201 | 0.004 | 0.003 | 51994.0 | 27629.0 | 1.0 |
| ind[9] | 0.411 | 0.509 | -0.555 | 1.361 | 0.003 | 0.002 | 37776.0 | 29669.0 | 1.0 |
| ind[10] | 0.678 | 0.473 | -0.217 | 1.561 | 0.002 | 0.002 | 44871.0 | 27460.0 | 1.0 |
| ind[11] | -0.411 | 0.734 | -1.797 | 0.938 | 0.003 | 0.003 | 50620.0 | 28600.0 | 1.0 |
| ind[12] | 0.895 | 0.435 | 0.084 | 1.719 | 0.002 | 0.002 | 35876.0 | 26761.0 | 1.0 |
| ind[13] | -0.709 | 0.565 | -1.762 | 0.359 | 0.003 | 0.002 | 40467.0 | 28377.0 | 1.0 |
| ind[14] | -0.163 | 0.488 | -1.072 | 0.755 | 0.002 | 0.002 | 40208.0 | 27479.0 | 1.0 |
| ind[15] | 0.593 | 0.495 | -0.354 | 1.509 | 0.002 | 0.002 | 39875.0 | 29433.0 | 1.0 |
| ind[16] | -0.415 | 0.505 | -1.375 | 0.521 | 0.002 | 0.002 | 42162.0 | 27668.0 | 1.0 |
| ind[17] | 0.151 | 0.985 | -1.692 | 2.024 | 0.004 | 0.005 | 58819.0 | 30221.0 | 1.0 |
| ind[18] | -0.659 | 0.593 | -1.760 | 0.472 | 0.003 | 0.003 | 46242.0 | 27687.0 | 1.0 |
| ind[19] | -0.173 | 0.517 | -1.151 | 0.787 | 0.002 | 0.003 | 44652.0 | 29020.0 | 1.0 |
| ind[20] | -0.012 | 0.465 | -0.878 | 0.863 | 0.002 | 0.003 | 45377.0 | 26994.0 | 1.0 |
| ind[21] | -0.098 | 0.464 | -0.969 | 0.774 | 0.002 | 0.002 | 43441.0 | 28816.0 | 1.0 |
| ind[22] | 1.275 | 0.452 | 0.425 | 2.118 | 0.003 | 0.002 | 31750.0 | 27957.0 | 1.0 |
| ind[24] | 0.905 | 0.424 | 0.107 | 1.703 | 0.002 | 0.002 | 36235.0 | 28780.0 | 1.0 |
| ind[25] | -1.014 | 0.578 | -2.108 | 0.062 | 0.003 | 0.002 | 48106.0 | 28467.0 | 1.0 |
| ind[26] | 0.751 | 0.475 | -0.138 | 1.648 | 0.002 | 0.002 | 43160.0 | 27127.0 | 1.0 |
| ind[27] | -0.774 | 0.537 | -1.749 | 0.265 | 0.003 | 0.002 | 44051.0 | 28573.0 | 1.0 |
| ind[28] | 0.279 | 0.434 | -0.561 | 1.072 | 0.002 | 0.002 | 38334.0 | 27237.0 | 1.0 |
| ind[29] | 0.982 | 0.456 | 0.130 | 1.842 | 0.002 | 0.002 | 37415.0 | 28675.0 | 1.0 |
| ind[30] | 0.222 | 0.455 | -0.627 | 1.079 | 0.002 | 0.002 | 35286.0 | 29037.0 | 1.0 |
| ind[31] | -0.755 | 0.554 | -1.837 | 0.237 | 0.003 | 0.002 | 47368.0 | 28028.0 | 1.0 |
| ind[32] | -0.671 | 0.518 | -1.624 | 0.314 | 0.003 | 0.002 | 42862.0 | 28492.0 | 1.0 |
| ind[33] | 0.364 | 0.431 | -0.453 | 1.161 | 0.002 | 0.002 | 38852.0 | 29237.0 | 1.0 |
| ind[34] | 1.400 | 0.429 | 0.581 | 2.194 | 0.002 | 0.001 | 43637.0 | 28910.0 | 1.0 |
| ind[35] | -0.298 | 0.522 | -1.287 | 0.660 | 0.003 | 0.002 | 39939.0 | 29173.0 | 1.0 |
| ind[36] | 1.838 | 0.480 | 0.931 | 2.742 | 0.002 | 0.002 | 45107.0 | 28239.0 | 1.0 |
| ind[37] | -0.315 | 0.491 | -1.223 | 0.633 | 0.002 | 0.002 | 39993.0 | 29045.0 | 1.0 |
| ind[38] | 0.124 | 0.709 | -1.218 | 1.458 | 0.003 | 0.004 | 42896.0 | 28674.0 | 1.0 |
| ind[39] | -0.259 | 0.462 | -1.131 | 0.603 | 0.002 | 0.002 | 34477.0 | 28192.0 | 1.0 |
| ind[40] | -0.654 | 0.547 | -1.703 | 0.348 | 0.002 | 0.002 | 49004.0 | 28758.0 | 1.0 |
| ind[41] | 0.758 | 0.440 | -0.090 | 1.567 | 0.002 | 0.002 | 33565.0 | 27185.0 | 1.0 |
| ind[42] | 0.541 | 0.491 | -0.386 | 1.462 | 0.002 | 0.002 | 38923.0 | 28306.0 | 1.0 |
| ind[43] | 1.047 | 0.464 | 0.152 | 1.896 | 0.002 | 0.002 | 39539.0 | 27802.0 | 1.0 |
| ind[44] | 0.361 | 0.482 | -0.543 | 1.263 | 0.002 | 0.002 | 40062.0 | 27901.0 | 1.0 |
| ind[45] | 0.095 | 0.980 | -1.751 | 1.939 | 0.004 | 0.005 | 70972.0 | 29788.0 | 1.0 |
| ind[46] | -0.950 | 0.789 | -2.444 | 0.522 | 0.003 | 0.003 | 53177.0 | 28130.0 | 1.0 |
| ind[47] | -0.604 | 0.556 | -1.638 | 0.450 | 0.003 | 0.002 | 42683.0 | 27873.0 | 1.0 |
| ind[48] | -0.289 | 0.466 | -1.168 | 0.579 | 0.003 | 0.002 | 34510.0 | 28344.0 | 1.0 |
| ind[50] | -0.340 | 0.497 | -1.293 | 0.577 | 0.003 | 0.002 | 39806.0 | 26551.0 | 1.0 |
| ind[51] | 2.969 | 0.481 | 2.083 | 3.884 | 0.002 | 0.002 | 39779.0 | 28403.0 | 1.0 |
| ind[52] | 0.640 | 0.436 | -0.190 | 1.451 | 0.002 | 0.002 | 37773.0 | 28705.0 | 1.0 |
| ind[53] | 0.499 | 0.472 | -0.408 | 1.364 | 0.002 | 0.002 | 44025.0 | 28717.0 | 1.0 |
| ind[54] | 0.094 | 0.482 | -0.814 | 0.986 | 0.002 | 0.003 | 43711.0 | 28620.0 | 1.0 |
| ind[55] | -0.946 | 0.580 | -2.015 | 0.159 | 0.003 | 0.002 | 39916.0 | 29176.0 | 1.0 |
| ind[56] | -1.542 | 0.867 | -3.154 | 0.093 | 0.004 | 0.003 | 54369.0 | 26844.0 | 1.0 |
| ind[57] | -0.338 | 0.555 | -1.400 | 0.678 | 0.003 | 0.003 | 45993.0 | 29689.0 | 1.0 |
| ind[58] | 0.038 | 0.503 | -0.900 | 0.985 | 0.003 | 0.003 | 36869.0 | 28840.0 | 1.0 |
| ind[59] | 0.510 | 0.472 | -0.361 | 1.410 | 0.002 | 0.002 | 40766.0 | 29231.0 | 1.0 |
| ind[60] | 1.816 | 0.459 | 0.946 | 2.674 | 0.003 | 0.002 | 31914.0 | 27356.0 | 1.0 |
| ind[61] | 2.578 | 0.413 | 1.801 | 3.359 | 0.002 | 0.001 | 41125.0 | 29297.0 | 1.0 |
| ind[62] | 0.107 | 0.693 | -1.219 | 1.387 | 0.003 | 0.004 | 52985.0 | 27893.0 | 1.0 |
| ind[63] | 2.596 | 0.452 | 1.724 | 3.429 | 0.002 | 0.002 | 37942.0 | 27971.0 | 1.0 |
| ind[64] | 0.118 | 0.557 | -0.944 | 1.148 | 0.002 | 0.003 | 51984.0 | 27193.0 | 1.0 |
| ind[65] | 2.019 | 0.432 | 1.220 | 2.843 | 0.002 | 0.002 | 37402.0 | 28502.0 | 1.0 |
| ind[66] | 0.137 | 0.463 | -0.731 | 1.000 | 0.002 | 0.002 | 38927.0 | 27662.0 | 1.0 |
| ind[67] | -0.609 | 0.481 | -1.517 | 0.288 | 0.002 | 0.002 | 45723.0 | 29637.0 | 1.0 |
| ind[68] | -0.058 | 0.464 | -0.910 | 0.830 | 0.002 | 0.002 | 36475.0 | 29969.0 | 1.0 |
| ind[69] | -0.601 | 0.555 | -1.652 | 0.422 | 0.003 | 0.002 | 39200.0 | 29107.0 | 1.0 |
| ind[70] | -0.380 | 0.506 | -1.303 | 0.595 | 0.003 | 0.002 | 38383.0 | 26836.0 | 1.0 |
| ind[71] | 1.100 | 0.449 | 0.262 | 1.948 | 0.003 | 0.002 | 31852.0 | 27575.0 | 1.0 |
| ind[72] | -0.906 | 0.536 | -1.896 | 0.109 | 0.002 | 0.002 | 48233.0 | 29685.0 | 1.0 |
| ind[73] | -0.332 | 0.480 | -1.242 | 0.551 | 0.002 | 0.002 | 47788.0 | 28751.0 | 1.0 |
| ind[74] | 0.139 | 0.464 | -0.747 | 0.986 | 0.002 | 0.002 | 38531.0 | 26985.0 | 1.0 |
| ind[75] | -0.731 | 0.485 | -1.663 | 0.166 | 0.002 | 0.002 | 39178.0 | 29368.0 | 1.0 |
| ind[76] | -0.947 | 0.560 | -1.977 | 0.142 | 0.003 | 0.002 | 46149.0 | 27958.0 | 1.0 |
| ind[77] | -1.419 | 0.883 | -3.054 | 0.260 | 0.004 | 0.003 | 50268.0 | 28339.0 | 1.0 |
| ind[78] | 0.302 | 0.420 | -0.486 | 1.086 | 0.002 | 0.002 | 33431.0 | 26954.0 | 1.0 |
| ind[79] | 1.439 | 0.472 | 0.550 | 2.315 | 0.002 | 0.002 | 41505.0 | 28798.0 | 1.0 |
| ind[80] | 0.150 | 0.441 | -0.681 | 0.979 | 0.002 | 0.002 | 38566.0 | 28604.0 | 1.0 |
| ind[81] | -0.563 | 0.501 | -1.495 | 0.391 | 0.002 | 0.002 | 47579.0 | 27367.0 | 1.0 |
| ind[82] | 0.346 | 0.456 | -0.528 | 1.192 | 0.002 | 0.002 | 40702.0 | 28132.0 | 1.0 |
| ind[83] | -0.063 | 0.499 | -0.989 | 0.879 | 0.002 | 0.003 | 43590.0 | 27026.0 | 1.0 |
| ind[84] | -1.677 | 0.614 | -2.830 | -0.527 | 0.003 | 0.002 | 47630.0 | 27156.0 | 1.0 |
| ind[85] | 0.853 | 0.432 | 0.054 | 1.669 | 0.002 | 0.002 | 33208.0 | 28510.0 | 1.0 |
| ind[86] | 0.519 | 0.474 | -0.391 | 1.390 | 0.002 | 0.002 | 40188.0 | 28185.0 | 1.0 |
| ind[87] | -0.165 | 0.489 | -1.097 | 0.744 | 0.002 | 0.002 | 40846.0 | 29087.0 | 1.0 |
| ind[88] | -0.275 | 0.473 | -1.170 | 0.609 | 0.002 | 0.002 | 43423.0 | 28178.0 | 1.0 |
| ind[89] | 0.417 | 0.736 | -0.979 | 1.785 | 0.003 | 0.003 | 46533.0 | 29828.0 | 1.0 |
| ind[90] | -0.077 | 0.526 | -1.050 | 0.928 | 0.003 | 0.003 | 39259.0 | 28837.0 | 1.0 |
| ind[91] | -0.231 | 0.475 | -1.131 | 0.653 | 0.002 | 0.002 | 45650.0 | 29150.0 | 1.0 |
| ind[92] | 0.236 | 0.472 | -0.669 | 1.099 | 0.002 | 0.002 | 40804.0 | 28917.0 | 1.0 |
| ind[93] | -1.565 | 0.608 | -2.719 | -0.439 | 0.003 | 0.002 | 52910.0 | 28530.0 | 1.0 |
| ind[94] | -2.276 | 0.744 | -3.685 | -0.919 | 0.004 | 0.003 | 44482.0 | 26441.0 | 1.0 |
| ind[95] | 2.082 | 0.500 | 1.137 | 3.019 | 0.002 | 0.002 | 43418.0 | 29805.0 | 1.0 |
| ind[96] | -0.942 | 0.543 | -1.953 | 0.082 | 0.003 | 0.002 | 40560.0 | 27905.0 | 1.0 |
| ind[97] | 0.698 | 0.466 | -0.180 | 1.578 | 0.003 | 0.002 | 34190.0 | 28138.0 | 1.0 |
| ind[99] | 1.061 | 0.418 | 0.273 | 1.842 | 0.002 | 0.002 | 33154.0 | 29151.0 | 1.0 |
| ind[100] | -0.655 | 0.496 | -1.591 | 0.265 | 0.002 | 0.002 | 41425.0 | 27283.0 | 1.0 |
| ind[101] | -1.471 | 0.614 | -2.607 | -0.307 | 0.003 | 0.002 | 51955.0 | 27165.0 | 1.0 |
| ind[102] | 0.698 | 0.466 | -0.166 | 1.580 | 0.002 | 0.002 | 45271.0 | 28423.0 | 1.0 |
| ind[103] | 1.605 | 0.455 | 0.749 | 2.458 | 0.002 | 0.002 | 38645.0 | 29050.0 | 1.0 |
| ind[104] | 0.091 | 0.490 | -0.834 | 1.006 | 0.003 | 0.002 | 37585.0 | 28579.0 | 1.0 |
| ind[105] | 1.341 | 0.457 | 0.482 | 2.196 | 0.002 | 0.002 | 39370.0 | 27802.0 | 1.0 |
| ind[106] | 1.419 | 0.501 | 0.475 | 2.367 | 0.002 | 0.002 | 41912.0 | 30061.0 | 1.0 |
| ind[107] | -1.068 | 0.579 | -2.152 | 0.026 | 0.003 | 0.002 | 48176.0 | 27990.0 | 1.0 |
| ind[108] | 0.517 | 0.445 | -0.309 | 1.365 | 0.002 | 0.002 | 36897.0 | 27856.0 | 1.0 |
| ind[109] | 0.871 | 0.413 | 0.082 | 1.638 | 0.002 | 0.002 | 39132.0 | 28709.0 | 1.0 |
| ind[110] | 0.187 | 0.436 | -0.634 | 1.008 | 0.002 | 0.002 | 40712.0 | 28941.0 | 1.0 |
| ind[111] | 0.790 | 0.487 | -0.154 | 1.682 | 0.002 | 0.002 | 40449.0 | 29914.0 | 1.0 |
| ind[112] | -0.988 | 0.536 | -2.034 | -0.023 | 0.003 | 0.002 | 41059.0 | 27079.0 | 1.0 |
| ind[113] | 0.269 | 0.515 | -0.695 | 1.240 | 0.003 | 0.002 | 39506.0 | 28139.0 | 1.0 |
| ind[114] | 0.102 | 0.460 | -0.762 | 0.970 | 0.002 | 0.002 | 39394.0 | 28896.0 | 1.0 |
| ind[115] | 0.539 | 0.526 | -0.466 | 1.519 | 0.002 | 0.002 | 45775.0 | 27708.0 | 1.0 |
| ind[116] | 0.724 | 0.471 | -0.155 | 1.622 | 0.003 | 0.002 | 34667.0 | 28668.0 | 1.0 |
| ind[117] | -0.629 | 0.518 | -1.595 | 0.350 | 0.003 | 0.002 | 42695.0 | 28114.0 | 1.0 |
| ind[118] | 1.084 | 0.491 | 0.168 | 2.011 | 0.002 | 0.002 | 47499.0 | 29094.0 | 1.0 |
| ind[119] | -0.041 | 0.511 | -0.998 | 0.922 | 0.002 | 0.003 | 51441.0 | 28361.0 | 1.0 |
| ind[120] | 0.133 | 0.479 | -0.782 | 1.019 | 0.002 | 0.002 | 38676.0 | 28575.0 | 1.0 |
| ind[121] | -1.699 | 0.610 | -2.840 | -0.562 | 0.003 | 0.002 | 48512.0 | 28607.0 | 1.0 |
| ind[122] | -1.738 | 0.678 | -3.009 | -0.477 | 0.003 | 0.002 | 51687.0 | 27341.0 | 1.0 |
| ind[123] | -0.439 | 0.495 | -1.386 | 0.481 | 0.002 | 0.002 | 41160.0 | 26931.0 | 1.0 |
| ind[124] | -1.210 | 0.638 | -2.421 | -0.037 | 0.003 | 0.002 | 45446.0 | 28019.0 | 1.0 |
| ind[125] | 1.644 | 0.420 | 0.852 | 2.426 | 0.002 | 0.002 | 34703.0 | 27762.0 | 1.0 |
| ind[126] | -0.666 | 0.820 | -2.257 | 0.828 | 0.004 | 0.004 | 50392.0 | 27238.0 | 1.0 |
| ind[127] | 0.254 | 0.467 | -0.635 | 1.128 | 0.002 | 0.002 | 39218.0 | 29842.0 | 1.0 |
| ind[128] | 0.314 | 0.631 | -0.872 | 1.503 | 0.003 | 0.003 | 44684.0 | 29551.0 | 1.0 |
| ind[129] | -0.670 | 0.485 | -1.585 | 0.243 | 0.002 | 0.002 | 43038.0 | 28672.0 | 1.0 |
| ind[130] | -0.852 | 0.517 | -1.814 | 0.125 | 0.002 | 0.002 | 47654.0 | 28955.0 | 1.0 |
| ind[131] | 0.711 | 0.426 | -0.104 | 1.505 | 0.002 | 0.002 | 40846.0 | 29783.0 | 1.0 |
| ind[132] | 1.324 | 0.414 | 0.546 | 2.097 | 0.002 | 0.002 | 35860.0 | 28997.0 | 1.0 |
| ind[133] | 1.160 | 0.465 | 0.296 | 2.040 | 0.002 | 0.002 | 38930.0 | 28693.0 | 1.0 |
| ind[134] | -0.098 | 0.689 | -1.454 | 1.143 | 0.003 | 0.004 | 54332.0 | 28354.0 | 1.0 |
| ind[135] | -0.333 | 0.754 | -1.799 | 1.037 | 0.003 | 0.004 | 52169.0 | 29360.0 | 1.0 |
| ind[136] | 0.569 | 0.425 | -0.266 | 1.337 | 0.002 | 0.002 | 40122.0 | 28031.0 | 1.0 |
| ind[137] | -0.223 | 0.514 | -1.189 | 0.734 | 0.002 | 0.003 | 48026.0 | 29374.0 | 1.0 |
| ind[138] | -0.495 | 0.526 | -1.483 | 0.496 | 0.003 | 0.002 | 33839.0 | 27821.0 | 1.0 |
| ind[139] | -1.744 | 0.600 | -2.898 | -0.646 | 0.003 | 0.002 | 46234.0 | 28209.0 | 1.0 |
| ind[140] | -1.092 | 0.533 | -2.067 | -0.061 | 0.002 | 0.002 | 47166.0 | 28751.0 | 1.0 |
| ind[141] | 0.428 | 0.462 | -0.452 | 1.287 | 0.002 | 0.002 | 35987.0 | 28428.0 | 1.0 |
| ind[142] | 0.122 | 0.530 | -0.878 | 1.111 | 0.003 | 0.003 | 39628.0 | 27685.0 | 1.0 |
| ind[143] | -0.441 | 0.518 | -1.392 | 0.551 | 0.003 | 0.002 | 42393.0 | 28424.0 | 1.0 |
| ind[145] | -1.196 | 0.632 | -2.379 | -0.009 | 0.003 | 0.002 | 49086.0 | 27521.0 | 1.0 |
| ind[146] | 0.000 | 1.212 | -2.229 | 2.330 | 0.005 | 0.006 | 56898.0 | 30785.0 | 1.0 |
| ind[148] | 1.990 | 0.499 | 1.064 | 2.933 | 0.002 | 0.002 | 45833.0 | 29649.0 | 1.0 |
| ind[149] | 0.113 | 0.993 | -1.744 | 1.993 | 0.004 | 0.006 | 53100.0 | 28082.0 | 1.0 |
| ind[150] | -0.685 | 0.501 | -1.610 | 0.278 | 0.002 | 0.002 | 44819.0 | 30418.0 | 1.0 |
| ind[151] | 1.689 | 0.450 | 0.846 | 2.542 | 0.002 | 0.002 | 38927.0 | 28449.0 | 1.0 |
| ind[152] | 0.613 | 0.488 | -0.303 | 1.527 | 0.002 | 0.002 | 42978.0 | 29164.0 | 1.0 |
| ind[153] | -0.504 | 0.725 | -1.858 | 0.864 | 0.003 | 0.003 | 53815.0 | 29761.0 | 1.0 |
| ind[154] | 1.165 | 0.431 | 0.352 | 1.975 | 0.002 | 0.002 | 40393.0 | 27219.0 | 1.0 |
| ind[155] | -2.027 | 0.657 | -3.273 | -0.797 | 0.003 | 0.002 | 47850.0 | 28226.0 | 1.0 |
| ind[156] | 0.119 | 0.441 | -0.719 | 0.940 | 0.002 | 0.002 | 40754.0 | 27140.0 | 1.0 |
| ind[157] | -0.398 | 0.496 | -1.338 | 0.522 | 0.003 | 0.002 | 38477.0 | 29471.0 | 1.0 |
| ind[158] | -0.746 | 0.808 | -2.272 | 0.760 | 0.004 | 0.003 | 47377.0 | 28561.0 | 1.0 |
| ind[159] | 0.481 | 0.460 | -0.389 | 1.338 | 0.002 | 0.002 | 47249.0 | 29973.0 | 1.0 |
| ind[160] | -0.359 | 0.590 | -1.450 | 0.768 | 0.003 | 0.003 | 43516.0 | 27835.0 | 1.0 |
| ind[161] | 0.413 | 0.500 | -0.515 | 1.373 | 0.002 | 0.002 | 42000.0 | 27909.0 | 1.0 |
| ind[162] | 1.266 | 0.453 | 0.429 | 2.145 | 0.002 | 0.002 | 42463.0 | 26934.0 | 1.0 |
| ind[163] | -0.770 | 0.674 | -2.053 | 0.470 | 0.003 | 0.003 | 41662.0 | 28924.0 | 1.0 |
| ind[165] | 0.721 | 0.466 | -0.164 | 1.586 | 0.002 | 0.002 | 36528.0 | 29747.0 | 1.0 |
| ind[166] | -0.202 | 0.536 | -1.216 | 0.800 | 0.003 | 0.003 | 41923.0 | 27991.0 | 1.0 |
| ind[167] | 0.021 | 0.673 | -1.247 | 1.272 | 0.003 | 0.004 | 49625.0 | 29318.0 | 1.0 |
| ind[168] | -0.436 | 0.547 | -1.468 | 0.591 | 0.003 | 0.002 | 44478.0 | 28720.0 | 1.0 |
| ind[169] | -0.045 | 0.492 | -1.000 | 0.848 | 0.002 | 0.003 | 43549.0 | 29155.0 | 1.0 |
| ind[170] | 0.112 | 0.523 | -0.880 | 1.095 | 0.003 | 0.003 | 38599.0 | 28881.0 | 1.0 |
| ind[171] | -0.135 | 0.460 | -1.003 | 0.716 | 0.002 | 0.002 | 43370.0 | 30669.0 | 1.0 |
| ind[172] | 0.014 | 0.502 | -0.942 | 0.943 | 0.002 | 0.003 | 40560.0 | 28907.0 | 1.0 |
| ind[173] | 0.941 | 0.417 | 0.144 | 1.723 | 0.002 | 0.002 | 36497.0 | 28225.0 | 1.0 |
| ind[174] | 0.786 | 0.553 | -0.263 | 1.815 | 0.003 | 0.002 | 39017.0 | 28065.0 | 1.0 |
| ind[175] | -1.147 | 0.572 | -2.214 | -0.065 | 0.003 | 0.002 | 44895.0 | 28454.0 | 1.0 |
| ind[176] | -0.974 | 0.580 | -2.075 | 0.102 | 0.003 | 0.002 | 48860.0 | 29000.0 | 1.0 |
| ind[177] | -0.292 | 0.514 | -1.274 | 0.658 | 0.002 | 0.002 | 44765.0 | 28652.0 | 1.0 |
| ind[178] | 0.188 | 0.603 | -0.955 | 1.312 | 0.003 | 0.003 | 45355.0 | 27462.0 | 1.0 |
| ind[180] | 0.157 | 0.692 | -1.163 | 1.429 | 0.003 | 0.004 | 55526.0 | 28200.0 | 1.0 |
| ind[181] | 1.976 | 0.576 | 0.885 | 3.051 | 0.003 | 0.002 | 40966.0 | 29925.0 | 1.0 |
| ind[182] | -0.167 | 0.481 | -1.076 | 0.731 | 0.002 | 0.002 | 50615.0 | 27822.0 | 1.0 |
| ind[183] | 0.172 | 0.505 | -0.782 | 1.110 | 0.002 | 0.002 | 46023.0 | 29776.0 | 1.0 |
| ind[184] | 1.072 | 0.523 | 0.086 | 2.040 | 0.003 | 0.002 | 38588.0 | 29248.0 | 1.0 |
| ind[185] | 0.364 | 0.778 | -1.089 | 1.808 | 0.004 | 0.004 | 49189.0 | 28672.0 | 1.0 |
| ind[186] | -0.117 | 0.756 | -1.559 | 1.285 | 0.003 | 0.004 | 48348.0 | 29469.0 | 1.0 |
| ind[187] | -1.302 | 0.905 | -2.998 | 0.398 | 0.004 | 0.003 | 60798.0 | 29216.0 | 1.0 |
| ind[188] | -2.215 | 0.757 | -3.661 | -0.803 | 0.003 | 0.003 | 49551.0 | 25274.0 | 1.0 |
| ind[189] | -0.387 | 0.530 | -1.383 | 0.609 | 0.003 | 0.002 | 42886.0 | 27720.0 | 1.0 |
| ind[190] | -0.029 | 0.466 | -0.929 | 0.819 | 0.002 | 0.002 | 40387.0 | 27362.0 | 1.0 |
| ind[191] | 0.304 | 0.491 | -0.637 | 1.217 | 0.002 | 0.002 | 41574.0 | 27825.0 | 1.0 |
| ind[192] | 2.583 | 0.497 | 1.636 | 3.513 | 0.002 | 0.002 | 41400.0 | 28843.0 | 1.0 |
| ind[194] | 1.361 | 0.450 | 0.503 | 2.193 | 0.002 | 0.002 | 42841.0 | 27392.0 | 1.0 |
| ind[195] | -0.313 | 0.819 | -1.879 | 1.196 | 0.003 | 0.004 | 56771.0 | 28144.0 | 1.0 |
| ind[196] | -0.231 | 0.502 | -1.209 | 0.679 | 0.002 | 0.002 | 46780.0 | 28309.0 | 1.0 |
| ind[197] | 0.383 | 0.465 | -0.492 | 1.249 | 0.002 | 0.002 | 42814.0 | 28295.0 | 1.0 |
| ind[198] | -1.517 | 0.885 | -3.197 | 0.123 | 0.004 | 0.003 | 55344.0 | 28713.0 | 1.0 |
| ind[199] | -0.179 | 0.841 | -1.740 | 1.397 | 0.004 | 0.004 | 51806.0 | 29004.0 | 1.0 |
| ind[200] | 0.632 | 0.483 | -0.294 | 1.514 | 0.003 | 0.002 | 35223.0 | 28661.0 | 1.0 |
| ind[201] | 0.487 | 0.478 | -0.408 | 1.388 | 0.002 | 0.002 | 38521.0 | 28546.0 | 1.0 |
| ind[202] | -1.285 | 0.579 | -2.390 | -0.230 | 0.003 | 0.002 | 50405.0 | 28163.0 | 1.0 |
| ind[203] | 0.545 | 0.675 | -0.767 | 1.767 | 0.003 | 0.003 | 51622.0 | 29800.0 | 1.0 |
| ind[204] | 1.305 | 0.451 | 0.450 | 2.151 | 0.002 | 0.002 | 35035.0 | 29771.0 | 1.0 |
| ind[205] | 0.202 | 0.701 | -1.114 | 1.523 | 0.003 | 0.004 | 48194.0 | 28479.0 | 1.0 |
| ind[206] | 0.435 | 0.733 | -0.968 | 1.783 | 0.003 | 0.003 | 65057.0 | 28824.0 | 1.0 |
| ind[207] | -0.181 | 0.474 | -1.089 | 0.689 | 0.002 | 0.002 | 41248.0 | 27041.0 | 1.0 |
| ind[208] | -0.701 | 0.556 | -1.766 | 0.322 | 0.002 | 0.002 | 50684.0 | 27975.0 | 1.0 |
| ind[209] | 0.120 | 0.798 | -1.385 | 1.629 | 0.003 | 0.004 | 57924.0 | 27763.0 | 1.0 |
| ind[210] | -1.784 | 0.671 | -3.047 | -0.533 | 0.003 | 0.002 | 56772.0 | 26649.0 | 1.0 |
| ind[211] | -1.168 | 0.579 | -2.278 | -0.101 | 0.003 | 0.002 | 45306.0 | 26774.0 | 1.0 |
| ind[212] | 1.318 | 0.484 | 0.396 | 2.210 | 0.002 | 0.002 | 37827.0 | 26715.0 | 1.0 |
| ind[213] | -0.040 | 0.493 | -0.946 | 0.907 | 0.002 | 0.003 | 39837.0 | 27649.0 | 1.0 |
| ind[214] | -1.546 | 0.870 | -3.236 | 0.023 | 0.004 | 0.003 | 53539.0 | 28411.0 | 1.0 |
| ind[215] | -0.995 | 0.785 | -2.510 | 0.454 | 0.003 | 0.003 | 63357.0 | 28038.0 | 1.0 |
| ind[216] | 0.254 | 0.449 | -0.603 | 1.081 | 0.002 | 0.002 | 34409.0 | 28381.0 | 1.0 |
| ind[217] | 0.706 | 0.432 | -0.087 | 1.535 | 0.002 | 0.002 | 37098.0 | 27606.0 | 1.0 |
| ind[218] | 0.519 | 0.471 | -0.388 | 1.397 | 0.002 | 0.002 | 38579.0 | 28716.0 | 1.0 |
| ind[219] | 0.379 | 0.479 | -0.524 | 1.281 | 0.002 | 0.002 | 40184.0 | 29910.0 | 1.0 |
| ind[220] | 1.796 | 0.660 | 0.548 | 3.030 | 0.003 | 0.002 | 49753.0 | 30240.0 | 1.0 |
| ind[221] | -1.078 | 0.582 | -2.196 | -0.000 | 0.003 | 0.002 | 50018.0 | 28578.0 | 1.0 |
| ind[222] | -1.424 | 0.613 | -2.574 | -0.281 | 0.003 | 0.002 | 48070.0 | 28119.0 | 1.0 |
| ind[223] | -0.498 | 0.529 | -1.506 | 0.481 | 0.003 | 0.002 | 44877.0 | 29299.0 | 1.0 |
| ind[224] | -0.297 | 0.528 | -1.305 | 0.687 | 0.003 | 0.002 | 36605.0 | 28160.0 | 1.0 |
| ind[225] | -0.255 | 0.457 | -1.098 | 0.608 | 0.002 | 0.002 | 40609.0 | 29535.0 | 1.0 |
| ind[226] | -0.201 | 0.841 | -1.767 | 1.385 | 0.004 | 0.004 | 54919.0 | 27041.0 | 1.0 |
| ind[227] | 1.835 | 0.502 | 0.883 | 2.765 | 0.002 | 0.002 | 42379.0 | 29225.0 | 1.0 |
| ind[228] | 1.864 | 0.456 | 1.017 | 2.731 | 0.002 | 0.002 | 40414.0 | 28401.0 | 1.0 |
| ind[229] | 0.063 | 0.549 | -0.997 | 1.065 | 0.003 | 0.003 | 43688.0 | 27749.0 | 1.0 |
| ind[230] | 0.489 | 0.733 | -0.913 | 1.830 | 0.003 | 0.003 | 47576.0 | 27987.0 | 1.0 |
| ind[231] | 0.585 | 0.501 | -0.370 | 1.509 | 0.002 | 0.002 | 47531.0 | 29454.0 | 1.0 |
| ind[232] | 0.092 | 0.481 | -0.812 | 1.001 | 0.002 | 0.003 | 42727.0 | 27501.0 | 1.0 |
| ind[233] | 0.812 | 0.422 | 0.024 | 1.618 | 0.002 | 0.002 | 38789.0 | 28146.0 | 1.0 |
| ind[234] | -0.505 | 0.530 | -1.484 | 0.510 | 0.002 | 0.002 | 45594.0 | 28103.0 | 1.0 |
| ind[235] | 0.718 | 0.447 | -0.103 | 1.590 | 0.002 | 0.002 | 39844.0 | 29618.0 | 1.0 |
| ind[236] | -0.224 | 0.446 | -1.079 | 0.602 | 0.002 | 0.002 | 46954.0 | 26864.0 | 1.0 |
| ind[237] | 1.049 | 0.455 | 0.199 | 1.915 | 0.002 | 0.002 | 40176.0 | 27951.0 | 1.0 |
| ind[238] | 0.563 | 0.451 | -0.284 | 1.409 | 0.002 | 0.002 | 40225.0 | 27801.0 | 1.0 |
| ind[239] | -1.499 | 0.623 | -2.691 | -0.353 | 0.003 | 0.002 | 46615.0 | 26997.0 | 1.0 |
| ind[240] | -2.338 | 0.742 | -3.756 | -0.989 | 0.003 | 0.003 | 52295.0 | 28706.0 | 1.0 |
| ind[241] | 1.914 | 0.462 | 1.034 | 2.765 | 0.002 | 0.002 | 42592.0 | 28901.0 | 1.0 |
| ind[242] | 1.537 | 0.444 | 0.679 | 2.352 | 0.002 | 0.002 | 41266.0 | 28159.0 | 1.0 |
| ind[244] | 1.032 | 0.470 | 0.125 | 1.897 | 0.002 | 0.002 | 40788.0 | 29005.0 | 1.0 |
| ind[245] | -0.695 | 0.492 | -1.630 | 0.221 | 0.002 | 0.002 | 49676.0 | 27449.0 | 1.0 |
| ind[246] | -0.882 | 0.596 | -2.026 | 0.227 | 0.003 | 0.002 | 47492.0 | 28087.0 | 1.0 |
| ind[247] | 0.187 | 0.474 | -0.690 | 1.087 | 0.002 | 0.002 | 44083.0 | 28070.0 | 1.0 |
| ind[248] | -0.262 | 0.679 | -1.574 | 0.975 | 0.003 | 0.003 | 47256.0 | 29484.0 | 1.0 |
| ind[249] | 0.286 | 0.502 | -0.657 | 1.223 | 0.002 | 0.002 | 49369.0 | 29927.0 | 1.0 |
| ind[250] | -1.550 | 0.616 | -2.713 | -0.414 | 0.003 | 0.002 | 56559.0 | 29617.0 | 1.0 |
| ind[251] | -0.365 | 0.511 | -1.306 | 0.611 | 0.003 | 0.002 | 35861.0 | 28603.0 | 1.0 |
| ind[252] | 0.940 | 0.446 | 0.090 | 1.763 | 0.002 | 0.002 | 39692.0 | 27394.0 | 1.0 |
| ind[253] | 0.009 | 0.489 | -0.910 | 0.933 | 0.002 | 0.002 | 41139.0 | 28879.0 | 1.0 |
| ind[254] | -0.568 | 0.520 | -1.541 | 0.411 | 0.003 | 0.002 | 39146.0 | 27658.0 | 1.0 |
| ind[255] | -1.254 | 0.644 | -2.450 | -0.024 | 0.003 | 0.002 | 57486.0 | 29263.0 | 1.0 |
| ind[256] | -0.521 | 0.859 | -2.182 | 1.054 | 0.004 | 0.004 | 57027.0 | 27462.0 | 1.0 |
| ind[257] | 2.313 | 0.476 | 1.437 | 3.227 | 0.002 | 0.002 | 40133.0 | 29772.0 | 1.0 |
| ind[258] | -0.427 | 0.829 | -1.992 | 1.116 | 0.004 | 0.004 | 53479.0 | 29346.0 | 1.0 |
| ind[259] | 0.450 | 0.478 | -0.456 | 1.345 | 0.002 | 0.002 | 39492.0 | 28555.0 | 1.0 |
| ind[260] | 0.291 | 0.445 | -0.549 | 1.123 | 0.002 | 0.002 | 43666.0 | 27531.0 | 1.0 |
| ind[261] | 3.793 | 0.422 | 3.002 | 4.587 | 0.002 | 0.001 | 41890.0 | 29721.0 | 1.0 |
| ind[262] | 0.673 | 0.492 | -0.264 | 1.587 | 0.002 | 0.002 | 41424.0 | 28928.0 | 1.0 |
| ind[263] | -0.640 | 0.546 | -1.689 | 0.367 | 0.003 | 0.002 | 41401.0 | 29178.0 | 1.0 |
| ind[264] | -1.515 | 0.885 | -3.168 | 0.170 | 0.004 | 0.003 | 63389.0 | 27710.0 | 1.0 |
| ind[265] | -0.021 | 0.500 | -0.981 | 0.907 | 0.002 | 0.003 | 40760.0 | 28404.0 | 1.0 |
| ind[266] | -0.491 | 0.486 | -1.391 | 0.431 | 0.002 | 0.002 | 44034.0 | 27585.0 | 1.0 |
| ind[267] | -0.105 | 0.511 | -1.051 | 0.863 | 0.002 | 0.003 | 42455.0 | 28151.0 | 1.0 |
| ind[268] | -0.495 | 0.519 | -1.485 | 0.455 | 0.003 | 0.002 | 35478.0 | 27954.0 | 1.0 |
| ind[269] | -1.190 | 0.921 | -2.945 | 0.507 | 0.004 | 0.004 | 58788.0 | 27334.0 | 1.0 |
| ind[270] | -1.540 | 0.613 | -2.712 | -0.408 | 0.003 | 0.002 | 49523.0 | 27690.0 | 1.0 |
| ind[271] | 0.102 | 0.774 | -1.369 | 1.538 | 0.003 | 0.004 | 55407.0 | 26725.0 | 1.0 |
| ind[272] | 0.089 | 0.436 | -0.738 | 0.906 | 0.002 | 0.002 | 31788.0 | 29545.0 | 1.0 |
| ind[273] | 0.400 | 0.715 | -0.934 | 1.782 | 0.003 | 0.003 | 44854.0 | 29128.0 | 1.0 |
| ind[274] | 1.333 | 0.485 | 0.443 | 2.267 | 0.003 | 0.002 | 32323.0 | 29349.0 | 1.0 |
| ind[275] | 1.456 | 0.446 | 0.596 | 2.276 | 0.002 | 0.002 | 35493.0 | 27366.0 | 1.0 |
| ind[276] | -1.143 | 0.534 | -2.162 | -0.160 | 0.003 | 0.002 | 44789.0 | 30278.0 | 1.0 |
| ind[277] | 0.854 | 0.484 | -0.042 | 1.786 | 0.002 | 0.002 | 40347.0 | 26714.0 | 1.0 |
| ind[278] | -0.003 | 0.442 | -0.828 | 0.837 | 0.002 | 0.002 | 35391.0 | 27984.0 | 1.0 |
| ind[279] | -0.891 | 0.803 | -2.418 | 0.597 | 0.003 | 0.003 | 60209.0 | 28923.0 | 1.0 |
| ind[280] | 0.448 | 0.490 | -0.447 | 1.396 | 0.003 | 0.002 | 34585.0 | 28017.0 | 1.0 |
| ind[281] | -0.754 | 0.522 | -1.752 | 0.207 | 0.003 | 0.002 | 35545.0 | 27145.0 | 1.0 |
| ind[282] | 0.201 | 0.544 | -0.859 | 1.182 | 0.003 | 0.003 | 36632.0 | 28301.0 | 1.0 |
| ind[283] | 0.507 | 0.453 | -0.346 | 1.368 | 0.002 | 0.002 | 33870.0 | 28427.0 | 1.0 |
| ind[285] | 0.900 | 0.421 | 0.106 | 1.688 | 0.002 | 0.002 | 35015.0 | 26488.0 | 1.0 |
| ind[286] | -1.103 | 0.588 | -2.219 | -0.021 | 0.003 | 0.002 | 42913.0 | 28730.0 | 1.0 |
| ind[287] | 1.102 | 0.429 | 0.304 | 1.924 | 0.002 | 0.002 | 34598.0 | 28078.0 | 1.0 |
| ind[288] | 0.141 | 0.975 | -1.667 | 1.998 | 0.004 | 0.005 | 60002.0 | 28425.0 | 1.0 |
| ind[289] | -0.186 | 0.505 | -1.156 | 0.744 | 0.003 | 0.002 | 38197.0 | 28261.0 | 1.0 |
| ind[290] | -0.317 | 0.751 | -1.754 | 1.056 | 0.003 | 0.004 | 58261.0 | 29097.0 | 1.0 |
| ind[291] | -1.558 | 0.864 | -3.158 | 0.082 | 0.004 | 0.003 | 46089.0 | 28403.0 | 1.0 |
| ind[292] | 1.620 | 0.478 | 0.725 | 2.529 | 0.002 | 0.002 | 39776.0 | 29522.0 | 1.0 |
| ind[293] | -0.350 | 0.499 | -1.282 | 0.597 | 0.002 | 0.002 | 40940.0 | 28751.0 | 1.0 |
| ind[294] | -1.316 | 0.908 | -3.073 | 0.328 | 0.004 | 0.004 | 49688.0 | 28588.0 | 1.0 |
| ind[295] | -0.343 | 0.739 | -1.769 | 1.003 | 0.003 | 0.004 | 59558.0 | 27129.0 | 1.0 |
| ind[296] | 0.946 | 0.769 | -0.518 | 2.374 | 0.003 | 0.003 | 54550.0 | 29674.0 | 1.0 |
| ind[297] | 0.061 | 0.767 | -1.412 | 1.473 | 0.003 | 0.004 | 55941.0 | 28453.0 | 1.0 |
| ind[298] | -0.216 | 0.749 | -1.654 | 1.167 | 0.004 | 0.004 | 41929.0 | 28488.0 | 1.0 |
| ind[299] | -2.174 | 0.641 | -3.376 | -0.984 | 0.003 | 0.002 | 54879.0 | 26284.0 | 1.0 |
| ind[300] | 0.869 | 0.487 | -0.025 | 1.805 | 0.002 | 0.002 | 42632.0 | 29472.0 | 1.0 |
| ind[301] | 2.180 | 0.532 | 1.176 | 3.181 | 0.003 | 0.002 | 37806.0 | 30164.0 | 1.0 |
| ind[302] | -0.196 | 0.507 | -1.124 | 0.777 | 0.003 | 0.002 | 39210.0 | 28437.0 | 1.0 |
| ind[304] | -0.527 | 0.615 | -1.679 | 0.617 | 0.003 | 0.003 | 44599.0 | 28952.0 | 1.0 |
| ind[305] | -0.537 | 0.534 | -1.560 | 0.450 | 0.003 | 0.002 | 45783.0 | 27356.0 | 1.0 |
| ind[306] | 0.695 | 0.698 | -0.592 | 2.042 | 0.003 | 0.003 | 49304.0 | 27991.0 | 1.0 |
| ind[307] | -2.080 | 0.775 | -3.568 | -0.669 | 0.004 | 0.003 | 45931.0 | 29003.0 | 1.0 |
| ind[308] | 0.740 | 0.564 | -0.298 | 1.822 | 0.003 | 0.002 | 40875.0 | 29204.0 | 1.0 |
| ind[309] | -0.804 | 1.074 | -2.781 | 1.277 | 0.004 | 0.005 | 58150.0 | 28841.0 | 1.0 |
| ind[310] | -0.802 | 0.560 | -1.863 | 0.236 | 0.003 | 0.002 | 38045.0 | 29244.0 | 1.0 |
| ind[311] | 2.304 | 0.416 | 1.507 | 3.074 | 0.002 | 0.002 | 34441.0 | 29571.0 | 1.0 |
| ind[312] | 1.466 | 0.793 | -0.014 | 2.955 | 0.004 | 0.003 | 48099.0 | 29047.0 | 1.0 |
| ind[313] | -0.316 | 0.484 | -1.243 | 0.588 | 0.002 | 0.002 | 39119.0 | 28821.0 | 1.0 |
| ind[314] | -0.308 | 0.749 | -1.747 | 1.070 | 0.003 | 0.004 | 46315.0 | 28428.0 | 1.0 |
| ind[315] | -0.976 | 0.537 | -1.980 | 0.025 | 0.003 | 0.002 | 37012.0 | 28639.0 | 1.0 |
| ind[316] | -0.353 | 0.481 | -1.240 | 0.565 | 0.002 | 0.002 | 44250.0 | 27992.0 | 1.0 |
| ind[317] | 0.074 | 0.493 | -0.848 | 1.014 | 0.002 | 0.003 | 45465.0 | 27340.0 | 1.0 |
| ind[318] | -1.033 | 0.638 | -2.213 | 0.182 | 0.003 | 0.003 | 39841.0 | 27474.0 | 1.0 |
| ind[319] | -0.359 | 0.511 | -1.303 | 0.627 | 0.002 | 0.002 | 51544.0 | 28210.0 | 1.0 |
| ind[320] | 0.439 | 0.444 | -0.418 | 1.254 | 0.002 | 0.002 | 45388.0 | 29544.0 | 1.0 |
| ind[321] | -0.500 | 0.482 | -1.392 | 0.419 | 0.002 | 0.002 | 43153.0 | 29227.0 | 1.0 |
| ind[322] | -2.125 | 0.760 | -3.561 | -0.708 | 0.003 | 0.003 | 59767.0 | 25485.0 | 1.0 |
| ind[323] | -1.713 | 0.609 | -2.900 | -0.614 | 0.002 | 0.002 | 61852.0 | 26816.0 | 1.0 |
| ind[324] | 0.812 | 0.417 | 0.042 | 1.598 | 0.002 | 0.002 | 42485.0 | 28349.0 | 1.0 |
| ind[325] | 0.833 | 0.545 | -0.199 | 1.863 | 0.003 | 0.002 | 42874.0 | 29423.0 | 1.0 |
| ind[326] | -0.492 | 0.514 | -1.466 | 0.466 | 0.002 | 0.002 | 50088.0 | 28308.0 | 1.0 |
| ind[327] | 0.010 | 0.451 | -0.818 | 0.878 | 0.002 | 0.002 | 44332.0 | 29388.0 | 1.0 |
| ind[328] | 1.016 | 0.715 | -0.347 | 2.343 | 0.003 | 0.002 | 68821.0 | 28470.0 | 1.0 |
| ind[329] | -0.536 | 0.526 | -1.538 | 0.448 | 0.003 | 0.002 | 41994.0 | 28880.0 | 1.0 |
| ind[330] | -0.422 | 0.503 | -1.372 | 0.516 | 0.002 | 0.002 | 41344.0 | 28260.0 | 1.0 |
| ind[331] | -0.112 | 0.758 | -1.524 | 1.344 | 0.003 | 0.004 | 55326.0 | 29211.0 | 1.0 |
| ind[332] | -0.584 | 0.837 | -2.142 | 0.996 | 0.004 | 0.004 | 52556.0 | 28812.0 | 1.0 |
| ind[333] | 1.823 | 0.405 | 1.044 | 2.570 | 0.002 | 0.001 | 39079.0 | 27097.0 | 1.0 |
| ind[334] | 1.439 | 0.429 | 0.647 | 2.256 | 0.002 | 0.002 | 36193.0 | 28819.0 | 1.0 |
| ind[335] | 0.434 | 0.474 | -0.452 | 1.334 | 0.002 | 0.002 | 39496.0 | 28675.0 | 1.0 |
| ind[336] | 1.284 | 0.741 | -0.118 | 2.660 | 0.003 | 0.003 | 47474.0 | 29153.0 | 1.0 |
| ind[337] | -0.030 | 0.482 | -0.943 | 0.871 | 0.002 | 0.002 | 39629.0 | 29191.0 | 1.0 |
| ind[338] | -0.195 | 0.478 | -1.094 | 0.700 | 0.002 | 0.002 | 42696.0 | 27970.0 | 1.0 |
| ind[339] | -0.030 | 0.512 | -1.032 | 0.898 | 0.002 | 0.003 | 47741.0 | 30638.0 | 1.0 |
| ind[340] | 1.205 | 0.498 | 0.282 | 2.151 | 0.003 | 0.002 | 37561.0 | 27838.0 | 1.0 |
| ind[341] | -0.590 | 0.527 | -1.589 | 0.394 | 0.002 | 0.002 | 53757.0 | 27902.0 | 1.0 |
| ind[342] | -0.966 | 0.582 | -2.066 | 0.114 | 0.003 | 0.002 | 42085.0 | 27608.0 | 1.0 |
| ind[343] | -0.568 | 0.823 | -2.145 | 0.926 | 0.004 | 0.004 | 49632.0 | 29473.0 | 1.0 |
| ind[344] | -0.370 | 0.498 | -1.307 | 0.570 | 0.002 | 0.002 | 44689.0 | 28814.0 | 1.0 |
| ind[345] | -1.650 | 0.692 | -2.939 | -0.357 | 0.003 | 0.003 | 48704.0 | 26249.0 | 1.0 |
| ind[346] | 1.114 | 0.442 | 0.264 | 1.928 | 0.002 | 0.002 | 39535.0 | 29172.0 | 1.0 |
| ind[347] | -1.828 | 0.668 | -3.108 | -0.599 | 0.003 | 0.002 | 47795.0 | 28030.0 | 1.0 |
| ind[348] | 1.992 | 0.427 | 1.157 | 2.758 | 0.002 | 0.002 | 36711.0 | 29268.0 | 1.0 |
| ind[349] | -1.098 | 0.576 | -2.184 | -0.027 | 0.003 | 0.002 | 45404.0 | 27769.0 | 1.0 |
| ind[350] | -1.038 | 0.528 | -2.043 | -0.072 | 0.003 | 0.002 | 44698.0 | 28835.0 | 1.0 |
| ind[351] | -0.067 | 0.863 | -1.689 | 1.548 | 0.004 | 0.005 | 59888.0 | 28554.0 | 1.0 |
| ind[352] | -0.899 | 0.543 | -1.934 | 0.101 | 0.003 | 0.002 | 39068.0 | 29650.0 | 1.0 |
| ind[353] | -0.854 | 0.558 | -1.916 | 0.179 | 0.003 | 0.002 | 43528.0 | 27792.0 | 1.0 |
| ind[354] | 0.918 | 0.471 | 0.027 | 1.798 | 0.002 | 0.002 | 44410.0 | 27349.0 | 1.0 |
| ind[355] | -1.270 | 0.577 | -2.410 | -0.231 | 0.003 | 0.002 | 46264.0 | 28820.0 | 1.0 |
| ind[356] | -1.239 | 0.908 | -2.958 | 0.435 | 0.004 | 0.003 | 59626.0 | 29295.0 | 1.0 |
| ind[357] | -0.170 | 0.499 | -1.118 | 0.759 | 0.002 | 0.002 | 44744.0 | 27480.0 | 1.0 |
| ind[358] | -0.725 | 0.814 | -2.300 | 0.779 | 0.003 | 0.004 | 55022.0 | 28162.0 | 1.0 |
| ind[359] | -0.166 | 0.565 | -1.202 | 0.905 | 0.003 | 0.003 | 43544.0 | 27345.0 | 1.0 |
| ind[360] | 0.440 | 0.713 | -0.938 | 1.748 | 0.003 | 0.003 | 63983.0 | 28810.0 | 1.0 |
| ind[361] | -1.480 | 0.893 | -3.150 | 0.180 | 0.004 | 0.003 | 53705.0 | 29791.0 | 1.0 |
| ind[362] | -1.314 | 0.901 | -3.009 | 0.366 | 0.004 | 0.003 | 60273.0 | 28883.0 | 1.0 |
| ind[363] | 0.188 | 0.779 | -1.285 | 1.641 | 0.003 | 0.004 | 52156.0 | 29389.0 | 1.0 |
| ind[364] | 0.866 | 0.726 | -0.462 | 2.259 | 0.003 | 0.003 | 59716.0 | 27421.0 | 1.0 |
| ind[365] | -1.484 | 0.891 | -3.175 | 0.160 | 0.004 | 0.003 | 53182.0 | 27547.0 | 1.0 |
| ind[366] | 0.001 | 0.446 | -0.841 | 0.831 | 0.002 | 0.002 | 36866.0 | 28645.0 | 1.0 |
| ind[367] | -0.405 | 0.494 | -1.339 | 0.510 | 0.002 | 0.002 | 40497.0 | 29694.0 | 1.0 |
| ind[368] | -0.372 | 0.524 | -1.367 | 0.613 | 0.002 | 0.002 | 44527.0 | 27531.0 | 1.0 |
| ind[369] | 0.808 | 0.462 | -0.075 | 1.659 | 0.002 | 0.002 | 40887.0 | 28850.0 | 1.0 |
| ind[370] | -1.686 | 0.604 | -2.820 | -0.548 | 0.003 | 0.002 | 56086.0 | 26804.0 | 1.0 |
| ind[371] | -1.085 | 0.576 | -2.193 | -0.030 | 0.003 | 0.002 | 46225.0 | 27621.0 | 1.0 |
| ind[373] | 0.593 | 0.484 | -0.298 | 1.520 | 0.002 | 0.002 | 40335.0 | 27848.0 | 1.0 |
| ind[374] | 0.568 | 0.432 | -0.240 | 1.386 | 0.002 | 0.002 | 39732.0 | 27380.0 | 1.0 |
| ind[375] | 0.067 | 0.786 | -1.429 | 1.536 | 0.004 | 0.004 | 48013.0 | 29568.0 | 1.0 |
| ind[376] | -2.522 | 0.711 | -3.863 | -1.203 | 0.003 | 0.002 | 56077.0 | 28276.0 | 1.0 |
| ind[377] | -0.719 | 0.515 | -1.711 | 0.227 | 0.003 | 0.002 | 40678.0 | 25319.0 | 1.0 |
| ind[378] | 1.603 | 0.445 | 0.795 | 2.470 | 0.002 | 0.002 | 37571.0 | 27988.0 | 1.0 |
| ind[379] | 1.832 | 0.437 | 1.015 | 2.663 | 0.002 | 0.002 | 33551.0 | 27550.0 | 1.0 |
| ind[380] | -0.194 | 0.837 | -1.764 | 1.389 | 0.003 | 0.005 | 59596.0 | 26489.0 | 1.0 |
| ind[381] | 0.677 | 0.494 | -0.282 | 1.586 | 0.002 | 0.002 | 48671.0 | 28568.0 | 1.0 |
| ind[382] | 1.056 | 0.455 | 0.200 | 1.906 | 0.002 | 0.002 | 36809.0 | 27359.0 | 1.0 |
| ind[383] | -0.662 | 0.513 | -1.631 | 0.301 | 0.003 | 0.002 | 39385.0 | 29036.0 | 1.0 |
| ind[384] | -0.041 | 0.490 | -0.960 | 0.878 | 0.003 | 0.003 | 32546.0 | 26757.0 | 1.0 |
| ind[385] | -0.450 | 0.837 | -2.040 | 1.097 | 0.004 | 0.004 | 49198.0 | 28930.0 | 1.0 |
| ind[386] | 0.584 | 0.753 | -0.830 | 2.004 | 0.003 | 0.003 | 55377.0 | 28028.0 | 1.0 |
| ind[387] | 0.175 | 0.744 | -1.283 | 1.520 | 0.003 | 0.004 | 52619.0 | 28617.0 | 1.0 |
| ind[388] | -2.211 | 0.763 | -3.676 | -0.818 | 0.004 | 0.003 | 46876.0 | 25719.0 | 1.0 |
| ind[389] | 1.018 | 0.467 | 0.129 | 1.891 | 0.002 | 0.002 | 43434.0 | 28477.0 | 1.0 |
| ind[390] | -1.548 | 0.861 | -3.172 | 0.048 | 0.004 | 0.003 | 60368.0 | 29114.0 | 1.0 |
| ind[391] | -0.310 | 0.478 | -1.217 | 0.576 | 0.003 | 0.002 | 33604.0 | 26222.0 | 1.0 |
| ind[392] | -0.080 | 0.484 | -0.997 | 0.823 | 0.002 | 0.003 | 44421.0 | 28603.0 | 1.0 |
| ind[393] | 0.461 | 0.472 | -0.427 | 1.342 | 0.002 | 0.002 | 44299.0 | 28938.0 | 1.0 |
| ind[394] | 1.108 | 0.686 | -0.182 | 2.383 | 0.003 | 0.002 | 51186.0 | 29585.0 | 1.0 |
| ind[395] | 0.052 | 0.782 | -1.436 | 1.505 | 0.003 | 0.004 | 60754.0 | 29521.0 | 1.0 |
| ind[396] | 1.326 | 0.495 | 0.387 | 2.252 | 0.003 | 0.002 | 35702.0 | 28777.0 | 1.0 |
| ind[397] | 1.008 | 0.430 | 0.201 | 1.821 | 0.002 | 0.002 | 39920.0 | 28961.0 | 1.0 |
| ind[399] | -1.248 | 0.616 | -2.441 | -0.136 | 0.003 | 0.002 | 48079.0 | 28627.0 | 1.0 |
| ind[400] | 0.524 | 0.455 | -0.332 | 1.374 | 0.002 | 0.002 | 39022.0 | 27186.0 | 1.0 |
| ind[401] | -1.089 | 0.580 | -2.180 | -0.009 | 0.003 | 0.002 | 52453.0 | 27095.0 | 1.0 |
| ind[402] | 1.504 | 0.482 | 0.604 | 2.404 | 0.003 | 0.002 | 36299.0 | 26825.0 | 1.0 |
| ind[403] | 0.004 | 1.211 | -2.256 | 2.304 | 0.005 | 0.006 | 53480.0 | 29559.0 | 1.0 |
| ind[404] | 0.602 | 0.458 | -0.236 | 1.478 | 0.002 | 0.002 | 35944.0 | 26988.0 | 1.0 |
| ind[405] | -1.827 | 0.673 | -3.110 | -0.585 | 0.003 | 0.003 | 46688.0 | 27680.0 | 1.0 |
| ind[406] | -0.317 | 0.525 | -1.322 | 0.662 | 0.003 | 0.003 | 42086.0 | 27687.0 | 1.0 |
| ind[407] | 0.900 | 0.432 | 0.098 | 1.720 | 0.002 | 0.002 | 37311.0 | 29308.0 | 1.0 |
| ind[408] | -0.649 | 0.542 | -1.635 | 0.412 | 0.003 | 0.002 | 47240.0 | 27874.0 | 1.0 |
| ind[409] | 0.894 | 0.412 | 0.118 | 1.670 | 0.002 | 0.002 | 38877.0 | 28644.0 | 1.0 |
| ind[410] | 0.233 | 0.476 | -0.674 | 1.118 | 0.003 | 0.002 | 35426.0 | 27949.0 | 1.0 |
| ind[411] | -1.550 | 0.696 | -2.900 | -0.292 | 0.003 | 0.003 | 43711.0 | 28089.0 | 1.0 |
| ind[412] | 0.988 | 0.440 | 0.180 | 1.822 | 0.002 | 0.002 | 36115.0 | 28458.0 | 1.0 |
| ind[413] | 0.741 | 0.491 | -0.183 | 1.663 | 0.002 | 0.002 | 47727.0 | 28325.0 | 1.0 |
| ind[414] | -0.052 | 0.511 | -1.028 | 0.887 | 0.002 | 0.003 | 42017.0 | 26909.0 | 1.0 |
| ind[415] | 1.123 | 0.436 | 0.274 | 1.911 | 0.002 | 0.002 | 33861.0 | 27686.0 | 1.0 |
| ind[416] | -1.262 | 0.619 | -2.479 | -0.159 | 0.003 | 0.002 | 45798.0 | 27852.0 | 1.0 |
| ind[417] | 0.901 | 0.723 | -0.429 | 2.295 | 0.003 | 0.003 | 47531.0 | 27522.0 | 1.0 |
| ind[418] | -1.043 | 0.546 | -2.094 | -0.031 | 0.003 | 0.002 | 47293.0 | 27588.0 | 1.0 |
| ind[419] | 0.396 | 0.729 | -0.964 | 1.781 | 0.004 | 0.003 | 43579.0 | 27723.0 | 1.0 |
| ind[420] | -0.638 | 0.811 | -2.173 | 0.859 | 0.003 | 0.004 | 57386.0 | 27334.0 | 1.0 |
| ind[421] | 1.365 | 0.417 | 0.567 | 2.128 | 0.002 | 0.001 | 39609.0 | 30061.0 | 1.0 |
| ind[422] | 0.557 | 0.498 | -0.386 | 1.478 | 0.002 | 0.002 | 42775.0 | 28847.0 | 1.0 |
| ind[423] | -0.510 | 0.831 | -2.057 | 1.070 | 0.003 | 0.004 | 60996.0 | 28493.0 | 1.0 |
| ind[424] | -0.364 | 0.507 | -1.295 | 0.616 | 0.002 | 0.002 | 42800.0 | 28203.0 | 1.0 |
| ind[425] | 0.344 | 0.520 | -0.628 | 1.326 | 0.003 | 0.002 | 38350.0 | 29129.0 | 1.0 |
| ind[426] | -0.698 | 0.544 | -1.751 | 0.292 | 0.003 | 0.002 | 46538.0 | 28247.0 | 1.0 |
| ind[427] | -0.387 | 0.528 | -1.388 | 0.597 | 0.002 | 0.002 | 45175.0 | 28031.0 | 1.0 |
| ind[428] | -0.193 | 0.527 | -1.208 | 0.778 | 0.003 | 0.003 | 38780.0 | 27729.0 | 1.0 |
| ind[429] | -0.795 | 0.813 | -2.315 | 0.728 | 0.004 | 0.003 | 53432.0 | 28002.0 | 1.0 |
| ind[430] | -0.134 | 0.470 | -1.022 | 0.734 | 0.002 | 0.002 | 46774.0 | 29212.0 | 1.0 |
| ind[431] | -0.500 | 0.728 | -1.888 | 0.834 | 0.003 | 0.003 | 50585.0 | 27906.0 | 1.0 |
| ind[432] | -1.179 | 0.920 | -2.928 | 0.521 | 0.004 | 0.004 | 58979.0 | 27753.0 | 1.0 |
| ind[433] | -1.010 | 0.539 | -2.033 | -0.017 | 0.003 | 0.002 | 46350.0 | 27160.0 | 1.0 |
| ind[434] | -0.426 | 0.490 | -1.342 | 0.500 | 0.002 | 0.002 | 43281.0 | 28706.0 | 1.0 |
| ind[435] | 1.637 | 0.434 | 0.829 | 2.469 | 0.002 | 0.002 | 31911.0 | 27990.0 | 1.0 |
| ind[436] | -1.813 | 0.673 | -3.056 | -0.546 | 0.003 | 0.002 | 48336.0 | 29045.0 | 1.0 |
| ind[437] | 0.126 | 0.984 | -1.733 | 1.963 | 0.004 | 0.005 | 66483.0 | 30209.0 | 1.0 |
| ind[438] | 0.778 | 0.472 | -0.124 | 1.656 | 0.002 | 0.002 | 40603.0 | 27878.0 | 1.0 |
| ind[439] | -0.510 | 0.567 | -1.571 | 0.555 | 0.003 | 0.002 | 49802.0 | 29060.0 | 1.0 |
| ind[440] | -1.781 | 0.847 | -3.347 | -0.183 | 0.004 | 0.003 | 52256.0 | 29873.0 | 1.0 |
| ind[441] | 1.669 | 0.447 | 0.803 | 2.485 | 0.002 | 0.002 | 37887.0 | 28907.0 | 1.0 |
| ind[442] | 1.152 | 0.429 | 0.352 | 1.962 | 0.002 | 0.002 | 38366.0 | 29595.0 | 1.0 |
| ind[443] | -0.327 | 0.492 | -1.271 | 0.570 | 0.002 | 0.002 | 47209.0 | 27397.0 | 1.0 |
| ind[444] | -0.695 | 0.711 | -2.025 | 0.648 | 0.003 | 0.003 | 51083.0 | 28900.0 | 1.0 |
| ind[445] | -0.715 | 1.069 | -2.778 | 1.260 | 0.005 | 0.005 | 48059.0 | 30116.0 | 1.0 |
| ind[446] | 0.822 | 0.552 | -0.203 | 1.867 | 0.003 | 0.002 | 45740.0 | 28072.0 | 1.0 |
| ind[447] | -2.173 | 0.663 | -3.435 | -0.957 | 0.003 | 0.002 | 49288.0 | 26924.0 | 1.0 |
| ind[448] | 0.218 | 0.466 | -0.682 | 1.065 | 0.003 | 0.002 | 32530.0 | 29741.0 | 1.0 |
| ind[449] | -1.048 | 0.588 | -2.157 | 0.036 | 0.003 | 0.002 | 44327.0 | 26987.0 | 1.0 |
| ind[450] | -1.783 | 0.680 | -3.083 | -0.524 | 0.003 | 0.002 | 61868.0 | 26456.0 | 1.0 |
| ind[451] | 2.016 | 0.435 | 1.185 | 2.827 | 0.002 | 0.002 | 36293.0 | 31179.0 | 1.0 |
| ind[452] | -0.005 | 1.209 | -2.240 | 2.279 | 0.005 | 0.006 | 52461.0 | 30500.0 | 1.0 |
| ind[454] | -0.050 | 0.476 | -0.940 | 0.848 | 0.002 | 0.002 | 40505.0 | 29061.0 | 1.0 |
| ind[455] | 0.016 | 0.473 | -0.880 | 0.895 | 0.002 | 0.003 | 48209.0 | 27667.0 | 1.0 |
| ind[456] | 1.113 | 0.446 | 0.261 | 1.933 | 0.002 | 0.002 | 36849.0 | 28937.0 | 1.0 |
| ind[457] | 0.678 | 0.474 | -0.206 | 1.582 | 0.003 | 0.002 | 32607.0 | 27877.0 | 1.0 |
| ind[458] | 0.458 | 0.478 | -0.450 | 1.350 | 0.002 | 0.002 | 47917.0 | 28569.0 | 1.0 |
| ind[459] | 1.877 | 0.529 | 0.885 | 2.879 | 0.003 | 0.002 | 39525.0 | 29975.0 | 1.0 |
| ind[460] | -0.139 | 0.482 | -1.059 | 0.760 | 0.002 | 0.003 | 45762.0 | 29378.0 | 1.0 |
| ind[461] | 0.610 | 0.412 | -0.169 | 1.365 | 0.002 | 0.002 | 40548.0 | 29145.0 | 1.0 |
| ind[462] | -0.722 | 1.064 | -2.685 | 1.298 | 0.005 | 0.005 | 53554.0 | 28355.0 | 1.0 |
| ind[463] | 0.146 | 0.993 | -1.705 | 2.025 | 0.004 | 0.005 | 64076.0 | 28617.0 | 1.0 |
| ind[464] | -0.493 | 0.562 | -1.539 | 0.568 | 0.003 | 0.002 | 45943.0 | 26940.0 | 1.0 |
| ind[465] | -0.035 | 0.467 | -0.912 | 0.840 | 0.002 | 0.002 | 37054.0 | 28820.0 | 1.0 |
| ind[466] | -1.325 | 0.919 | -3.074 | 0.351 | 0.004 | 0.003 | 58322.0 | 28023.0 | 1.0 |
| ind[467] | 0.135 | 0.989 | -1.711 | 2.012 | 0.004 | 0.005 | 61372.0 | 29953.0 | 1.0 |
| ind[468] | -1.389 | 0.573 | -2.488 | -0.340 | 0.003 | 0.002 | 45899.0 | 27308.0 | 1.0 |
| ind[469] | -1.230 | 0.767 | -2.667 | 0.210 | 0.003 | 0.003 | 57204.0 | 27273.0 | 1.0 |
| ind[470] | 1.231 | 0.413 | 0.444 | 2.005 | 0.002 | 0.002 | 41723.0 | 27295.0 | 1.0 |
| ind[471] | -1.183 | 0.786 | -2.694 | 0.249 | 0.004 | 0.003 | 50415.0 | 28531.0 | 1.0 |
| ind[472] | -0.655 | 0.541 | -1.669 | 0.370 | 0.003 | 0.002 | 37435.0 | 28472.0 | 1.0 |
| ind[473] | -0.210 | 0.501 | -1.166 | 0.716 | 0.002 | 0.002 | 42601.0 | 28525.0 | 1.0 |
| ind[474] | -1.089 | 0.792 | -2.563 | 0.404 | 0.004 | 0.003 | 51024.0 | 26838.0 | 1.0 |
| ind[475] | 0.744 | 0.715 | -0.629 | 2.066 | 0.003 | 0.003 | 53190.0 | 29128.0 | 1.0 |
| ind[476] | -1.111 | 0.637 | -2.317 | 0.065 | 0.003 | 0.002 | 50240.0 | 25750.0 | 1.0 |
| ind[477] | -0.690 | 0.496 | -1.631 | 0.239 | 0.003 | 0.002 | 39285.0 | 28996.0 | 1.0 |
| ind[478] | -0.073 | 0.729 | -1.434 | 1.304 | 0.003 | 0.004 | 50954.0 | 29094.0 | 1.0 |
| ind[479] | -0.705 | 1.059 | -2.675 | 1.301 | 0.004 | 0.005 | 68791.0 | 30335.0 | 1.0 |
| ind[480] | -0.693 | 0.503 | -1.653 | 0.232 | 0.002 | 0.002 | 44980.0 | 27616.0 | 1.0 |
| ind[481] | 0.357 | 0.491 | -0.577 | 1.264 | 0.002 | 0.002 | 43622.0 | 28656.0 | 1.0 |
| ind[482] | 0.198 | 0.991 | -1.656 | 2.064 | 0.004 | 0.005 | 61925.0 | 31038.0 | 1.0 |
| ind[483] | -0.432 | 0.531 | -1.475 | 0.529 | 0.003 | 0.002 | 44887.0 | 29261.0 | 1.0 |
| ind[484] | 0.151 | 0.476 | -0.733 | 1.056 | 0.002 | 0.002 | 44197.0 | 28906.0 | 1.0 |
| ind[485] | 1.643 | 0.423 | 0.853 | 2.436 | 0.002 | 0.002 | 37001.0 | 28556.0 | 1.0 |
| ind[486] | 0.982 | 0.465 | 0.094 | 1.826 | 0.002 | 0.002 | 39973.0 | 27595.0 | 1.0 |
| ind[487] | -1.405 | 0.566 | -2.473 | -0.348 | 0.003 | 0.002 | 45453.0 | 27515.0 | 1.0 |
| ind[488] | 1.378 | 0.441 | 0.565 | 2.229 | 0.002 | 0.002 | 40498.0 | 28521.0 | 1.0 |
| ind[489] | 0.795 | 0.436 | -0.028 | 1.611 | 0.002 | 0.002 | 36061.0 | 28097.0 | 1.0 |
| ind[490] | 2.709 | 0.612 | 1.552 | 3.843 | 0.003 | 0.002 | 43212.0 | 29406.0 | 1.0 |
| ind[491] | 1.302 | 0.432 | 0.480 | 2.103 | 0.002 | 0.002 | 36712.0 | 28752.0 | 1.0 |
| ind[492] | 0.222 | 0.988 | -1.623 | 2.126 | 0.004 | 0.005 | 51019.0 | 30928.0 | 1.0 |
| ind[493] | -0.786 | 0.556 | -1.836 | 0.254 | 0.003 | 0.002 | 43233.0 | 28481.0 | 1.0 |
| ind[494] | -0.475 | 0.483 | -1.397 | 0.413 | 0.002 | 0.002 | 44335.0 | 29236.0 | 1.0 |
| ind[495] | 1.059 | 0.453 | 0.230 | 1.932 | 0.002 | 0.002 | 37272.0 | 28643.0 | 1.0 |
| ind[496] | 0.869 | 0.727 | -0.517 | 2.226 | 0.003 | 0.003 | 54498.0 | 28570.0 | 1.0 |
| ind[497] | 2.227 | 0.722 | 0.869 | 3.587 | 0.003 | 0.002 | 57597.0 | 26925.0 | 1.0 |
| ind[498] | -1.135 | 0.533 | -2.166 | -0.161 | 0.002 | 0.002 | 52581.0 | 27906.0 | 1.0 |
| ind[499] | -0.107 | 0.454 | -0.973 | 0.736 | 0.002 | 0.002 | 39353.0 | 28588.0 | 1.0 |
| ind[500] | 1.008 | 0.449 | 0.166 | 1.850 | 0.002 | 0.002 | 42624.0 | 28692.0 | 1.0 |
| ind[501] | 0.428 | 0.668 | -0.804 | 1.701 | 0.003 | 0.003 | 55199.0 | 28418.0 | 1.0 |
| ind[502] | 1.179 | 0.456 | 0.310 | 2.028 | 0.002 | 0.002 | 35201.0 | 29224.0 | 1.0 |
| ind[503] | -0.614 | 0.843 | -2.203 | 0.969 | 0.004 | 0.004 | 53802.0 | 29368.0 | 1.0 |
| ind[504] | 0.658 | 0.999 | -1.234 | 2.512 | 0.004 | 0.004 | 55687.0 | 30488.0 | 1.0 |
| ind[505] | -0.614 | 0.520 | -1.579 | 0.375 | 0.003 | 0.002 | 39691.0 | 28393.0 | 1.0 |
| ind[506] | -0.388 | 0.737 | -1.777 | 0.987 | 0.003 | 0.004 | 53111.0 | 28685.0 | 1.0 |
| ind[507] | 1.870 | 0.434 | 1.053 | 2.680 | 0.002 | 0.002 | 42033.0 | 28400.0 | 1.0 |
| ind[508] | 1.277 | 0.573 | 0.199 | 2.363 | 0.003 | 0.002 | 48506.0 | 28848.0 | 1.0 |
| ind[509] | -1.291 | 0.907 | -3.036 | 0.370 | 0.004 | 0.004 | 54197.0 | 26422.0 | 1.0 |
| ind[510] | -0.186 | 0.498 | -1.114 | 0.776 | 0.002 | 0.002 | 40799.0 | 29146.0 | 1.0 |
| ind[511] | -1.329 | 0.625 | -2.516 | -0.172 | 0.003 | 0.003 | 38899.0 | 27043.0 | 1.0 |
| ind[512] | 1.153 | 0.457 | 0.293 | 2.015 | 0.002 | 0.002 | 42267.0 | 28922.0 | 1.0 |
| ind[513] | 1.325 | 0.421 | 0.515 | 2.107 | 0.002 | 0.002 | 40684.0 | 28463.0 | 1.0 |
| ind[514] | 0.972 | 0.489 | 0.050 | 1.896 | 0.002 | 0.002 | 42294.0 | 26167.0 | 1.0 |
| ind[515] | -0.151 | 0.464 | -1.016 | 0.729 | 0.002 | 0.002 | 38208.0 | 28631.0 | 1.0 |
| ind[516] | -2.279 | 0.759 | -3.717 | -0.876 | 0.004 | 0.003 | 49031.0 | 27212.0 | 1.0 |
| ind[517] | 1.588 | 0.449 | 0.741 | 2.424 | 0.002 | 0.002 | 39775.0 | 28391.0 | 1.0 |
| ind[518] | 2.416 | 0.638 | 1.241 | 3.641 | 0.003 | 0.002 | 51671.0 | 28893.0 | 1.0 |
| ind[519] | -1.186 | 0.921 | -2.904 | 0.555 | 0.004 | 0.004 | 56494.0 | 29509.0 | 1.0 |
| ind[520] | -0.026 | 0.483 | -0.929 | 0.877 | 0.003 | 0.002 | 36266.0 | 29315.0 | 1.0 |
| ind[521] | -0.375 | 0.477 | -1.269 | 0.523 | 0.002 | 0.002 | 43381.0 | 29693.0 | 1.0 |
| ind[522] | 0.666 | 0.427 | -0.152 | 1.454 | 0.002 | 0.002 | 34928.0 | 27990.0 | 1.0 |
| ind[523] | 1.234 | 0.411 | 0.476 | 2.021 | 0.002 | 0.002 | 35702.0 | 28932.0 | 1.0 |
| ind[524] | -0.556 | 0.523 | -1.538 | 0.429 | 0.003 | 0.002 | 42060.0 | 28309.0 | 1.0 |
| ind[525] | -1.318 | 0.906 | -3.036 | 0.385 | 0.004 | 0.003 | 55990.0 | 28035.0 | 1.0 |
| ind[526] | -1.898 | 0.666 | -3.178 | -0.689 | 0.003 | 0.002 | 50020.0 | 28295.0 | 1.0 |
| ind[527] | 0.550 | 0.460 | -0.299 | 1.427 | 0.002 | 0.002 | 34103.0 | 29502.0 | 1.0 |
| ind[528] | 1.012 | 0.466 | 0.114 | 1.861 | 0.002 | 0.002 | 44812.0 | 28631.0 | 1.0 |
| ind[529] | 0.155 | 0.488 | -0.780 | 1.062 | 0.002 | 0.002 | 40554.0 | 27496.0 | 1.0 |
| ind[530] | 0.361 | 0.477 | -0.548 | 1.248 | 0.002 | 0.002 | 43018.0 | 29370.0 | 1.0 |
| ind[531] | 1.206 | 0.720 | -0.154 | 2.550 | 0.003 | 0.002 | 56962.0 | 28510.0 | 1.0 |
| ind[532] | -1.726 | 0.602 | -2.869 | -0.596 | 0.003 | 0.002 | 44788.0 | 28321.0 | 1.0 |
| ind[533] | 2.743 | 0.407 | 1.982 | 3.511 | 0.002 | 0.002 | 28934.0 | 28277.0 | 1.0 |
| ind[534] | 0.492 | 0.569 | -0.590 | 1.543 | 0.003 | 0.002 | 44222.0 | 26766.0 | 1.0 |
| ind[535] | -0.631 | 0.829 | -2.163 | 0.950 | 0.004 | 0.004 | 51564.0 | 26984.0 | 1.0 |
| ind[536] | -2.271 | 0.745 | -3.654 | -0.870 | 0.004 | 0.003 | 41969.0 | 26467.0 | 1.0 |
| ind[537] | 0.008 | 0.479 | -0.908 | 0.897 | 0.002 | 0.002 | 40764.0 | 29600.0 | 1.0 |
| ind[538] | 1.297 | 0.445 | 0.464 | 2.137 | 0.003 | 0.002 | 30463.0 | 27950.0 | 1.0 |
| ind[539] | 0.498 | 0.457 | -0.391 | 1.327 | 0.002 | 0.002 | 39462.0 | 27036.0 | 1.0 |
| ind[540] | 0.708 | 0.492 | -0.219 | 1.633 | 0.002 | 0.002 | 42072.0 | 29930.0 | 1.0 |
| ind[541] | -1.961 | 0.662 | -3.205 | -0.721 | 0.003 | 0.002 | 47562.0 | 26620.0 | 1.0 |
| ind[542] | 0.932 | 0.447 | 0.099 | 1.788 | 0.002 | 0.002 | 40434.0 | 27760.0 | 1.0 |
| ind[543] | -1.401 | 0.638 | -2.615 | -0.220 | 0.003 | 0.002 | 45634.0 | 25921.0 | 1.0 |
| ind[544] | -0.581 | 0.551 | -1.647 | 0.425 | 0.003 | 0.002 | 45779.0 | 28549.0 | 1.0 |
| ind[545] | -0.523 | 0.529 | -1.513 | 0.471 | 0.002 | 0.002 | 46996.0 | 27317.0 | 1.0 |
| ind[546] | 2.527 | 0.468 | 1.649 | 3.404 | 0.002 | 0.002 | 36366.0 | 27721.0 | 1.0 |
| ind[547] | -1.297 | 0.907 | -3.028 | 0.369 | 0.004 | 0.004 | 53884.0 | 26503.0 | 1.0 |
| ind[548] | 0.920 | 0.806 | -0.601 | 2.435 | 0.004 | 0.003 | 47632.0 | 28974.0 | 1.0 |
| ind[549] | 0.472 | 0.481 | -0.403 | 1.403 | 0.002 | 0.002 | 42628.0 | 28498.0 | 1.0 |
| ind[550] | 0.253 | 0.491 | -0.666 | 1.182 | 0.003 | 0.002 | 36640.0 | 29165.0 | 1.0 |
| ind[551] | -0.630 | 0.824 | -2.139 | 0.969 | 0.004 | 0.004 | 46192.0 | 27467.0 | 1.0 |
| ind[552] | -0.533 | 0.474 | -1.458 | 0.316 | 0.002 | 0.002 | 36923.0 | 29024.0 | 1.0 |
| ind[553] | -1.538 | 0.625 | -2.716 | -0.372 | 0.003 | 0.002 | 47778.0 | 29521.0 | 1.0 |
| ind[554] | -1.229 | 0.532 | -2.246 | -0.259 | 0.003 | 0.002 | 42292.0 | 28867.0 | 1.0 |
| ind[555] | -0.654 | 0.804 | -2.181 | 0.818 | 0.003 | 0.003 | 58927.0 | 29564.0 | 1.0 |
| ind[556] | 0.339 | 0.469 | -0.527 | 1.235 | 0.002 | 0.002 | 37255.0 | 29339.0 | 1.0 |
| ind[557] | 0.630 | 0.786 | -0.873 | 2.091 | 0.004 | 0.003 | 49292.0 | 29361.0 | 1.0 |
| ind[558] | -2.156 | 0.757 | -3.572 | -0.729 | 0.003 | 0.003 | 62256.0 | 26134.0 | 1.0 |
| ind[559] | 0.590 | 0.726 | -0.795 | 1.944 | 0.003 | 0.003 | 56958.0 | 27498.0 | 1.0 |
| ind[560] | -0.102 | 0.763 | -1.535 | 1.335 | 0.003 | 0.004 | 57518.0 | 27960.0 | 1.0 |
| ind[561] | -0.072 | 0.500 | -1.017 | 0.859 | 0.003 | 0.003 | 36554.0 | 27977.0 | 1.0 |
| ind[562] | 2.278 | 0.491 | 1.393 | 3.242 | 0.003 | 0.002 | 38643.0 | 28421.0 | 1.0 |
| ind[563] | 0.455 | 0.471 | -0.425 | 1.345 | 0.002 | 0.002 | 40974.0 | 28656.0 | 1.0 |
| ind[564] | -0.949 | 0.584 | -2.062 | 0.135 | 0.003 | 0.002 | 53436.0 | 28366.0 | 1.0 |
| ind[565] | -1.743 | 0.853 | -3.403 | -0.203 | 0.004 | 0.003 | 58453.0 | 28206.0 | 1.0 |
| ind[566] | 0.919 | 0.495 | 0.005 | 1.871 | 0.003 | 0.002 | 37374.0 | 29309.0 | 1.0 |
| ind[567] | 0.339 | 0.527 | -0.667 | 1.318 | 0.003 | 0.002 | 42838.0 | 28654.0 | 1.0 |
| ind[568] | 0.784 | 0.449 | -0.104 | 1.597 | 0.002 | 0.002 | 42651.0 | 28109.0 | 1.0 |
| ind[569] | -0.309 | 0.529 | -1.315 | 0.675 | 0.002 | 0.003 | 47542.0 | 27133.0 | 1.0 |
| ind[570] | 0.988 | 0.690 | -0.318 | 2.261 | 0.003 | 0.002 | 49755.0 | 29761.0 | 1.0 |
| ind[571] | -2.044 | 0.663 | -3.332 | -0.846 | 0.003 | 0.002 | 51859.0 | 27133.0 | 1.0 |
| ind[572] | 0.991 | 0.459 | 0.097 | 1.827 | 0.002 | 0.002 | 38963.0 | 29238.0 | 1.0 |
| ind[573] | 1.110 | 0.445 | 0.263 | 1.937 | 0.002 | 0.002 | 32623.0 | 29432.0 | 1.0 |
| ind[574] | 1.218 | 0.634 | 0.032 | 2.408 | 0.003 | 0.002 | 44524.0 | 28457.0 | 1.0 |
| ind[575] | -1.737 | 0.673 | -3.036 | -0.522 | 0.003 | 0.002 | 55087.0 | 29020.0 | 1.0 |
| ind[576] | 0.678 | 0.441 | -0.180 | 1.479 | 0.002 | 0.002 | 37830.0 | 28757.0 | 1.0 |
| ind[577] | 1.596 | 0.440 | 0.764 | 2.419 | 0.002 | 0.002 | 43183.0 | 29053.0 | 1.0 |
| ind[578] | 0.449 | 0.719 | -0.938 | 1.777 | 0.003 | 0.003 | 53401.0 | 29110.0 | 1.0 |
| ind[579] | 0.914 | 0.703 | -0.397 | 2.255 | 0.003 | 0.003 | 52493.0 | 27411.0 | 1.0 |
| ind[580] | -0.658 | 0.821 | -2.171 | 0.901 | 0.004 | 0.004 | 54284.0 | 27321.0 | 1.0 |
| ind[581] | 0.807 | 0.712 | -0.551 | 2.136 | 0.003 | 0.003 | 54786.0 | 27423.0 | 1.0 |
| ind[582] | 0.808 | 0.458 | -0.050 | 1.661 | 0.002 | 0.002 | 35353.0 | 28361.0 | 1.0 |
| ind[583] | -1.029 | 0.582 | -2.122 | 0.063 | 0.003 | 0.002 | 47404.0 | 28871.0 | 1.0 |
| ind[584] | -0.554 | 0.555 | -1.604 | 0.484 | 0.003 | 0.002 | 42458.0 | 26678.0 | 1.0 |
| ind[585] | -0.724 | 0.553 | -1.749 | 0.323 | 0.002 | 0.002 | 56909.0 | 27752.0 | 1.0 |
| ind[586] | -2.296 | 0.740 | -3.701 | -0.937 | 0.003 | 0.003 | 58011.0 | 27166.0 | 1.0 |
| ind[587] | -1.234 | 0.919 | -2.958 | 0.476 | 0.004 | 0.004 | 55493.0 | 28689.0 | 1.0 |
| ind[588] | 0.397 | 0.713 | -0.957 | 1.728 | 0.003 | 0.003 | 51892.0 | 29121.0 | 1.0 |
| ind[589] | 1.019 | 0.449 | 0.156 | 1.847 | 0.002 | 0.002 | 38903.0 | 28420.0 | 1.0 |
| ind[590] | 0.627 | 0.496 | -0.313 | 1.548 | 0.002 | 0.002 | 39530.0 | 27620.0 | 1.0 |
| ind[591] | 0.326 | 0.793 | -1.170 | 1.818 | 0.003 | 0.004 | 54216.0 | 27210.0 | 1.0 |
| ind[592] | -0.864 | 0.793 | -2.397 | 0.577 | 0.004 | 0.003 | 49409.0 | 29347.0 | 1.0 |
| ind[593] | 0.168 | 0.480 | -0.744 | 1.061 | 0.002 | 0.002 | 37846.0 | 28075.0 | 1.0 |
| ind[594] | -0.791 | 1.062 | -2.826 | 1.169 | 0.005 | 0.005 | 47896.0 | 30582.0 | 1.0 |
| ind[595] | 1.968 | 0.447 | 1.114 | 2.790 | 0.002 | 0.002 | 37211.0 | 29942.0 | 1.0 |
| ind[596] | -2.337 | 0.743 | -3.739 | -0.975 | 0.003 | 0.003 | 51363.0 | 28053.0 | 1.0 |
| ind[597] | -0.362 | 0.526 | -1.330 | 0.642 | 0.002 | 0.002 | 44800.0 | 29284.0 | 1.0 |
| ind[598] | -0.763 | 0.484 | -1.676 | 0.143 | 0.002 | 0.002 | 44845.0 | 28331.0 | 1.0 |
| ind[599] | -1.353 | 0.623 | -2.516 | -0.177 | 0.003 | 0.002 | 48213.0 | 26724.0 | 1.0 |
| ind[600] | 0.150 | 0.487 | -0.800 | 1.032 | 0.003 | 0.002 | 37716.0 | 28365.0 | 1.0 |
| ind[601] | 0.070 | 0.792 | -1.406 | 1.559 | 0.003 | 0.004 | 54828.0 | 30191.0 | 1.0 |
| ind[602] | -0.244 | 0.833 | -1.854 | 1.269 | 0.004 | 0.004 | 56943.0 | 28122.0 | 1.0 |
| ind[603] | 0.203 | 0.488 | -0.714 | 1.121 | 0.003 | 0.002 | 35674.0 | 28562.0 | 1.0 |
| ind[604] | -1.516 | 0.682 | -2.808 | -0.250 | 0.003 | 0.003 | 46250.0 | 29117.0 | 1.0 |
| ind[605] | -1.014 | 0.586 | -2.113 | 0.079 | 0.003 | 0.002 | 45672.0 | 27813.0 | 1.0 |
| ind[606] | -1.533 | 0.884 | -3.238 | 0.078 | 0.004 | 0.003 | 55303.0 | 28967.0 | 1.0 |
| ind[607] | -0.828 | 0.546 | -1.866 | 0.179 | 0.003 | 0.002 | 45165.0 | 28196.0 | 1.0 |
| ind[608] | 0.625 | 0.408 | -0.134 | 1.394 | 0.002 | 0.002 | 36765.0 | 28559.0 | 1.0 |
| ind[609] | 2.074 | 0.707 | 0.765 | 3.421 | 0.003 | 0.002 | 54112.0 | 29156.0 | 1.0 |
| ind[610] | -0.453 | 0.732 | -1.858 | 0.884 | 0.003 | 0.003 | 50949.0 | 29475.0 | 1.0 |
| ind[611] | -1.182 | 0.925 | -2.982 | 0.515 | 0.004 | 0.004 | 52727.0 | 28649.0 | 1.0 |
| ind[612] | -1.604 | 0.609 | -2.756 | -0.473 | 0.003 | 0.002 | 51724.0 | 27406.0 | 1.0 |
| ind[613] | 2.063 | 0.662 | 0.849 | 3.328 | 0.003 | 0.002 | 41727.0 | 30139.0 | 1.0 |
| ind[614] | -1.511 | 0.887 | -3.165 | 0.190 | 0.004 | 0.003 | 55181.0 | 29453.0 | 1.0 |
| ind[615] | 1.289 | 0.441 | 0.484 | 2.131 | 0.002 | 0.002 | 42711.0 | 27761.0 | 1.0 |
| ind[616] | -1.060 | 0.582 | -2.144 | 0.048 | 0.003 | 0.002 | 39373.0 | 27107.0 | 1.0 |
| ind[617] | 0.378 | 0.483 | -0.558 | 1.259 | 0.002 | 0.002 | 45888.0 | 28437.0 | 1.0 |
| ind[618] | -0.144 | 0.756 | -1.582 | 1.238 | 0.003 | 0.004 | 50015.0 | 28764.0 | 1.0 |
| ind[619] | 1.981 | 0.447 | 1.118 | 2.800 | 0.002 | 0.002 | 41880.0 | 28413.0 | 1.0 |
| ind[620] | -1.470 | 0.617 | -2.619 | -0.300 | 0.003 | 0.002 | 44681.0 | 26675.0 | 1.0 |
| ind[621] | 2.034 | 0.521 | 1.046 | 2.994 | 0.003 | 0.002 | 42327.0 | 30342.0 | 1.0 |
| ind[622] | 0.408 | 0.539 | -0.570 | 1.467 | 0.003 | 0.002 | 44685.0 | 28470.0 | 1.0 |
| ind[623] | 0.095 | 0.476 | -0.804 | 0.982 | 0.003 | 0.002 | 33731.0 | 28441.0 | 1.0 |
| ind[624] | -1.051 | 0.585 | -2.173 | 0.022 | 0.003 | 0.002 | 45360.0 | 28932.0 | 1.0 |
| ind[625] | 3.315 | 0.698 | 1.988 | 4.611 | 0.003 | 0.002 | 52363.0 | 28003.0 | 1.0 |
| ind[626] | 1.576 | 0.497 | 0.647 | 2.521 | 0.002 | 0.002 | 40421.0 | 27290.0 | 1.0 |
| ind[627] | -0.827 | 0.577 | -1.884 | 0.283 | 0.003 | 0.002 | 47686.0 | 28081.0 | 1.0 |
| ind[628] | 1.623 | 0.766 | 0.187 | 3.056 | 0.003 | 0.002 | 57605.0 | 28374.0 | 1.0 |
| ind[629] | -0.394 | 0.466 | -1.298 | 0.458 | 0.002 | 0.002 | 35916.0 | 29399.0 | 1.0 |
| ind[630] | -0.205 | 0.486 | -1.124 | 0.702 | 0.002 | 0.002 | 45005.0 | 28870.0 | 1.0 |
| ind[631] | -0.418 | 0.879 | -2.070 | 1.232 | 0.004 | 0.004 | 57606.0 | 29289.0 | 1.0 |
| ind[632] | -0.705 | 0.815 | -2.257 | 0.822 | 0.003 | 0.004 | 56792.0 | 28448.0 | 1.0 |
| ind[633] | -1.838 | 0.800 | -3.361 | -0.360 | 0.004 | 0.003 | 47701.0 | 27772.0 | 1.0 |
| ind[634] | 0.027 | 0.518 | -0.984 | 0.966 | 0.002 | 0.003 | 45994.0 | 26825.0 | 1.0 |
| ind[635] | -0.659 | 0.819 | -2.166 | 0.928 | 0.004 | 0.004 | 52303.0 | 27581.0 | 1.0 |
| ind[637] | -1.022 | 0.582 | -2.123 | 0.053 | 0.003 | 0.002 | 51382.0 | 26824.0 | 1.0 |
| ind[638] | 1.236 | 0.478 | 0.309 | 2.113 | 0.002 | 0.002 | 45673.0 | 28517.0 | 1.0 |
| ind[639] | -1.194 | 0.541 | -2.241 | -0.198 | 0.003 | 0.002 | 44713.0 | 28945.0 | 1.0 |
| ind[640] | -1.176 | 0.923 | -2.910 | 0.551 | 0.004 | 0.004 | 55900.0 | 29192.0 | 1.0 |
| ind[641] | -0.078 | 0.862 | -1.692 | 1.526 | 0.004 | 0.005 | 51502.0 | 28313.0 | 1.0 |
| ind[642] | 0.744 | 0.477 | -0.145 | 1.648 | 0.002 | 0.002 | 42774.0 | 29026.0 | 1.0 |
| ind[643] | 0.358 | 0.473 | -0.561 | 1.218 | 0.002 | 0.002 | 41955.0 | 29395.0 | 1.0 |
| ind[644] | 0.102 | 0.537 | -0.904 | 1.104 | 0.002 | 0.003 | 52399.0 | 25735.0 | 1.0 |
| ind[645] | 0.242 | 0.468 | -0.656 | 1.101 | 0.002 | 0.002 | 37001.0 | 28660.0 | 1.0 |
| ind[646] | 0.123 | 0.473 | -0.764 | 1.007 | 0.003 | 0.002 | 31962.0 | 27011.0 | 1.0 |
| ind[647] | -0.141 | 0.490 | -1.066 | 0.776 | 0.002 | 0.003 | 45834.0 | 29069.0 | 1.0 |
| ind[648] | -0.660 | 0.818 | -2.199 | 0.861 | 0.004 | 0.004 | 49867.0 | 28319.0 | 1.0 |
| ind[649] | -0.040 | 0.749 | -1.462 | 1.358 | 0.003 | 0.004 | 56525.0 | 26778.0 | 1.0 |
| ind[650] | 1.716 | 0.462 | 0.847 | 2.587 | 0.002 | 0.002 | 37126.0 | 28995.0 | 1.0 |
| ind[651] | -0.658 | 0.825 | -2.220 | 0.856 | 0.003 | 0.004 | 59284.0 | 28636.0 | 1.0 |
| ind[652] | -1.550 | 0.866 | -3.231 | 0.007 | 0.004 | 0.003 | 56152.0 | 30478.0 | 1.0 |
| ind[653] | 1.344 | 0.676 | 0.050 | 2.590 | 0.003 | 0.002 | 49301.0 | 28049.0 | 1.0 |
| ind[654] | -0.002 | 1.219 | -2.336 | 2.255 | 0.005 | 0.006 | 51240.0 | 30540.0 | 1.0 |
| ind[655] | 1.204 | 0.423 | 0.398 | 1.994 | 0.002 | 0.002 | 37280.0 | 25372.0 | 1.0 |
| ind[656] | -1.990 | 0.660 | -3.286 | -0.815 | 0.003 | 0.002 | 44652.0 | 27755.0 | 1.0 |
| ind[657] | 0.511 | 0.733 | -0.899 | 1.848 | 0.003 | 0.003 | 57670.0 | 28889.0 | 1.0 |
| ind[658] | 1.141 | 0.524 | 0.156 | 2.121 | 0.002 | 0.002 | 53065.0 | 29869.0 | 1.0 |
| ind[659] | -0.760 | 0.557 | -1.817 | 0.263 | 0.003 | 0.002 | 49550.0 | 25467.0 | 1.0 |
| ind[660] | -0.278 | 0.526 | -1.265 | 0.709 | 0.003 | 0.002 | 44043.0 | 29136.0 | 1.0 |
| ind[661] | -0.922 | 0.806 | -2.427 | 0.601 | 0.004 | 0.003 | 51077.0 | 29888.0 | 1.0 |
| ind[662] | -0.132 | 0.530 | -1.142 | 0.851 | 0.003 | 0.003 | 42020.0 | 28269.0 | 1.0 |
| ind[663] | -0.034 | 0.511 | -0.993 | 0.922 | 0.002 | 0.003 | 42166.0 | 28312.0 | 1.0 |
| ind[664] | -0.214 | 0.539 | -1.225 | 0.805 | 0.003 | 0.003 | 45723.0 | 29220.0 | 1.0 |
| ind[665] | 1.583 | 0.540 | 0.561 | 2.584 | 0.003 | 0.002 | 36122.0 | 28740.0 | 1.0 |
| ind[666] | -1.211 | 0.634 | -2.399 | -0.027 | 0.003 | 0.002 | 46795.0 | 26978.0 | 1.0 |
| ind[667] | -0.980 | 0.534 | -1.996 | 0.012 | 0.003 | 0.002 | 40674.0 | 28846.0 | 1.0 |
| ind[668] | 0.190 | 0.449 | -0.654 | 1.032 | 0.002 | 0.002 | 34864.0 | 28955.0 | 1.0 |
| ind[669] | -1.257 | 0.645 | -2.499 | -0.055 | 0.003 | 0.002 | 43470.0 | 28312.0 | 1.0 |
| ind[670] | -0.176 | 0.483 | -1.083 | 0.733 | 0.003 | 0.002 | 37301.0 | 29173.0 | 1.0 |
| ind[671] | 0.161 | 0.580 | -0.938 | 1.248 | 0.003 | 0.003 | 43508.0 | 28913.0 | 1.0 |
| ind[672] | 0.212 | 0.514 | -0.756 | 1.181 | 0.002 | 0.003 | 46285.0 | 28294.0 | 1.0 |
| ind[673] | -0.452 | 0.497 | -1.410 | 0.455 | 0.002 | 0.002 | 40766.0 | 26876.0 | 1.0 |
| ind[674] | -0.900 | 0.545 | -1.950 | 0.099 | 0.003 | 0.002 | 47083.0 | 26518.0 | 1.0 |
| ind[675] | -0.650 | 0.816 | -2.205 | 0.850 | 0.004 | 0.004 | 50565.0 | 27811.0 | 1.0 |
| ind[676] | -0.347 | 0.472 | -1.234 | 0.540 | 0.002 | 0.002 | 39803.0 | 28301.0 | 1.0 |
| ind[677] | -1.315 | 0.902 | -2.999 | 0.361 | 0.004 | 0.004 | 47063.0 | 28329.0 | 1.0 |
| ind[678] | -0.508 | 0.506 | -1.487 | 0.414 | 0.003 | 0.002 | 37600.0 | 27931.0 | 1.0 |
| ind[679] | 1.530 | 0.430 | 0.701 | 2.317 | 0.002 | 0.001 | 43752.0 | 28727.0 | 1.0 |
| ind[680] | -1.189 | 0.926 | -2.920 | 0.541 | 0.004 | 0.004 | 47660.0 | 27745.0 | 1.0 |
| ind[681] | 0.141 | 0.440 | -0.711 | 0.945 | 0.002 | 0.002 | 42205.0 | 28259.0 | 1.0 |
| ind[682] | -1.262 | 0.638 | -2.499 | -0.100 | 0.003 | 0.002 | 44439.0 | 27477.0 | 1.0 |
| ind[683] | 0.424 | 0.447 | -0.419 | 1.255 | 0.002 | 0.002 | 37081.0 | 29446.0 | 1.0 |
| ind[684] | 0.364 | 0.516 | -0.605 | 1.334 | 0.003 | 0.002 | 35591.0 | 27918.0 | 1.0 |
| ind[685] | 2.043 | 0.392 | 1.326 | 2.801 | 0.002 | 0.001 | 35532.0 | 29265.0 | 1.0 |
| ind[686] | -0.356 | 0.522 | -1.341 | 0.628 | 0.002 | 0.002 | 53076.0 | 27283.0 | 1.0 |
| ind[687] | 0.561 | 0.446 | -0.269 | 1.407 | 0.002 | 0.002 | 37331.0 | 27564.0 | 1.0 |
| ind[688] | 0.220 | 0.670 | -1.030 | 1.480 | 0.003 | 0.004 | 63937.0 | 28263.0 | 1.0 |
| ind[690] | -0.310 | 0.750 | -1.737 | 1.081 | 0.003 | 0.004 | 51787.0 | 28691.0 | 1.0 |
| ind[691] | -0.845 | 0.683 | -2.129 | 0.440 | 0.003 | 0.003 | 47570.0 | 26995.0 | 1.0 |
| ind[692] | -0.217 | 0.688 | -1.510 | 1.072 | 0.003 | 0.003 | 54035.0 | 29562.0 | 1.0 |
| ind[693] | -0.676 | 0.569 | -1.782 | 0.356 | 0.003 | 0.002 | 42989.0 | 28140.0 | 1.0 |
| ind[694] | -1.526 | 0.894 | -3.268 | 0.089 | 0.004 | 0.003 | 44347.0 | 29057.0 | 1.0 |
| ind[695] | -0.508 | 0.876 | -2.141 | 1.170 | 0.003 | 0.004 | 63325.0 | 28317.0 | 1.0 |
| ind[696] | -2.014 | 0.788 | -3.537 | -0.585 | 0.004 | 0.003 | 44450.0 | 27379.0 | 1.0 |
| ind[697] | 0.493 | 0.789 | -0.967 | 1.974 | 0.003 | 0.004 | 53572.0 | 27474.0 | 1.0 |
| ind[698] | 1.234 | 0.576 | 0.125 | 2.303 | 0.002 | 0.002 | 53338.0 | 28014.0 | 1.0 |

## sample stats¶

In [21]:

```
#R-hat (we used 4 chains) and effective sample size

az.summary(trace, var_names = ['gender','field','seniority', 's_ind','s_field', 's_seniority'])[['r_hat', 'ess_bulk']].T
```

Out[21]:

|  | gender[female] | gender[male] | field[Arts & Hum] | field[Life & Biomed] | field[Natural Science] | field[Social Science] | field[Tech & Engineering] | seniority[0-10] | seniority[11-20] | seniority[21-30] | seniority[31-40] | seniority[>40] | s\_ind | s\_field | s\_seniority |
| --- | --- | --- | --- | --- | --- | --- | --- | --- | --- | --- | --- | --- | --- | --- | --- |
| r\_hat | 1.0 | 1.0 | 1.0 | 1.0 | 1.0 | 1.0 | 1.0 | 1.0 | 1.0 | 1.0 | 1.0 | 1.0 | 1.0 | 1.0 | 1.0 |
| ess\_bulk | 6469.0 | 6813.0 | 8377.0 | 9124.0 | 9002.0 | 8659.0 | 9655.0 | 18738.0 | 11148.0 | 14247.0 | 14269.0 | 17558.0 | 10968.0 | 7303.0 | 6296.0 |

In [22]:

```
#see here: https://docs.pymc.io/en/v3/pymc-examples/examples/diagnostics_and_criticism/sampler-stats.html

#print number of divergences, ideally 0
print(f'divergences: {trace.sample_stats["diverging"].values.sum()}')

#print the acceptance rate
print(f'mean acceptance rate: {trace.sample_stats["acceptance_rate"].values.mean()}')

#compare the overall distribution of the energy levels with the change of energy between successive samples. Ideally, they should be very similar
az.plot_energy(trace, figsize=(6, 4));
```

```
divergences: 0
mean acceptance rate: 0.9879681348339189
```

## Results¶

In [23]:

```
# plot difference between coefficients for female and male

fig, ax = plt.subplots(figsize = (5,2))

male = trace.posterior['gender'].loc[:,:,'male']
female = trace.posterior['gender'].loc[:,:,'female']

az.plot_posterior(female-male, ax=ax)

plt.show()
```

In [24]:

```
# for table 3 in the paper: get the description of the distribution of the difference

az.summary(female-male)
```

Out[24]:

|  | mean | sd | hdi\_3% | hdi\_97% | mcse\_mean | mcse\_sd | ess\_bulk | ess\_tail | r\_hat |
| --- | --- | --- | --- | --- | --- | --- | --- | --- | --- |
| gender | 0.03 | 0.108 | -0.172 | 0.234 | 0.001 | 0.001 | 9565.0 | 16848.0 | 1.0 |

In [25]:

```
# generate posterior predictive samples
# we generate them for the entire sample set to male (other characteristics intact) and female (idem)

ppcs = {}
with gender_hyp1:
    for i in range(2):
        pm.set_data({"G": np.repeat(i, len(df))})
        thinned_trace = trace.sel(draw=slice(None, None, 5))
        ppc = pm.sample_posterior_predictive(thinned_trace, progressbar = False)
        ppcs[i] = ppc
```

```
Sampling: [y]
Sampling: [y]
```

In [26]:

```
#posterior predictive counts of the various qrp response options

# randomly draw 20k samples from the ones  generated


female = np.random.choice(np.ravel(ppcs[0].posterior_predictive['y'].values),
                          size = 20000,
                          replace=True)
male = np.random.choice(np.ravel(ppcs[1].posterior_predictive['y'].values),
                        size = 20000,
                        replace=True)

ppcdf = pd.DataFrame([female, male], index = ['female', 'male']).T
data = ppcdf.stack().reset_index().rename(columns = {0:'QRP', 'level_1':'Gender'})

# save data to make figures for paper in other notebook

with open('...', 'wb') as handle:
    pickle.dump(data, handle, protocol=pickle.HIGHEST_PROTOCOL)

sns.countplot(data=data,
              x = 'QRP',
              hue = 'Gender')

plt.show()
```

In [47]:

```
# plot the results for the standard deviation hyper parameters
# this gives an idea of how much variation there is between fields, between seniority cats, between individuals, and between questions

fig, ax = plt.subplots(figsize = (7,5))

az.plot_forest(trace,
               var_names = ['s_field','s_seniority','s_ind'],
               kind = 'ridgeplot',
               ridgeplot_alpha = 0.5,
               combined = True,
               ridgeplot_truncate = True,
               ridgeplot_overlap = 1,
               ax=ax)
ax.set_yticklabels(['Respondent', 'Seniority','Field'])
fig.tight_layout()
plt.savefig('...', dpi = 300)
```

In [35]:

```
# dct for question labels

q_dct = ({'C1':'field','C2': 'Continent','C3':'Seniority','C4': 'gender','C5':'role',
          'C6': 'number of applications','C7':'observed qrp','C8':'alterted qrp',
          
          'A1':'confidence in deliverables',
          'A2': 'overstated confidence in app',
          'A3':'proposals with missing authors',
          'A4': 'hired consultants',
          'A5': 'irrelevant outputs in report',
          'A6': 'applied for too much',
          'A7': 'new equipment instead of maintenance',
          'A8': 'improper use funds',
          'A9': 'improper use funds at end',
          'A10':'overlapping grants',
          'A11':'research already done',
          'A12': 'applied for cv',
          'A13': 'applied for employing someone',
          'A14': 'selective citing',
          'A15': 'reviewer selection',
          'A16': 'accurate grant reports',
          'A17': 'inexpert reviewers',
          'A18': 'unfair report negative',
          'A19': 'unfair report positive',
          'A20': 'success rate',
          
         'R1': 'confidence in reviews',
        'R2':'review behavior when uncertain',
          'R3': 'likelihood good reviewer',
         'R4': 'insufficient effort in review',
         'R5': 'not read complete file',
         'R6': 'review outside expertise',
         'R7': 'review of friends',
         'R8': 'bias in reviews',
         
         'CoI':'CoI',
          
         'P1': 'instructions integrity from funders',
         'P2': 'training integrity from funders',
         'P4':'ill prepared panelist (self)',
         'P5': 'ill prepared panelist (others)',
         'P3': 'incomparable proposals',
         'P6': 'no quality differences'})
```

In [36]:

```
# plot the cutpoint estimates, comparing the same cutpoint for different questions
# this shows how much difference there is between questions

fig, axs = plt.subplots(2,3, figsize = (20,15), sharey = True)

for ax,i in zip(axs.flat, cutpoint_codes):

    az.plot_forest(trace, var_names = ['cutpoints'],combined = True, coords = {'c_n':i}, ax=ax)
    ax.set_title(i, fontsize = 23)
    ax.set_yticklabels([q_dct[i] for i in q_codes], fontsize = 18)

plt.suptitle('Cutpoint coefficients for all QRP questions (gender hypothesis test 1)', fontsize = 30)
fig.tight_layout()
plt.savefig('...', dpi = 300)
```

In [ ]:

```

```
