# Supplementary material for: Questionable research practices in competitive grant funding: A survey
Source: PLoS One. 2023 Nov 2;18(11):e0293310. doi: 10.1371/journal.pone.0293310 (PMC10621923; doi:10.1371/journal.pone.0293310)
Supplement: S3 File — The code can also be accessed through the OSF page of the project (https://osf.io/jk6wd/). (ZIP) [file pone.0293310.s015.zip › S13 File/ERF_gender_hypothesis2_upload.html]

ERF\_gender\_hypothesis2\_upload


In [1]:

```
import pandas as pd
import numpy as np
import pickle

# for the stats
import pymc as pm
import bambi as bmb
from scipy.special import expit, logit


# for plotting
import seaborn as sns
import matplotlib.pyplot as plt
import arviz as az
import pickle
# for the DAG

import collections.abc
#causalgraphicalmodels needs the four following aliases to be done manually.
collections.Iterable = collections.abc.Iterable
collections.Mapping = collections.abc.Mapping
collections.MutableSet = collections.abc.MutableSet
collections.MutableMapping = collections.abc.MutableMapping
from causalgraphicalmodels import CausalGraphicalModel
import daft
```

In [2]:

```
# versions

print('\n'.join(f'{m.__name__}=={m.__version__}' for m in globals().values() if getattr(m, '__version__', None)))
```

```
pandas==1.5.3
numpy==1.24.2
pymc==5.1.2
bambi==0.10.0
seaborn==0.12.2
arviz==0.15.1
daft==0.1.2
```

In [3]:

```
sns.set_palette("Dark2")
sns.set_style("darkgrid")
sns.set_context("paper", font_scale=1.5)
```

In [4]:

```
# seed

SEED = 2808

np.random.seed(SEED)
```

# Hypothesis¶

## Description¶

**GENDER HYPOTHESIS 2: Respondents that identify as male are more likely to have at least one QRP with a score of 4 or higher.**

## Reporting¶

In the preregistered analysis code, we had written that we would report model 1 in the paper. The model reported here is very similar (same variables and data included), but adds hierarchical structure in the shape of hyperpriors for field and seniority. We do this because some of the gender x field x seniority cells are empty or have very few responses. The multi-level structure allows the model to inform field/seniority groups with a lot of data to inform those with little data. Adding the hierarchical structure did not affect the results substantially. The preregistered model is available on the OSF page of the project.

## Justification¶

We expected higher qrp levels for those who identify as male on the basis of:

- the pilot study (see results below, generally a weak effect of 'male' increasing the probability of higher ordinal responses.
- existing research: Fang et al. 2013 doi: 10.1128/mBio.00640-12 find that in reports of scientific misconduct (fraud, fabrication, and plagiarism) the proportion of males is higher than the proportion of males in the relevant disciplines. In line with this, Gopalakrishna et al 2022 doi: 10.1371/journal.pone.0263023 find that being a male increased the odds of various qrps. While one other study did not find this in an analysis of 120 cases of wrongdoing (of which 40 FFP and 40 other research misconduct), we think the evidence in favour of this hypothesis is stronger, in particular due to the much larger sample size and scope of the Gopalakrishna study, and the fact that the results are weakly confirmed by our pilot study.
- We choose to compare 'male' with 'female' instead of 'non-male' (which includes nonbinary and other) because potential explanations involve societal roles of men/women and levels of testosterone. These explanations are easier to evaluate if the hypothesis focuses on men and women. In the exploratory analysis, we will rerun the same analysis with male vs non-male, and with all gender categories.
- field and career stage are included in the DAG because previous research suggests they might play a role. We did not include 'continent' because we have no reason (neither pilot results nor previous research) to think it is relevant. In addition, our samples will be mostly european, meaning that we'll have too little data from other continents for meaningfull comparison.

# DAG¶

In [5]:

```
# make our causal assumptions explicit

dag_gender = CausalGraphicalModel(nodes=["QRP", "G", "Age",'Field'], 
                              edges=[("Age", "QRP"), ("Age", "QRP"), ("Field", "QRP"), 
                                     ('G','Age'),('G','QRP'),('G','Field'), ('Age','Field'),
                                    ])
dag_gender.draw()
```

Out[5]:

Field

Field


QRP

QRP


Field->QRP


Age

Age


Age->Field


Age->QRP


G

G


G->Field


G->QRP


G->Age

In [6]:

```
# a function to identify backdoor paths, and check for gender (our variable of interest)


def backdoor(dag, predictor, outcome):
    all_adjustment_sets = dag.get_all_backdoor_adjustment_sets(predictor, outcome)
    for s in all_adjustment_sets:
        if all(not t.issubset(s) for t in all_adjustment_sets if t != s):
            if s != {"U"}:
                print(s)

                
# For gender

backdoor(dag_gender,'G','QRP')
```

```
frozenset()
```

note: We are interested in the direct effect of gender. Hence, we will include seniority and field as controls and gender variable of interest. This model will give us an estimate of the direct effect of gender.

# Indices & Data¶

In [7]:

```
# data has been processed already in ERF_get_data.
# Now make it into the right shape for this test.

df = pd.read_csv('...')

r_qrp = ['R4', 'R5', 'R6','R7','R8']
r_qrp = ['R4', 'R5', 'R6','R7','R8']
a_qrp = ['A2','A3','A5', 'A6','A8', 'A9', 'A10', 'A11','A14','A15']
demo = ['C1', 'C2', 'C3', 'C4']


# select the columns we need

df_bin = df[a_qrp + r_qrp + ['C1','C3','C4']].copy()

# drop other genders and nans

other_genders = len((df_bin.loc[(df_bin.C4 != 'male') & (df_bin.C4 != 'female')]))
df_bin = df_bin.loc[(df_bin.C4 == 'male') | (df_bin.C4 == 'female')]

predrop = len(df_bin)
df_bin = df_bin.dropna()
postdrop = len(df_bin)

print(f'number of non-male/female responses removed: {other_genders}')
print(f'rows with nans removed: {predrop - postdrop}')
print(f'total responses: {postdrop}')

# FREQ indicator

df_bin['FREQ'] = np.where((df_bin[a_qrp + r_qrp] > 3).any(axis=1),1,0)

# rename demo columns

df_bin.rename(columns = {'C1':'field','C3':'seniority','C4':'gender'},inplace=True)

# set dtype to categorical

df_bin[['field','seniority','gender']] = df_bin[['field','seniority','gender']].astype('category')
df_bin.tail()

df_bin.head()
```

```
number of non-male/female responses removed: 21
rows with nans removed: 187
total responses: 496
```

Out[7]:

|  | A2 | A3 | A5 | A6 | A8 | A9 | A10 | A11 | A14 | A15 | R4 | R5 | R6 | R7 | R8 | field | seniority | gender | FREQ |
| --- | --- | --- | --- | --- | --- | --- | --- | --- | --- | --- | --- | --- | --- | --- | --- | --- | --- | --- | --- |
| 1 | 7.0 | 1.0 | 7.0 | 1.0 | 1.0 | 2.0 | 1.0 | 5.0 | 1.0 | 4.0 | 2.0 | 2.0 | 2.0 | 2.0 | 1.0 | Arts & Hum | 11-20 | female | 1 |
| 2 | 1.0 | 6.0 | 3.0 | 1.0 | 5.0 | 3.0 | 5.0 | 2.0 | 1.0 | 1.0 | 2.0 | 1.0 | 2.0 | 1.0 | 1.0 | Life & Biomed | 21-30 | female | 1 |
| 4 | 7.0 | 1.0 | 3.0 | 1.0 | 1.0 | 7.0 | 1.0 | 3.0 | 1.0 | 3.0 | 1.0 | 1.0 | 1.0 | 2.0 | 1.0 | Arts & Hum | 21-30 | male | 1 |
| 5 | 7.0 | 2.0 | 6.0 | 3.0 | 6.0 | 5.0 | 2.0 | 7.0 | 1.0 | 1.0 | 2.0 | 7.0 | 2.0 | 1.0 | 1.0 | Social Science | 11-20 | male | 1 |
| 6 | 5.0 | 5.0 | 3.0 | 2.0 | 2.0 | 5.0 | 5.0 | 3.0 | 3.0 | 3.0 | 4.0 | 1.0 | 2.0 | 1.0 | 2.0 | Life & Biomed | 11-20 | female | 1 |

In [8]:

```
#  data for pymc model

gender_idx = df_bin.gender.cat.codes.values
field_idx = df_bin.field.cat.codes.values
seniority_idx = df_bin.seniority.cat.codes.values
freq_idx = df_bin.FREQ.values

# coordinates for the pymc model
gender_codes = df_bin.gender.cat.categories.values
field_codes = df_bin.field.cat.categories.values
seniority_codes = df_bin.seniority.cat.categories.values


# dict with coordinates to put into the model
coords = {'s_n':seniority_codes,'g_n':gender_codes,
          'f_n':field_codes}
```

# Total effect of gender¶

This is not preregistered, and we will not report it in the paper, but just for interest: the total effect of gender.

In [9]:

```
with pm.Model(coords=coords) as gender_hyp2_total:

    # data

    G = pm.MutableData("G", gender_idx)

    
    # priors for the intercept and gender parameters
    
    alpha = pm.Normal('alpha', 0,1)

    gender = pm.Normal("gender", 0.0, 1, dims = 'g_n')
    

    p = pm.invlogit(alpha + gender[G]) # 

    y = pm.Bernoulli("y", p = p, observed=freq_idx)
    
    # get prior predictive samples to look at the priors
    
    pr = pm.sample_prior_predictive()
```

```
Sampling: [alpha, gender, y]
```

In [ ]:

```
# plot the priors for this model

variables = ['alpha','gender']
fig, axs = plt.subplots(ncols = 2,figsize = (8,2))

for ax, var in zip(axs.flat, variables):
    if len(pr.prior[var].shape) > 2:
        az.plot_posterior(pr.prior[var][:,:,0], ax=ax)
    else:
        az.plot_posterior(pr.prior[var][:,:], ax=ax)
```

In [ ]:

```
# get an estimate of the posteriors
# 4 chains

with gender_hyp2_total:
    trace_total = pm.sample(10000,
                            tune = 1000,
                            random_seed = SEED,
                            target_accept = 0.8)
```

In [ ]:

```
# save the trace

trace_total.to_netcdf('...')
```

In [10]:

```
# load the trace

trace_total = az.from_netcdf('...')
```

In [11]:

```
az.summary(trace_total)
```

Out[11]:

|  | mean | sd | hdi\_3% | hdi\_97% | mcse\_mean | mcse\_sd | ess\_bulk | ess\_tail | r\_hat |
| --- | --- | --- | --- | --- | --- | --- | --- | --- | --- |
| alpha | 0.534 | 0.576 | -0.528 | 1.641 | 0.007 | 0.005 | 6744.0 | 7694.0 | 1.0 |
| gender[female] | 0.448 | 0.582 | -0.659 | 1.539 | 0.007 | 0.005 | 6866.0 | 7918.0 | 1.0 |
| gender[male] | 0.100 | 0.580 | -0.984 | 1.188 | 0.007 | 0.005 | 6817.0 | 7918.0 | 1.0 |

## Results¶

In [12]:

```
# plot difference between coefficients female and male

fig, ax = plt.subplots(figsize = (5,2))

male = trace_total.posterior['gender'].loc[:,:,'male']
female = trace_total.posterior['gender'].loc[:,:,'female']

az.plot_posterior(female-male, ax=ax)
ax.set_title('Difference in coefficients for female and male')

plt.show()
```

In [13]:

```
# generate posterior predictive samples
# we generate them for the entire sample set to male (other characteristics intact) and female (idem)


ppcs = {}
with gender_hyp2_total:
    for i in range(2):
        pm.set_data({"G": np.repeat(i, len(df_bin))})
        ppc = pm.sample_posterior_predictive(trace_total, progressbar = False)
        ppcs[i] = ppc
```

```
Sampling: [y]
Sampling: [y]
```

In [14]:

```
#posterior predictive of the proportions of female respondents with FREQ == 1 minus the proprtion of male respondents with FREQ == 1


female = az.extract(ppcs[0].posterior_predictive['y'])['y'].values.sum(axis = 0) / len(df_bin)
male = az.extract(ppcs[1].posterior_predictive['y'])['y'].values.sum(axis = 0) / len(df_bin)

fig, ax = plt.subplots()
sns.kdeplot(np.array(female-male),fill=True, ax=ax)
ax.axvline(0, color = 'r')
ax.set_xlabel('difference in proportion of FREQ == 1')


plt.show()
```

In [15]:

```
# compare counts of FREQ

female = np.random.choice(np.ravel(ppcs[0].posterior_predictive['y'].values), size = 20000, replace=True)
male = np.random.choice(np.ravel(ppcs[1].posterior_predictive['y'].values), size = 20000, replace=True)

data = pd.DataFrame([female, male], index = ['female','male']).T.stack().reset_index().rename(columns = {'level_1':'gender',0:'FREQ'})
sns.countplot(data=data, x = 'FREQ',hue = 'gender')
```

Out[15]:

```
<Axes: xlabel='FREQ', ylabel='count'>
```

# Direct effect of gender¶

In [16]:

```
with pm.Model(coords=coords) as gender_hyp2:

    # data

    G = pm.MutableData("G", gender_idx)
    F = pm.MutableData("F", field_idx)
    S = pm.MutableData("S", seniority_idx)


    # fixed hyperpriors for field, seniority
    
    s_field = pm.Uniform('s_field', 0,3)
    s_seniority = pm.Uniform('s_seniority',0,3)
    

    # variable priors for the demographic predictors
    # non-centered to make sampling easier
    
    gender = pm.Normal("gender", 0.0, 1, dims = 'g_n')
    
    z_field = pm.Normal("z_field", 0.0, 1, dims = 'f_n')
    field = pm.Deterministic("field", z_field * s_field, dims = 'f_n')
    
    z_seniority = pm.Normal("z_seniority", 0.0, 1.0, dims = 's_n')
    seniority = pm.Deterministic("seniority", z_seniority * s_seniority, dims = 's_n')

    p = gender[G] +  field[F] + seniority[S] # 

    y = pm.Bernoulli("y", logit_p = p,  observed=freq_idx)
    
    # sample from the priors to look at them
    
    pr = pm.sample_prior_predictive()
```

```
Sampling: [gender, s_field, s_seniority, y, z_field, z_seniority]
```

In [17]:

```
# plot the priors

variables = ['gender','s_field','s_seniority','field','seniority']
fig, axs = plt.subplots(2,3,figsize = (15,7))

for ax, var in zip(axs.flat, variables):
    if len(pr.prior[var].shape) > 2:
        az.plot_posterior(pr.prior[var][:,:,0], ax=ax)
    else:
        az.plot_posterior(pr.prior[var][:,:], ax=ax)
```

In [ ]:

```
# draw samples from the posterior

with gender_hyp2:
    trace = pm.sample(10000,
                      tune = 2000,
                      return_inferencedata = True,
                      random_seed = SEED,
                      target_accept = 0.97)
```

In [ ]:

```
# save the trace for later use

trace.to_netcdf('...')
```

In [18]:

```
# load the trace

trace = az.from_netcdf('...')
```

In [19]:

```
# all coefficients

az.summary(trace)
```

Out[19]:

|  | mean | sd | hdi\_3% | hdi\_97% | mcse\_mean | mcse\_sd | ess\_bulk | ess\_tail | r\_hat |
| --- | --- | --- | --- | --- | --- | --- | --- | --- | --- |
| gender[female] | 0.764 | 0.339 | 0.104 | 1.387 | 0.003 | 0.002 | 12761.0 | 13379.0 | 1.0 |
| gender[male] | 0.370 | 0.330 | -0.290 | 0.959 | 0.003 | 0.002 | 12049.0 | 13001.0 | 1.0 |
| z\_field[Arts & Hum] | -0.598 | 0.658 | -1.856 | 0.600 | 0.005 | 0.003 | 18198.0 | 25909.0 | 1.0 |
| z\_field[Life & Biomed] | 1.255 | 0.646 | 0.097 | 2.485 | 0.005 | 0.003 | 18618.0 | 21827.0 | 1.0 |
| z\_field[Natural Science] | -0.228 | 0.608 | -1.372 | 0.900 | 0.005 | 0.003 | 17578.0 | 25352.0 | 1.0 |
| z\_field[Social Science] | -0.003 | 0.604 | -1.187 | 1.109 | 0.004 | 0.003 | 18940.0 | 24353.0 | 1.0 |
| z\_field[Tech & Engineering] | 0.149 | 0.641 | -1.110 | 1.342 | 0.004 | 0.003 | 22786.0 | 26215.0 | 1.0 |
| z\_seniority[0-10] | 0.255 | 0.850 | -1.372 | 1.858 | 0.005 | 0.004 | 33479.0 | 27187.0 | 1.0 |
| z\_seniority[11-20] | 0.476 | 0.782 | -1.085 | 1.938 | 0.005 | 0.004 | 27455.0 | 23357.0 | 1.0 |
| z\_seniority[21-30] | 0.389 | 0.760 | -1.062 | 1.858 | 0.004 | 0.004 | 29084.0 | 23916.0 | 1.0 |
| z\_seniority[31-40] | -0.446 | 0.802 | -1.994 | 1.055 | 0.005 | 0.004 | 29298.0 | 25398.0 | 1.0 |
| z\_seniority[>40] | -0.370 | 0.822 | -1.920 | 1.220 | 0.005 | 0.004 | 32817.0 | 25450.0 | 1.0 |
| s\_field | 0.569 | 0.357 | 0.110 | 1.230 | 0.004 | 0.003 | 10907.0 | 13657.0 | 1.0 |
| s\_seniority | 0.293 | 0.274 | 0.000 | 0.729 | 0.003 | 0.002 | 10798.0 | 15283.0 | 1.0 |
| field[Arts & Hum] | -0.266 | 0.324 | -0.898 | 0.339 | 0.002 | 0.002 | 18391.0 | 18377.0 | 1.0 |
| field[Life & Biomed] | 0.609 | 0.330 | 0.044 | 1.262 | 0.003 | 0.002 | 13599.0 | 14781.0 | 1.0 |
| field[Natural Science] | -0.080 | 0.314 | -0.679 | 0.521 | 0.003 | 0.002 | 15724.0 | 16562.0 | 1.0 |
| field[Social Science] | 0.027 | 0.319 | -0.564 | 0.658 | 0.003 | 0.002 | 15633.0 | 17251.0 | 1.0 |
| field[Tech & Engineering] | 0.101 | 0.343 | -0.526 | 0.770 | 0.003 | 0.002 | 17259.0 | 18223.0 | 1.0 |
| seniority[0-10] | 0.097 | 0.262 | -0.349 | 0.638 | 0.002 | 0.002 | 24237.0 | 21946.0 | 1.0 |
| seniority[11-20] | 0.144 | 0.235 | -0.227 | 0.614 | 0.002 | 0.002 | 19143.0 | 19459.0 | 1.0 |
| seniority[21-30] | 0.119 | 0.224 | -0.245 | 0.578 | 0.002 | 0.002 | 20376.0 | 19147.0 | 1.0 |
| seniority[31-40] | -0.112 | 0.225 | -0.571 | 0.274 | 0.002 | 0.001 | 23942.0 | 21986.0 | 1.0 |
| seniority[>40] | -0.101 | 0.236 | -0.609 | 0.301 | 0.002 | 0.001 | 26293.0 | 22851.0 | 1.0 |

## sampling stats¶

In [20]:

```
#R-hat (we used three chains) and ESS

az.summary(trace, var_names = ['gender','field','seniority','s_field', 's_seniority'])[['r_hat', 'ess_bulk']].T
```

Out[20]:

|  | gender[female] | gender[male] | field[Arts & Hum] | field[Life & Biomed] | field[Natural Science] | field[Social Science] | field[Tech & Engineering] | seniority[0-10] | seniority[11-20] | seniority[21-30] | seniority[31-40] | seniority[>40] | s\_field | s\_seniority |
| --- | --- | --- | --- | --- | --- | --- | --- | --- | --- | --- | --- | --- | --- | --- |
| r\_hat | 1.0 | 1.0 | 1.0 | 1.0 | 1.0 | 1.0 | 1.0 | 1.0 | 1.0 | 1.0 | 1.0 | 1.0 | 1.0 | 1.0 |
| ess\_bulk | 12761.0 | 12049.0 | 18391.0 | 13599.0 | 15724.0 | 15633.0 | 17259.0 | 24237.0 | 19143.0 | 20376.0 | 23942.0 | 26293.0 | 10907.0 | 10798.0 |

In [21]:

```
#see here: https://docs.pymc.io/en/v3/pymc-examples/examples/diagnostics_and_criticism/sampler-stats.html

#print number of divergences, ideally 0
print(f'divergences: {trace.sample_stats["diverging"].values.sum()}')

#print the acceptance rate
print(f'mean acceptance rate: {trace.sample_stats["acceptance_rate"].values.mean()}')

#compare the overall distribution of the energy levels with the change of energy between successive samples. Ideally, they should be very similar
az.plot_energy(trace, figsize=(6, 4));
```

```
divergences: 0
mean acceptance rate: 0.9683058380352729
```

## Results¶

In [22]:

```
# plot difference between coefficients female and male

fig, ax = plt.subplots(figsize = (5,2))

male = trace.posterior['gender'].loc[:,:,'male']
female = trace.posterior['gender'].loc[:,:,'female']

az.plot_posterior(female-male, round_to= 3 ,ax=ax)

plt.show()
```

In [23]:

```
# for table 3 in the paper: get the description of the distribution of the difference

az.summary(female-male)
```

Out[23]:

|  | mean | sd | hdi\_3% | hdi\_97% | mcse\_mean | mcse\_sd | ess\_bulk | ess\_tail | r\_hat |
| --- | --- | --- | --- | --- | --- | --- | --- | --- | --- |
| gender | 0.394 | 0.204 | -0.001 | 0.767 | 0.001 | 0.001 | 57210.0 | 28852.0 | 1.0 |

In [24]:

```
# generate posterior predictive samples
# we generate them for the entire sample set to male (other characteristics intact) and female (idem)


ppcs = {}
with gender_hyp2:
    for i in range(2):
        pm.set_data({"G": np.repeat(i, len(df_bin))})
        ppc = pm.sample_posterior_predictive(trace, progressbar = False)
        ppcs[i] = ppc
```

```
Sampling: [y]
Sampling: [y]
```

In [25]:

```
#posterior predictive of the proportions of female respondents with FREQ == 1 minus the proprtion of male respondents with FREQ == 1


female = az.extract(ppcs[0].posterior_predictive['y'])['y'].values.sum(axis = 0) / len(df_bin)
male = az.extract(ppcs[1].posterior_predictive['y'])['y'].values.sum(axis = 0) / len(df_bin)

fig, ax = plt.subplots()
sns.kdeplot(np.array(female-male),fill=True, ax=ax)
ax.axvline(0, color = 'r')
ax.set_xlabel('proportion')
ax.set_title('Gender test 2: Difference in proportion of FREQ == 1', fontsize = 20, pad = 20)

fig.tight_layout()
plt.savefig('...', dpi = 300)

plt.show()
```

In [26]:

```
# compare posterior predictive counts of FREQ

female = np.random.choice(np.ravel(ppcs[0].posterior_predictive['y'].values), size = 20000, replace=True)
male = np.random.choice(np.ravel(ppcs[1].posterior_predictive['y'].values), size = 20000, replace=True)

data = pd.DataFrame([female, male], index = ['female','male']).T.stack().reset_index().rename(columns = {'level_1':'gender',0:'FREQ'})

# save data to make figures for paper in other notebook

with open('...', 'wb') as handle:
    pickle.dump(data, handle, protocol=pickle.HIGHEST_PROTOCOL)


    
sns.countplot(data=data, x = 'FREQ',hue = 'gender')
```

Out[26]:

```
<Axes: xlabel='FREQ', ylabel='count'>
```

In [ ]:

```

```
