# Supplementary material for: Questionable research practices in competitive grant funding: A survey
Source: PLoS One. 2023 Nov 2;18(11):e0293310. doi: 10.1371/journal.pone.0293310 (PMC10621923; doi:10.1371/journal.pone.0293310)
Supplement: S3 File — The code can also be accessed through the OSF page of the project (https://osf.io/jk6wd/). (ZIP) [file pone.0293310.s015.zip › S13 File/ERF_gender_hypothesis3_upload.html]

ERF\_gender\_hypothesis3\_upload


In [1]:

```
import pandas as pd
import numpy as np
import pickle

# for the stats
import pymc as pm
import bambi as bmb
from scipy.special import expit, logit


# for plotting
import seaborn as sns
import matplotlib.pyplot as plt
import arviz as az

# for DAG

import collections.abc
#causalgraphicalmodels needs the four following aliases to be done manually.
collections.Iterable = collections.abc.Iterable
collections.Mapping = collections.abc.Mapping
collections.MutableSet = collections.abc.MutableSet
collections.MutableMapping = collections.abc.MutableMapping
from causalgraphicalmodels import CausalGraphicalModel
import daft
```

In [2]:

```
# versions

print('\n'.join(f'{m.__name__}=={m.__version__}' for m in globals().values() if getattr(m, '__version__', None)))
```

```
pandas==1.5.3
numpy==1.24.2
pymc==5.1.2
bambi==0.10.0
seaborn==0.12.2
arviz==0.15.1
daft==0.1.2
```

In [3]:

```
sns.set_palette("Dark2")
sns.set_style("darkgrid")
sns.set_context("paper", font_scale=1.5)
```

In [4]:

```
# seed

SEED = 2808

np.random.seed(SEED)
```

# Hypothesis¶

## Description¶

**GENDER HYPOTHESIS 3: Responses to individual qrp questions are more likely to be 'at least sometimes' if the responder identifies as male than if the respondent identies as female'.**

note: In the preregistration we had written that we would leave penalist qrps out of this analysis as it would force us to omit from the data any respondent that hasn't filled in the 'panelist' questions. However, this is not true, as this is an item-level outcome variable. Hence, we have included all responses, regardless of which roles the respondents filled in. This has not affected the result in a substantial way. The results with the originally specified dataset can be accessed through the OSF page of the project, or by running the models here limiting the dataset to respondents that filled in all r\_qrp and a\_qrp questions.

## Reporting¶

In the preregistered analysis code, we had written that we would report model 1 in the paper. However, seeing that, like in the pilot, the influence of question type and individual is much larger than that of gender, we think it better to report the model that includes these in the paper. The other models do not give different results with respect to the hypothesis (i.e. no effect for gender), and are still accessible through the OSF page of the project.

## Justification¶

We expected higher qrp levels for those who identify as male on the basis of:

- the pilot study (see results below, generally a weak effect of 'male' increasing the probability of higher ordinal responses.
- existing research: Fang et al. 2013 doi: 10.1128/mBio.00640-12 find that in reports of scientific misconduct (fraud, fabrication, and plagiarism) the proportion of males is higher than the proportion of males in the relevant disciplines. In line with this, Gopalakrishna et al 2022 doi: 10.1371/journal.pone.0263023 find that being a male increased the odds of various qrps. While one other study did not find this in an analysis of 120 cases of wrongdoing (of which 40 FFP and 40 other research misconduct), we think the evidence in favour of this hypothesis is stronger, in particular due to the much larger sample size and scope of the Gopalakrishna study, and the fact that the results are weakly confirmed by our pilot study.
- We choose to compare 'male' with 'female' instead of 'non-male' (which includes nonbinary and other) because potential explanations involve societal roles of men/women and levels of testosterone. These explanations are easier to evaluate if the hypothesis focuses on men and women. In the exploratory analysis, we will rerun the same analysis with male vs non-male, and with all gender categories.
- field and career stage are included in the DAG because previous research suggests they might play a role. We did not include 'continent' because we have no reason (neither pilot results nor previous research) to think it is relevant. In addition, our samples will be mostly european, meaning that we'll have too little data from other continents for meaningfull comparison.

# DAG¶

In [5]:

```
dag_gender = CausalGraphicalModel(nodes=["QRP", "G", "Age",'Field', 'Ind','Q'], 
                              edges=[("Age", "QRP"), ("Age", "QRP"), ("Field", "QRP"), 
                                     ('G','Age'),('G','QRP'),('G','Field'), ('Age','Field'),
                                    ('G','Ind'),('Field','Ind'),('Age','Ind'), ('Ind','QRP'),
                                    ('Q','QRP')])
dag_gender.draw()
dag_gender.draw()
```

Out[5]:

Ind

Ind


QRP

QRP


Ind->QRP


G

G


G->Ind


Age

Age


G->Age


G->QRP


Field

Field


G->Field


Age->Ind


Age->QRP


Age->Field


Q

Q


Q->QRP


Field->Ind


Field->QRP

In [6]:

```
def backdoor(dag, predictor, outcome):
    all_adjustment_sets = dag.get_all_backdoor_adjustment_sets(predictor, outcome)
    for s in all_adjustment_sets:
        if all(not t.issubset(s) for t in all_adjustment_sets if t != s):
            if s != {"U"}:
                print(s)

                
# For gender

backdoor(dag_gender,'G','QRP')
```

```
frozenset()
```

note: We are interested in the direct effect of gender. Hence, we will include seniority, field, individual as controls and gender variable of interest. We will also include question as a competing cause. This model will give us an estimate of the direct effect of gender, the direct effect of individuals, and the effect of questions.

# Indices & Data¶

In [7]:

```
# data

real_data = pd.read_csv('...')

# columns with QRPs

r_qrp = ['R4', 'R5', 'R6','R7','R8']
a_qrp = ['A2','A3','A5', 'A6','A8', 'A9', 'A10', 'A11','A14','A15']
p_qrp = ['P4']
demo = ['C1', 'C3', 'C4']

# select the relevant columns for this test

df = real_data[r_qrp + p_qrp + a_qrp + demo].reset_index().copy()

# put df into long form
df = df.melt(id_vars= demo + ['index'])
df.columns = ['field','seniority','gender','ind','question','score']

# drop other genders
other_genders = len((df.loc[(df.gender != 'male') & (df.gender != 'female')]))
df = df.loc[(df.gender == 'male') | (df.gender == 'female')]

print(f'number of nonmale/female responses removed: {other_genders}')

# set scores to start at 0 instead of 1

df['score'] = df.score - 1

# change dtype to categorical for the categorical columns
df[['field','seniority','gender','ind','question']] = df[['field','seniority','gender','ind','question']].astype('category')
df = df.replace({7:0})

# create ALS score

df['ALS'] = np.where(df.score > 0, 1, 0)

#complete case analyses: dropna

predrop = len(df)
df = df.dropna()
postdrop = len(df)

print(f'rows with nans removed: {predrop - postdrop}')
print(f'total responses: {postdrop}')

df.head()
```

```
number of nonmale/female responses removed: 336
rows with nans removed: 2088
total responses: 8840
```

Out[7]:

|  | field | seniority | gender | ind | question | score | ALS |
| --- | --- | --- | --- | --- | --- | --- | --- |
| 1 | Arts & Hum | 11-20 | female | 1 | R4 | 1.0 | 1 |
| 2 | Life & Biomed | 21-30 | female | 2 | R4 | 1.0 | 1 |
| 3 | Arts & Hum | 21-30 | female | 3 | R4 | 0.0 | 0 |
| 4 | Arts & Hum | 21-30 | male | 4 | R4 | 0.0 | 0 |
| 5 | Social Science | 11-20 | male | 5 | R4 | 1.0 | 1 |

In [8]:

```
# data from how many respondents

len(df.ind.unique())
```

Out[8]:

```
678
```

In [9]:

```
#  coordinates and data for pymc model
q_idx = df.question.cat.codes.values
s_idx = df.seniority.cat.codes.values
g_idx = df.gender.cat.codes.values
i_idx = df.ind.cat.codes.values
f_idx = df.field.cat.codes.values
als_idx = df.ALS.values


# The 9 position names
q_codes = df.question.cat.categories.values
s_codes = df.seniority.cat.categories.values
g_codes = df.gender.cat.categories.values
i_codes = df.ind.cat.categories.values
f_codes = df.field.cat.categories.values


coords = {'q_n':q_codes, 's_n':s_codes,'g_n':g_codes,'i_n':i_codes,
          'f_n':f_codes}
```

# Total effect of gender¶

In [10]:

```
with pm.Model(coords=coords) as gender_hyp3_total:

    # data

    G = pm.MutableData("G", g_idx)

    
    # intercept and gender priors
    
    alpha = pm.Normal('alpha', 0,1)
    gender = pm.Normal("gender", 0.0, 1, dims = 'g_n')
    

    p = pm.invlogit(alpha + gender[G]) # 

    y = pm.Bernoulli("y", p = p, observed=als_idx)
    
    # get prior samples
    
    pr = pm.sample_prior_predictive()
```

```
Sampling: [alpha, gender, y]
```

In [11]:

```
# plot the priors

variables = ['alpha','gender']
fig, axs = plt.subplots(ncols = 2,figsize = (8,2))

for ax, var in zip(axs.flat, variables):
    if len(pr.prior[var].shape) > 2:
        az.plot_posterior(pr.prior[var][:,:,0], ax=ax)
    else:
        az.plot_posterior(pr.prior[var][:,:], ax=ax)
```

In [ ]:

```
# sample from the posterior

with gender_hyp3_total:
    trace_total = pm.sample(10000,
                            tune = 1000,
                            random_seed = SEED,
                            target_accept = 0.8)
```

In [13]:

```
# save the trace for later use

# trace_total.to_netcdf('...')

# to load

trace_total = az.from_netcdf('...')
```

In [14]:

```
az.summary(trace_total)
```

Out[14]:

|  | mean | sd | hdi\_3% | hdi\_97% | mcse\_mean | mcse\_sd | ess\_bulk | ess\_tail | r\_hat |
| --- | --- | --- | --- | --- | --- | --- | --- | --- | --- |
| alpha | -0.127 | 0.58 | -1.267 | 0.917 | 0.009 | 0.006 | 4375.0 | 5038.0 | 1.0 |
| gender[female] | -0.023 | 0.58 | -1.069 | 1.114 | 0.009 | 0.006 | 4379.0 | 5007.0 | 1.0 |
| gender[male] | -0.078 | 0.58 | -1.121 | 1.061 | 0.009 | 0.006 | 4376.0 | 5079.0 | 1.0 |

## Results¶

In [15]:

```
# plot difference between coefficients female and male

fig, ax = plt.subplots(figsize = (5,2))

male = trace_total.posterior['gender'].loc[:,:,'male']
female = trace_total.posterior['gender'].loc[:,:,'female']

az.plot_posterior(female-male, ax=ax)
ax.set_title('Difference in coefficients for female and male')

plt.show()
```

In [16]:

```
# generate posterior predictive samples
# we generate them for the entire sample set to male (other characteristics intact) and female (idem)


ppcs = {}
with gender_hyp3_total:
    for i in range(2):
        pm.set_data({"G": np.repeat(i, len(df))})
        ppc = pm.sample_posterior_predictive(trace_total, progressbar = False)
        ppcs[i] = ppc
```

```
Sampling: [y]
Sampling: [y]
```

In [17]:

```
#posterior predictive of the proportions of female respondents with FREQ == 1 minus the proprtion of male respondents with FREQ == 1

female = az.extract(ppcs[0].posterior_predictive['y'])['y'].values.sum(axis = 0) / len(df)
male = az.extract(ppcs[1].posterior_predictive['y'])['y'].values.sum(axis = 0) / len(df)

fig, ax = plt.subplots()
sns.kdeplot(np.array(female-male),fill=True, ax=ax)
ax.axvline(0, color = 'r')
ax.set_xlabel('difference in proportion of ALS == 1')

plt.show()
```

In [18]:

```
# compare counts of ALS

female = np.random.choice(np.ravel(ppcs[0].posterior_predictive['y'].values), size = 20000, replace=True)
male = np.random.choice(np.ravel(ppcs[1].posterior_predictive['y'].values), size = 20000, replace=True)

data = pd.DataFrame([female, male], index = ['female','male']).T.stack().reset_index().rename(columns = {'level_1':'gender',0:'ALS'})
sns.countplot(data=data, x = 'ALS',hue = 'gender')
```

Out[18]:

```
<Axes: xlabel='ALS', ylabel='count'>
```

# Direct effect of gender¶

Prior for the variation between questions and individuals a bit wider, as we saw way more variation there than between fields or levels of seniority in the pilot.

In [19]:

```
# add field, seniority, question and individual to the model
# HalfCauchy or HalfNormal priors for the variation hyperpriors gives the same results, but gets divergences

with pm.Model(coords=coords) as gender_hyp3:

    # data

    G = pm.MutableData("G", g_idx)
    F = pm.MutableData("F", f_idx)
    S = pm.MutableData("S", s_idx)
    Q = pm.MutableData("Q", q_idx)
    I = pm.MutableData("I", i_idx)

    # fixed hyperpriors for field, seniority and participant
    
    s_ind = pm.Uniform('s_ind', 0,3)
    s_field = pm.Uniform('s_field', 0,2)
    s_seniority = pm.Uniform('s_seniority',0,2)
    s_question = pm.Uniform('s_question',0,3)
    
    # variable priors for the demographic predictors
    # non-centered to make sampling easier
    
    gender = pm.Normal("gender", 0.0, 1, dims = 'g_n')
    
    z_field = pm.Normal("z_field", 0.0, 1, dims = 'f_n')
    field = pm.Deterministic("field", z_field * s_field, dims = 'f_n')
    
    z_question = pm.Normal("z_question", 0.0, 1, dims = 'q_n')
    question = pm.Deterministic("question", z_question * s_question, dims = 'q_n')
    
    z_seniority = pm.Normal("z_seniority", 0.0, 1.0, dims = 's_n')
    seniority = pm.Deterministic("seniority", z_seniority * s_seniority, dims = 's_n')
    
    z_ind = pm.Normal("z_ind", 0.0, 1, dims = 'i_n')
    ind = pm.Deterministic("ind", z_ind * s_ind, dims = 'i_n')

    p = gender[G] +  ind[I] + field[F] + seniority[S] + question[Q] # 

    y = pm.Bernoulli("y", logit_p = p,  observed=als_idx)
    
    pr = pm.sample_prior_predictive()
```

```
Sampling: [gender, s_field, s_ind, s_question, s_seniority, y, z_field, z_ind, z_question, z_seniority]
```

In [20]:

```
# plot the priors

variables = ['gender','s_field','s_ind','s_seniority','ind','field','seniority','question']
fig, axs = plt.subplots(2,4,figsize = (15,7))

for ax, var in zip(axs.flat, variables):
    if len(pr.prior[var].shape) > 2:
        az.plot_posterior(pr.prior[var][:,:,0], ax=ax)
    else:
        az.plot_posterior(pr.prior[var][:,:], ax=ax)
```

In [ ]:

```
# sample from the posterior
# 4 chains
# the high target_accept means that the sampling is very slow, but it is needed to avoid divergences

with gender_hyp3:
    trace = pm.sample(10000,
                      tune = 2000,
                      return_inferencedata = True,
                      random_seed = SEED,
                      target_accept = 0.99)
```

In [21]:

```
# save the trace for later use

# trace.to_netcdf('...')

# to load

trace = az.from_netcdf('...')
```

In [22]:

```
# don't print results for 'individual', as there are almost 700 parameters for that one.

az.summary(trace, var_names = ['gender','field','seniority', 's_ind','s_field', 's_seniority'])
```

Out[22]:

|  | mean | sd | hdi\_3% | hdi\_97% | mcse\_mean | mcse\_sd | ess\_bulk | ess\_tail | r\_hat |
| --- | --- | --- | --- | --- | --- | --- | --- | --- | --- |
| gender[female] | -0.262 | 0.292 | -0.810 | 0.286 | 0.004 | 0.003 | 4556.0 | 9327.0 | 1.0 |
| gender[male] | -0.329 | 0.288 | -0.881 | 0.202 | 0.004 | 0.003 | 4452.0 | 9003.0 | 1.0 |
| field[Arts & Hum] | -0.292 | 0.209 | -0.707 | 0.074 | 0.002 | 0.001 | 13459.0 | 16462.0 | 1.0 |
| field[Life & Biomed] | 0.301 | 0.190 | -0.044 | 0.676 | 0.002 | 0.001 | 12844.0 | 17911.0 | 1.0 |
| field[Natural Science] | -0.056 | 0.199 | -0.438 | 0.319 | 0.002 | 0.001 | 13801.0 | 18413.0 | 1.0 |
| field[Social Science] | -0.036 | 0.197 | -0.429 | 0.318 | 0.002 | 0.001 | 13011.0 | 16838.0 | 1.0 |
| field[Tech & Engineering] | 0.011 | 0.205 | -0.361 | 0.417 | 0.002 | 0.001 | 15110.0 | 19555.0 | 1.0 |
| seniority[0-10] | -0.018 | 0.116 | -0.255 | 0.202 | 0.001 | 0.001 | 23106.0 | 21321.0 | 1.0 |
| seniority[11-20] | 0.076 | 0.111 | -0.096 | 0.301 | 0.001 | 0.001 | 12774.0 | 19189.0 | 1.0 |
| seniority[21-30] | -0.041 | 0.103 | -0.245 | 0.142 | 0.001 | 0.001 | 18989.0 | 20789.0 | 1.0 |
| seniority[31-40] | -0.007 | 0.104 | -0.212 | 0.194 | 0.001 | 0.001 | 23652.0 | 24282.0 | 1.0 |
| seniority[>40] | -0.026 | 0.113 | -0.259 | 0.179 | 0.001 | 0.001 | 23733.0 | 22015.0 | 1.0 |
| s\_ind | 1.155 | 0.051 | 1.061 | 1.252 | 0.000 | 0.000 | 14949.0 | 24806.0 | 1.0 |
| s\_field | 0.356 | 0.219 | 0.082 | 0.747 | 0.002 | 0.002 | 10269.0 | 13822.0 | 1.0 |
| s\_seniority | 0.130 | 0.127 | 0.000 | 0.327 | 0.001 | 0.001 | 7519.0 | 15796.0 | 1.0 |

## sampling stats¶

In [23]:

```
#R-hat (we used three chains) and ESS

az.summary(trace, var_names = ['gender','field','seniority', 's_ind','s_field', 's_seniority'])[['r_hat', 'ess_bulk']].T
```

Out[23]:

|  | gender[female] | gender[male] | field[Arts & Hum] | field[Life & Biomed] | field[Natural Science] | field[Social Science] | field[Tech & Engineering] | seniority[0-10] | seniority[11-20] | seniority[21-30] | seniority[31-40] | seniority[>40] | s\_ind | s\_field | s\_seniority |
| --- | --- | --- | --- | --- | --- | --- | --- | --- | --- | --- | --- | --- | --- | --- | --- |
| r\_hat | 1.0 | 1.0 | 1.0 | 1.0 | 1.0 | 1.0 | 1.0 | 1.0 | 1.0 | 1.0 | 1.0 | 1.0 | 1.0 | 1.0 | 1.0 |
| ess\_bulk | 4556.0 | 4452.0 | 13459.0 | 12844.0 | 13801.0 | 13011.0 | 15110.0 | 23106.0 | 12774.0 | 18989.0 | 23652.0 | 23733.0 | 14949.0 | 10269.0 | 7519.0 |

In [24]:

```
#see here: https://docs.pymc.io/en/v3/pymc-examples/examples/diagnostics_and_criticism/sampler-stats.html

#print number of divergences, ideally 0
print(f'divergences: {trace.sample_stats["diverging"].values.sum()}')

#print the acceptance rate
print(f'mean acceptance rate: {trace.sample_stats["acceptance_rate"].values.mean()}')

#compare the overall distribution of the energy levels with the change of energy between successive samples. Ideally, they should be very similar
az.plot_energy(trace, figsize=(6, 4));
```

```
divergences: 0
mean acceptance rate: 0.9878999348127678
```

## Results¶

In [25]:

```
# plot difference between coefficients female and male

fig, ax = plt.subplots(figsize = (5,2))

male = trace.posterior['gender'].loc[:,:,'male']
female = trace.posterior['gender'].loc[:,:,'female']

az.plot_posterior(female-male, round_to = 3, ax=ax)

plt.show()
```

In [26]:

```
# for table 3 in the paper: get the description of the distribution of the difference

az.summary(female-male)
```

Out[26]:

|  | mean | sd | hdi\_3% | hdi\_97% | mcse\_mean | mcse\_sd | ess\_bulk | ess\_tail | r\_hat |
| --- | --- | --- | --- | --- | --- | --- | --- | --- | --- |
| gender | 0.067 | 0.108 | -0.134 | 0.271 | 0.001 | 0.001 | 14174.0 | 21912.0 | 1.0 |

In [27]:

```
# generate posterior predictive samples
# we generate them for the entire sample set to male (other characteristics intact) and female (idem)

ppcs = {}
with gender_hyp3:
    for i in range(2):
        pm.set_data({"G": np.repeat(i, len(df))})
#         thinned_trace = trace.sel(draw=slice(None, None, 5))
        ppc = pm.sample_posterior_predictive(trace, progressbar = False)
        ppcs[i] = ppc
```

```
Sampling: [y]
Sampling: [y]
```

In [28]:

```
# plot posterior predictive differences between female and male for proportion FREQ = 1 

female = az.extract(ppcs[0].posterior_predictive['y'])['y'].values.sum(axis = 0) / len(df)
male = az.extract(ppcs[1].posterior_predictive['y'])['y'].values.sum(axis = 0) / len(df)

fig, ax = plt.subplots()
sns.kdeplot(np.array(female-male),fill=True, ax=ax)
ax.axvline(0, color = 'r')
ax.set_xlabel('difference in proportion of ALS == 1')

plt.show()
```

In [29]:

```
# compare counts of ALS

female = np.random.choice(np.ravel(ppcs[0].posterior_predictive['y'].values), size = 20000, replace=True)
male = np.random.choice(np.ravel(ppcs[1].posterior_predictive['y'].values), size = 20000, replace=True)

data = pd.DataFrame([female, male], index = ['female','male']).T.stack().reset_index().rename(columns = {'level_1':'gender',0:'ALS'})

# save data to make figures for paper in other notebook

with open('...', 'wb') as handle:
    pickle.dump(data, handle, protocol=pickle.HIGHEST_PROTOCOL)

sns.countplot(data=data, x = 'ALS',hue = 'gender')
```

Out[29]:

```
<Axes: xlabel='ALS', ylabel='count'>
```

In [30]:

```
# standard deviation hyper parameters
fig, ax = plt.subplots(figsize = (5,3))
az.plot_forest(trace, var_names = ['s_field','s_seniority','s_ind','s_question'], combined = True, ax=ax)
plt.show()
```

In [31]:

```
# density of mean odds ratios of individuals-parameter

fig, ax = plt.subplots(figsize = (5,3))
ind_means = np.exp(trace.posterior['ind'].mean(dim = ['chain','draw']).values)

sns.kdeplot(ind_means, ax=ax)
plt.show()
```

In [ ]:

```

```
