# Supplementary material for: Questionable research practices in competitive grant funding: A survey
Source: PLoS One. 2023 Nov 2;18(11):e0293310. doi: 10.1371/journal.pone.0293310 (PMC10621923; doi:10.1371/journal.pone.0293310)
Supplement: S3 File — The code can also be accessed through the OSF page of the project (https://osf.io/jk6wd/). (ZIP) [file pone.0293310.s015.zip › S13 File/ERF_lack_of_funding_exploration_upload.html]

ERF\_lack\_of\_funding\_exploration\_upload


In [9]:

```
import pandas as pd
import numpy as np
from pandas.api.types import CategoricalDtype

# for the stats
import pymc as pm
import bambi as bmb
from scipy.special import expit as logistic
# import aesara.tensor as at

# for plotting
import seaborn as sns
import matplotlib.pyplot as plt
import arviz as az

# for DAGs

import collections.abc
#causalgraphicalmodels needs the four following aliases to be done manually.
collections.Iterable = collections.abc.Iterable
collections.Mapping = collections.abc.Mapping
collections.MutableSet = collections.abc.MutableSet
collections.MutableMapping = collections.abc.MutableMapping
from causalgraphicalmodels import CausalGraphicalModel
import daft
```

In [2]:

```
# versions

print('\n'.join(f'{m.__name__}=={m.__version__}' for m in globals().values() if getattr(m, '__version__', None)))
```

```
pandas==1.5.3
numpy==1.24.2
pymc==5.1.2
bambi==0.10.0
seaborn==0.12.2
arviz==0.15.1
daft==0.1.2
```

In [3]:

```
sns.set_palette("Dark2")
sns.set_style("darkgrid")
sns.set_context("paper", font_scale=1.5)
```

In [4]:

```
# seed

SEED = 2808

np.random.seed(SEED)
```

# Hypothesis¶

Not preregistered, but we expect that researchers who lack funding are more likely to engage in QRPs.

# DAG¶

In [6]:

```
dagLOF = CausalGraphicalModel(nodes=["QRP", "G", "Age",'Field', 'SR', 'LOF'],
                              edges=[("Age", "QRP"), ("Age", "QRP"), ("Field", "QRP"), ('G','Age'),
                                     ('G','QRP'),('G','Field'),('SR', 'QRP'),('G','SR'), ('Age','SR'),
                                     ('Field','SR'), ('Age','Field'), ('SR','LOF'),('LOF','QRP'), 
                                     ('Field','LOF'), ('G','LOF'), ('Age','LOF')
                                    ])

dagLOF.draw()
```

Out[6]:

LOF

LOF


QRP

QRP


LOF->QRP


Age

Age


Age->LOF


Age->QRP


SR

SR


Age->SR


Field

Field


Age->Field


SR->LOF


SR->QRP


G

G


G->LOF


G->QRP


G->Age


G->SR


G->Field


Field->LOF


Field->QRP


Field->SR

In [7]:

```
def backdoor(dag, predictor, outcome):
    all_adjustment_sets = dag.get_all_backdoor_adjustment_sets(predictor, outcome)
    for s in all_adjustment_sets:
        if all(not t.issubset(s) for t in all_adjustment_sets if t != s):
            if s != {"U"}:
                print(s)

                
# For gender

backdoor(dagLOF,'LOF','QRP')
```

```
frozenset({'SR', 'G', 'Field', 'Age'})
```

# Indices & Data¶

In [10]:

```
# data

real_data = pd.read_csv('...')
r_qrp = ['R4', 'R5', 'R6','R7','R8']
a_qrp = ['A2','A3','A5', 'A6','A8', 'A9', 'A10', 'A11','A14','A15']
p_qrp = ['P4']
demo = ['C1', 'C3', 'C4']

# select the relevant columns (QRPs, lof and success)

df = real_data[r_qrp + p_qrp + a_qrp + demo + ['A20','C6_bis']].reset_index().copy()


# put in long form
df = df.melt(id_vars= demo + ['index', 'A20','C6_bis'])
df.columns = ['field','seniority','gender','ind','success','lof','question','score']

# make success and lof ordered categorical

cat_type = CategoricalDtype(categories=['<10%',"10-20%", "20-30%","30-40%",'40-50%', "50-75%",'75%+'],ordered=True)
df.success = df.success.astype(cat_type)

cat_type = CategoricalDtype(categories=['Never',"2", "3","4",'5', "6",'Very frequently'],ordered=True)
df.lof = df.lof.astype(cat_type)

# drop other genders

other_genders = len((df.loc[(df.gender != 'male') & (df.gender != 'female')]))
df = df.loc[(df.gender == 'male') | (df.gender == 'female')]

print(f'number of responses removed: {other_genders}')

# let score start at 0, replace NA by 0, and make dtype categorical

df['score'] = df.score - 1
df[['field','seniority','gender','ind','question']] = df[['field','seniority','gender','ind','question']].astype('category')
df = df.replace({7:0})

#complete case analyses: dropna

predrop = len(df)
df = df.dropna()
postdrop = len(df)
print(f'rows with nans removed: {predrop - postdrop}')
print(f'total responses: {postdrop}')


df.head()
```

```
number of responses removed: 336
rows with nans removed: 2306
total responses: 8622
```

Out[10]:

|  | field | seniority | gender | ind | success | lof | question | score |
| --- | --- | --- | --- | --- | --- | --- | --- | --- |
| 1 | Arts & Hum | 11-20 | female | 1 | 20-30% | 4 | R4 | 1.0 |
| 2 | Life & Biomed | 21-30 | female | 2 | 30-40% | 4 | R4 | 1.0 |
| 3 | Arts & Hum | 21-30 | female | 3 | 75%+ | Very frequently | R4 | 0.0 |
| 4 | Arts & Hum | 21-30 | male | 4 | 10-20% | 3 | R4 | 0.0 |
| 5 | Social Science | 11-20 | male | 5 | 30-40% | 2 | R4 | 1.0 |

In [11]:

```
# data from how many respondents

len(df.ind.unique())
```

Out[11]:

```
647
```

In [12]:

```
#  coordinates and data for pymc model
q_idx = df.question.cat.codes.values
s_idx = df.seniority.cat.codes.values
g_idx = df.gender.cat.codes.values
i_idx = df.ind.cat.codes.values
f_idx = df.field.cat.codes.values
l_idx = df.lof.cat.codes.values
suc_idx = df.success.cat.codes.values

q_codes = df.question.cat.categories.values
s_codes = df.seniority.cat.categories.values
g_codes = df.gender.cat.categories.values
i_codes = df.ind.cat.categories.values
f_codes = df.field.cat.categories.values
l_codes = df.lof.cat.categories.values
suc_codes = df.success.cat.categories.values
cutpoint_codes = np.array(['cutpoint_1','cutpoint_2','cutpoint_3','cutpoint_4','cutpoint_5','cutpoint_6'])


coords = {'q_n':q_codes, 's_n':s_codes,'g_n':g_codes,'i_n':i_codes,
          'f_n':f_codes, 'c_n':cutpoint_codes, 'l_n':l_codes, 'suc_n':suc_codes}
```

# Total lof effect¶

In [15]:

```
with pm.Model(coords=coords) as lof_check:

    # data

    G = pm.MutableData("G", g_idx)
    F = pm.MutableData("F", f_idx)
    S = pm.MutableData("S", s_idx)
    Q = pm.MutableData("Q", q_idx)
    SUC = pm.MutableData("SUC", suc_idx)
    L = pm.MutableData("L", l_idx)

    # fixed hyperpriors for field, seniority and participant
    
    s_field = pm.Uniform('s_field', 0,3)
    s_seniority = pm.Uniform('s_seniority',0,3)
    s_success = pm.Uniform('s_success',0,3)
    
    # prior for the cutpoints, one set of cutpoints per question
    
    cutpoints = pm.Normal('cutpoints',
                           mu=[0,1,2,3,4,5],
                           sigma=2,
                           transform=pm.distributions.transforms.univariate_ordered,
                           dims = ('q_n','c_n')
                           )

    # variable priors for the demographic predictors
    # non-centered to make sampling easier
    
    gender = pm.Normal("gender", 0.0, 1, dims = 'g_n')
    
    lof = pm.Normal('lof', 0,1,dims = 'l_n')
    
    z_field = pm.Normal("z_field", 0.0, 1, dims = 'f_n')
    field = pm.Deterministic("field", z_field * s_field, dims = 'f_n')

    z_success = pm.Normal("z_success", 0.0, 1, dims = 'suc_n')
    success = pm.Deterministic("success", z_success * s_success, dims = 'suc_n')
    
    z_seniority = pm.Normal("z_seniority", 0.0, 1.0, dims = 's_n')
    seniority = pm.Deterministic("seniority", z_seniority * s_seniority, dims = 's_n')

    phi = lof[L] + gender[G] + field[F] + seniority[S] + success[SUC] # 

    y = pm.OrderedLogistic("y", phi, cutpoints[Q], observed=df.score,compute_p = False)
    
    pr = pm.sample_prior_predictive()
```

```
Sampling: [cutpoints, gender, lof, s_field, s_seniority, s_success, y, z_field, z_seniority, z_success]
```

In [16]:

```
# plot the priors

variables = ['cutpoints','gender','s_field','s_seniority','field','seniority', 'success','lof']
fig, axs = plt.subplots(2,4,figsize = (15,7))

for ax, var in zip(axs.flat, variables):
    if len(pr.prior[var].shape) > 2:
        az.plot_posterior(pr.prior[var][:,:,0], ax=ax)
    else:
        az.plot_posterior(pr.prior[var][:,:], ax=ax)
```

In [17]:

```
# sample from the posterior

with lof_check:
    trace = pm.sample(5000,
                      tune = 1000,
                      return_inferencedata = True,
                      idata_kwargs={"log_likelihood": True},
                      random_seed = SEED,
                      target_accept = 0.99)
```

```
Auto-assigning NUTS sampler...
Initializing NUTS using jitter+adapt_diag...
Multiprocess sampling (4 chains in 4 jobs)
NUTS: [s_field, s_seniority, s_success, cutpoints, gender, lof, z_field, z_success, z_seniority]
```

100.00% [24000/24000 2:45:57<00:00 Sampling 4 chains, 0 divergences]

```
Sampling 4 chains for 1_000 tune and 5_000 draw iterations (4_000 + 20_000 draws total) took 9977 seconds.
```

In [18]:

```
# save the trace for later use

trace.to_netcdf('...')
```

Out[18]:

```
'C:\\Users\\u0124323\\OneDrive - KU Leuven\\Documents\\traces_ERF\\lof.nc'
```

In [20]:

```
az.summary(trace, var_names = ['gender','s_field','s_seniority','field','seniority', 'success','lof'])
```

Out[20]:

|  | mean | sd | hdi\_3% | hdi\_97% | mcse\_mean | mcse\_sd | ess\_bulk | ess\_tail | r\_hat |
| --- | --- | --- | --- | --- | --- | --- | --- | --- | --- |
| gender[female] | -0.259 | 0.402 | -1.014 | 0.492 | 0.007 | 0.005 | 3033.0 | 5558.0 | 1.0 |
| gender[male] | -0.324 | 0.402 | -1.073 | 0.428 | 0.007 | 0.005 | 3025.0 | 5554.0 | 1.0 |
| s\_field | 0.304 | 0.200 | 0.090 | 0.608 | 0.003 | 0.002 | 6501.0 | 8376.0 | 1.0 |
| s\_seniority | 0.116 | 0.096 | 0.000 | 0.259 | 0.001 | 0.001 | 5700.0 | 6662.0 | 1.0 |
| field[Arts & Hum] | -0.232 | 0.162 | -0.544 | 0.049 | 0.002 | 0.001 | 9089.0 | 9507.0 | 1.0 |
| field[Life & Biomed] | 0.277 | 0.156 | -0.006 | 0.571 | 0.002 | 0.001 | 8448.0 | 9195.0 | 1.0 |
| field[Natural Science] | -0.031 | 0.158 | -0.321 | 0.264 | 0.002 | 0.001 | 8704.0 | 10042.0 | 1.0 |
| field[Social Science] | 0.013 | 0.158 | -0.282 | 0.301 | 0.002 | 0.001 | 8718.0 | 9883.0 | 1.0 |
| field[Tech & Engineering] | -0.101 | 0.162 | -0.412 | 0.183 | 0.002 | 0.001 | 9097.0 | 10415.0 | 1.0 |
| seniority[0-10] | -0.078 | 0.090 | -0.249 | 0.068 | 0.001 | 0.001 | 14064.0 | 14989.0 | 1.0 |
| seniority[11-20] | 0.091 | 0.080 | -0.036 | 0.240 | 0.001 | 0.001 | 12395.0 | 14362.0 | 1.0 |
| seniority[21-30] | -0.024 | 0.075 | -0.154 | 0.116 | 0.001 | 0.001 | 15620.0 | 13000.0 | 1.0 |
| seniority[31-40] | 0.006 | 0.078 | -0.129 | 0.152 | 0.001 | 0.001 | 16456.0 | 14014.0 | 1.0 |
| seniority[>40] | -0.003 | 0.082 | -0.153 | 0.148 | 0.001 | 0.001 | 17789.0 | 14591.0 | 1.0 |
| success[<10%] | -0.311 | 0.155 | -0.607 | -0.025 | 0.002 | 0.001 | 8201.0 | 11140.0 | 1.0 |
| success[10-20%] | 0.092 | 0.140 | -0.168 | 0.358 | 0.002 | 0.001 | 7153.0 | 9709.0 | 1.0 |
| success[20-30%] | 0.189 | 0.139 | -0.067 | 0.455 | 0.002 | 0.001 | 7299.0 | 8425.0 | 1.0 |
| success[30-40%] | 0.142 | 0.140 | -0.115 | 0.406 | 0.002 | 0.001 | 7617.0 | 10316.0 | 1.0 |
| success[40-50%] | -0.001 | 0.141 | -0.272 | 0.260 | 0.002 | 0.001 | 7095.0 | 9546.0 | 1.0 |
| success[50-75%] | 0.206 | 0.140 | -0.066 | 0.465 | 0.002 | 0.001 | 7567.0 | 9249.0 | 1.0 |
| success[75%+] | -0.402 | 0.147 | -0.675 | -0.123 | 0.002 | 0.001 | 7649.0 | 10025.0 | 1.0 |
| lof[Never] | -0.305 | 0.339 | -0.965 | 0.321 | 0.006 | 0.004 | 3324.0 | 6018.0 | 1.0 |
| lof[2] | -0.191 | 0.341 | -0.825 | 0.471 | 0.006 | 0.004 | 3308.0 | 5742.0 | 1.0 |
| lof[3] | 0.140 | 0.342 | -0.494 | 0.797 | 0.006 | 0.004 | 3349.0 | 6006.0 | 1.0 |
| lof[4] | -0.137 | 0.341 | -0.773 | 0.516 | 0.006 | 0.004 | 3360.0 | 5987.0 | 1.0 |
| lof[5] | -0.040 | 0.340 | -0.701 | 0.586 | 0.006 | 0.004 | 3342.0 | 5921.0 | 1.0 |
| lof[6] | 0.209 | 0.342 | -0.450 | 0.842 | 0.006 | 0.004 | 3366.0 | 5939.0 | 1.0 |
| lof[Very frequently] | -0.274 | 0.341 | -0.919 | 0.371 | 0.006 | 0.004 | 3275.0 | 6586.0 | 1.0 |

## sampling stats¶

In [21]:

```
#R-hat (we used three chains) and ESS

az.summary(trace, var_names = ['gender','field','seniority', 'lof', 'success'])[['r_hat', 'ess_bulk']].T
```

Out[21]:

|  | gender[female] | gender[male] | field[Arts & Hum] | field[Life & Biomed] | field[Natural Science] | field[Social Science] | field[Tech & Engineering] | seniority[0-10] | seniority[11-20] | seniority[21-30] | ... | lof[5] | lof[6] | lof[Very frequently] | success[<10%] | success[10-20%] | success[20-30%] | success[30-40%] | success[40-50%] | success[50-75%] | success[75%+] |
| --- | --- | --- | --- | --- | --- | --- | --- | --- | --- | --- | --- | --- | --- | --- | --- | --- | --- | --- | --- | --- | --- |
| r\_hat | 1.0 | 1.0 | 1.0 | 1.0 | 1.0 | 1.0 | 1.0 | 1.0 | 1.0 | 1.0 | ... | 1.0 | 1.0 | 1.0 | 1.0 | 1.0 | 1.0 | 1.0 | 1.0 | 1.0 | 1.0 |
| ess\_bulk | 3033.0 | 3025.0 | 9089.0 | 8448.0 | 8704.0 | 8718.0 | 9097.0 | 14064.0 | 12395.0 | 15620.0 | ... | 3342.0 | 3366.0 | 3275.0 | 8201.0 | 7153.0 | 7299.0 | 7617.0 | 7095.0 | 7567.0 | 7649.0 |

2 rows × 26 columns

In [22]:

```
#see here: https://docs.pymc.io/en/v3/pymc-examples/examples/diagnostics_and_criticism/sampler-stats.html

#print number of divergences, ideally 0
print(f'divergences: {trace.sample_stats["diverging"].values.sum()}')

#print the acceptance rate
print(f'mean acceptance rate: {trace.sample_stats["acceptance_rate"].values.mean()}')

#compare the overall distribution of the energy levels with the change of energy between successive samples. Ideally, they should be very similar
az.plot_energy(trace, figsize=(6, 4));
```

```
divergences: 0
mean acceptance rate: 0.9867530670630226
```

## Results¶

In [25]:

```
# generate posterior predictive samples
# we generate them for the entire sample set to each of the categories of lof in turn
ppcs = {}
with lof_check:
    for i in range(len(l_codes)):
        pm.set_data({"L": np.repeat(i, len(df))})
        thinned_trace = trace.sel(draw=slice(None, None, 9))
        ppc = pm.sample_posterior_predictive(thinned_trace, progressbar = True)
        ppcs[i] = ppc
```

```
Sampling: [y]
```

100.00% [2224/2224 01:30<00:00]

```
Sampling: [y]
```

100.00% [2224/2224 01:30<00:00]

```
Sampling: [y]
```

100.00% [2224/2224 01:29<00:00]

```
Sampling: [y]
```

100.00% [2224/2224 01:31<00:00]

```
Sampling: [y]
```

100.00% [2224/2224 01:30<00:00]

```
Sampling: [y]
```

100.00% [2224/2224 01:29<00:00]

```
Sampling: [y]
```

100.00% [2224/2224 01:30<00:00]

In [26]:

```
#posterior predictive counts of the various qrp response options


lofdatas = [np.random.choice(np.ravel(ppcs[i].posterior_predictive['y'].values), 
                          size = 20000,
                         replace = True) for i in range(len(l_codes))]

ppcdf = pd.DataFrame(lofdatas, index = l_codes).T

sns.countplot(data = ppcdf.stack().reset_index().rename(columns = {0:'QRP', 'level_1':'LOF'}),
              x = 'QRP',
              hue = 'LOF')

plt.show()
```

In [32]:

```
# plot difference between coefficients for LOF

fig, axs = plt.subplot_mosaic("ABC;DEF;GGG",figsize = (15,13))

lofs = [trace.posterior['lof'][:,:,i] for i in range(7)]

no_lof = lofs[0]

for ax, (j,i) in zip(["A","B",'C','D','E','F'], enumerate(lofs[1:])):
    az.plot_posterior(i - no_lof, ax=axs[ax])
    axs[ax].set_title(f'Difference no lof and score {j+2}', fontsize = 15)
    
plt.suptitle('Differences between levels of lack of funding', fontsize = 25)

sns.countplot(data = ppcdf.stack().reset_index().rename(columns = {0:'QRP', 'level_1':'LOF'}),
              x = 'QRP',
              hue = 'LOF',
             ax = axs['G'])
axs['G'].set_title('Posterior predictive samples countplot', fontsize = 18)
axs['G'].set_xticklabels(['Never',2,3,4,5,6,'Almost always'])
plt.tight_layout()

plt.savefig('...', dpi = 300)

plt.show()
```
