# Supplementary material for: Questionable research practices in competitive grant funding: A survey
Source: PLoS One. 2023 Nov 2;18(11):e0293310. doi: 10.1371/journal.pone.0293310 (PMC10621923; doi:10.1371/journal.pone.0293310)
Supplement: S3 File — The code can also be accessed through the OSF page of the project (https://osf.io/jk6wd/). (ZIP) [file pone.0293310.s015.zip › S13 File/ERF_success_hypothesis5_upload.html]

ERF\_success\_hypothesis5\_upload


In [2]:

```
import pandas as pd
import numpy as np
import pickle

# for the stats
import pymc as pm
import bambi as bmb
from scipy.special import expit, logit


# for plotting
import seaborn as sns
import matplotlib.pyplot as plt
import arviz as az

# for DAGs

import collections.abc
#causalgraphicalmodels needs the four following aliases to be done manually.
collections.Iterable = collections.abc.Iterable
collections.Mapping = collections.abc.Mapping
collections.MutableSet = collections.abc.MutableSet
collections.MutableMapping = collections.abc.MutableMapping
from causalgraphicalmodels import CausalGraphicalModel
import daft
```

In [3]:

```
# versions

print('\n'.join(f'{m.__name__}=={m.__version__}' for m in globals().values() if getattr(m, '__version__', None)))
```

```
pandas==1.5.3
numpy==1.24.2
pymc==5.1.2
bambi==0.10.0
seaborn==0.12.2
arviz==0.15.1
daft==0.1.2
```

In [4]:

```
sns.set_palette("Dark2")
sns.set_style("darkgrid")
sns.set_context("paper", font_scale=1.5)
```

In [5]:

```
# seed

SEED = 2808

np.random.seed(SEED)
```

# Hypothesis¶

## Description¶

**SUCCESS HYPOTHESIS 5: The rate of HALF is higher for those wo report higher scores (scale 1 - 7) for success in grant funding applications.**

A participant has '1' for HALF if they responded '2' or more on at least half of the applicant or reviewer QRP questions. Success is measured on an ordinal scale: 1 (0% - 10%), 2 (11% - 20%), 3 (21% - 30%), 4 (31% - 40%), 5 (41% - 50%), 6 (51% - 75%), 7(76% - 100%).

Panelist qrps are left out of this analysis because this would force us to omit from the data any respondent that hasn't filled in the 'panelist' questions. Because early career researchers often have not been part of funding panels, this could create a bias and limit our sample size. Moreover, there is only one reviewer qrp in the questionnaire, and it is very similar to one of the reviewer qrps.

## Reporting¶

In the preregistered analysis code, we had written that we would report model 1 in the paper. The model reported here is very similar (same variables and data included), but adds hierarchical structure in the shape of hyperpriors for field and seniority. We do this because some of the gender x field x seniority cells are empty or have very few responses. The multi-level structure allows the model to inform field/seniority groups with a lot of data to inform those with little data. Adding the hierarchical structure did not affect the results substantially. The preregistered model is available on the OSF page of the project.

## Justification¶

We expect higher qrp levels for those with higher success rates on the basis of:

- the pilot study:
  - AL1: regression with continuous data shows a weak increase of AL1 as success rates increase. Transforming these data into our ordinal categories shows that this is mainly due to the highest category (+.75), and that there is no effect for the lower categories.
  - hmto: Similar to AL1\_app, the relation between success rate and hmto is strong for the highest category of success, but there is no clear linear relation between success rate and qrps.
- existing research: None, as far as we now.
- conceptual: many of the qrp's seem (purely conceptually) likely to improve the chances of those who use them, e.g. cite potential reviewers, do more research in advance, etc. Hence, we would expect success to increase as the rate of qrp's increase. This conceptual justification is the main justification for testing this hypothesis.
- field and career stage are included in the models because previous research suggests they play a role in the context of research integrity. We did not include 'continent' because we have no reason (neither pilot results nor previous research) to think it is relevant. In exploratory analysis, we will rerun the same models with additional variables such as continent.

# DAG¶

In [5]:

```
# draw the dag with our causal assumptions

dag_success = CausalGraphicalModel(nodes=["QRP", "G", "Age",'Field','Success'], 
                              edges=[("Age", "QRP"), ("Age", "QRP"), ("Field", "QRP"), 
                                     ('G','Age'),('G','QRP'),('G','Field'), ('Age','Field'),
                                     ('QRP','Success'), ('G','Success'), ('Field','Success'), ('Age','Success')
                                    ])
dag_success.draw()
```

Out[5]:

G

G


Field

Field


G->Field


Age

Age


G->Age


Success

Success


G->Success


QRP

QRP


G->QRP


Field->Success


Field->QRP


Age->Field


Age->Success


Age->QRP


QRP->Success

In [6]:

```
# function to check backdoor paths


def backdoor(dag, predictor, outcome):
    all_adjustment_sets = dag.get_all_backdoor_adjustment_sets(predictor, outcome)
    for s in all_adjustment_sets:
        if all(not t.issubset(s) for t in all_adjustment_sets if t != s):
            if s != {"U"}:
                print(s)

                
# For gender

backdoor(dag_success,'QRP','Success')
```

```
frozenset({'Age', 'G', 'Field'})
```

note: We are interested in the effect of QRPs on success. Hence, we will include seniority, gender and field as controls.

# Indices & Data¶

In [7]:

```
# data

df = pd.read_csv('...')

r_qrp = ['R4', 'R5', 'R6','R7','R8']
a_qrp = ['A2','A3','A5', 'A6','A8', 'A9', 'A10', 'A11','A14','A15']
demo = ['C1', 'C2', 'C3', 'C4']


# make a copy of the df

df = df[['C1','C3','C4','A20'] + a_qrp + r_qrp].copy()

# drop other genders and nans

other_genders = len((df.loc[(df.C4 != 'male') & (df.C4 != 'female')]))
df = df.loc[(df.C4 == 'male') | (df.C4 == 'female')]

predrop = len(df)
df = df.dropna()
postdrop = len(df)

print(f'number of non-male/female responses removed: {other_genders}')
print(f'rows with nans removed: {predrop - postdrop}')
print(f'total responses: {postdrop}')

# HALF indicator
half = []
for idx,row in df.iterrows(): #iterate over df get the scores
    count = 0
    for i in a_qrp + r_qrp:
        if row[i] > 1:
            count += 1
    if count > 7: 
        half.append(1)
    else:
        half.append(0)

df['HALF'] = half
# rename demo columns

df.rename(columns = {'C1':'field','C3':'seniority','C4':'gender', 'A20':'success'},inplace=True)

# set dtype to categorical

df[['field','seniority','gender','HALF']] = df[['field','seniority','gender','HALF']].astype('category')

# turn outcome into ordered categorical

from pandas.api.types import CategoricalDtype

cat_type = CategoricalDtype(categories=['<10%',"10-20%", "20-30%","30-40%",'40-50%', "50-75%",'75%+'],ordered=True)
df.success = df.success.astype(cat_type)

df.head()
```

```
number of non-male/female responses removed: 21
rows with nans removed: 190
total responses: 493
```

Out[7]:

|  | field | seniority | gender | success | A2 | A3 | A5 | A6 | A8 | A9 | A10 | A11 | A14 | A15 | R4 | R5 | R6 | R7 | R8 | HALF |
| --- | --- | --- | --- | --- | --- | --- | --- | --- | --- | --- | --- | --- | --- | --- | --- | --- | --- | --- | --- | --- |
| 1 | Arts & Hum | 11-20 | female | 20-30% | 7.0 | 1.0 | 7.0 | 1.0 | 1.0 | 2.0 | 1.0 | 5.0 | 1.0 | 4.0 | 2.0 | 2.0 | 2.0 | 2.0 | 1.0 | 1 |
| 2 | Life & Biomed | 21-30 | female | 30-40% | 1.0 | 6.0 | 3.0 | 1.0 | 5.0 | 3.0 | 5.0 | 2.0 | 1.0 | 1.0 | 2.0 | 1.0 | 2.0 | 1.0 | 1.0 | 1 |
| 4 | Arts & Hum | 21-30 | male | 10-20% | 7.0 | 1.0 | 3.0 | 1.0 | 1.0 | 7.0 | 1.0 | 3.0 | 1.0 | 3.0 | 1.0 | 1.0 | 1.0 | 2.0 | 1.0 | 0 |
| 5 | Social Science | 11-20 | male | 30-40% | 7.0 | 2.0 | 6.0 | 3.0 | 6.0 | 5.0 | 2.0 | 7.0 | 1.0 | 1.0 | 2.0 | 7.0 | 2.0 | 1.0 | 1.0 | 1 |
| 6 | Life & Biomed | 11-20 | female | 10-20% | 5.0 | 5.0 | 3.0 | 2.0 | 2.0 | 5.0 | 5.0 | 3.0 | 3.0 | 3.0 | 4.0 | 1.0 | 2.0 | 1.0 | 2.0 | 1 |

In [8]:

```
#  data for the pymc model

s_idx = df.seniority.cat.codes.values
g_idx = df.gender.cat.codes.values
half_idx = df.HALF.cat.codes.values
f_idx = df.field.cat.codes.values
success_idx = df.success.cat.codes.values


# coordinates
q_codes = df.HALF.cat.categories.values
s_codes = df.seniority.cat.categories.values
g_codes = df.gender.cat.categories.values
f_codes = df.field.cat.categories.values
cutpoint_codes = np.array(['cutpoint_1','cutpoint_2','cutpoint_3','cutpoint_4','cutpoint_5','cutpoint_6'])


# dct with the coordinates

coords = {'q_n':q_codes, 's_n':s_codes,'g_n':g_codes,
          'f_n':f_codes, 'c_n':cutpoint_codes}
```

# Model¶

In [9]:

```
with pm.Model(coords=coords) as success_hyp5:

    # data

    G = pm.MutableData("G", g_idx)
    F = pm.MutableData("F", f_idx)
    S = pm.MutableData("S", s_idx)
    Q = pm.MutableData("Q", half_idx)

    # fixed hyperpriors for field, seniority and participant
    
    s_field = pm.Uniform('s_field',0,3)
    s_seniority = pm.Uniform('s_seniority',0,3)
    
    # prior for the cutpoints, one set of cutpoints per question
    
    cutpoints = pm.Normal('cutpoints',
                           mu=[0,1,2,3,4,5],
                           sigma=2,
                           transform=pm.distributions.transforms.univariate_ordered,
                           dims = ('c_n')
                           )

    # variable priors for the demographic predictors
    # non-centered to make sampling easier
    
    gender = pm.Normal("gender", 0.0, 1, dims = 'g_n')
    
    qrp = pm.Normal("qrp", 0.0, 1, dims = 'q_n')
    
    z_field = pm.Normal("z_field", 0.0, 1, dims = 'f_n')
    field = pm.Deterministic("field", z_field * s_field, dims = 'f_n')
    
    z_seniority = pm.Normal("z_seniority", 0.0, 1.0, dims = 's_n')
    seniority = pm.Deterministic("seniority", z_seniority * s_seniority, dims = 's_n')
    


    phi = gender[G] +  qrp[Q] + field[F] + seniority[S] # 

    y = pm.OrderedLogistic("y", phi, cutpoints, observed=success_idx,compute_p = False)
    
    pr = pm.sample_prior_predictive()
```

```
Sampling: [cutpoints, gender, qrp, s_field, s_seniority, y, z_field, z_seniority]
```

In [10]:

```
# plot the priors

variables = ['gender','s_field','s_seniority','field','seniority','qrp','cutpoints']
fig, axs = plt.subplots(2,4,figsize = (15,7))

for ax, var in zip(axs.flat, variables):
    if len(pr.prior[var].shape) > 2:
        az.plot_posterior(pr.prior[var][:,:,0], ax=ax)
    else:
        az.plot_posterior(pr.prior[var][:,:], ax=ax)
```

In [ ]:

```
# sample from the posterior

with success_hyp5:
    trace = pm.sample(10000,
                      tune = 2000,
                      return_inferencedata = True,
                      random_seed = SEED,
                      target_accept = 0.97)
```

In [6]:

```
# save the trace for later use

# trace.to_netcdf('...')

# to load

trace = az.from_netcdf('...')
```

In [7]:

```
pd.set_option('display.max_rows', 500)
az.summary(trace)
```

Out[7]:

|  | mean | sd | hdi\_3% | hdi\_97% | mcse\_mean | mcse\_sd | ess\_bulk | ess\_tail | r\_hat |
| --- | --- | --- | --- | --- | --- | --- | --- | --- | --- |
| gender[female] | 0.735 | 0.616 | -0.451 | 1.857 | 0.004 | 0.003 | 20470.0 | 25637.0 | 1.0 |
| gender[male] | 0.782 | 0.614 | -0.394 | 1.913 | 0.004 | 0.003 | 20647.0 | 25151.0 | 1.0 |
| qrp[0] | 0.810 | 0.609 | -0.346 | 1.940 | 0.004 | 0.003 | 18624.0 | 24210.0 | 1.0 |
| qrp[1] | 0.686 | 0.610 | -0.451 | 1.833 | 0.004 | 0.003 | 19003.0 | 24502.0 | 1.0 |
| z\_field[Arts & Hum] | 1.289 | 0.687 | 0.059 | 2.618 | 0.004 | 0.003 | 27675.0 | 25844.0 | 1.0 |
| z\_field[Life & Biomed] | -0.382 | 0.637 | -1.560 | 0.808 | 0.005 | 0.003 | 17910.0 | 24340.0 | 1.0 |
| z\_field[Natural Science] | 0.112 | 0.616 | -1.068 | 1.257 | 0.004 | 0.003 | 25625.0 | 26032.0 | 1.0 |
| z\_field[Social Science] | 0.104 | 0.615 | -1.083 | 1.228 | 0.004 | 0.003 | 24482.0 | 26867.0 | 1.0 |
| z\_field[Tech & Engineering] | -0.320 | 0.678 | -1.586 | 0.940 | 0.004 | 0.003 | 25204.0 | 27206.0 | 1.0 |
| z\_seniority[0-10] | 0.359 | 0.622 | -0.797 | 1.553 | 0.004 | 0.003 | 28149.0 | 25908.0 | 1.0 |
| z\_seniority[11-20] | -0.768 | 0.698 | -2.091 | 0.495 | 0.006 | 0.004 | 15019.0 | 19753.0 | 1.0 |
| z\_seniority[21-30] | -0.219 | 0.578 | -1.306 | 0.857 | 0.004 | 0.003 | 18639.0 | 27321.0 | 1.0 |
| z\_seniority[31-40] | 0.492 | 0.564 | -0.588 | 1.549 | 0.003 | 0.003 | 26831.0 | 27087.0 | 1.0 |
| z\_seniority[>40] | 1.049 | 0.620 | -0.122 | 2.209 | 0.004 | 0.003 | 26170.0 | 25727.0 | 1.0 |
| s\_field | 0.557 | 0.416 | 0.001 | 1.288 | 0.004 | 0.003 | 9893.0 | 13863.0 | 1.0 |
| s\_seniority | 0.638 | 0.431 | 0.094 | 1.443 | 0.004 | 0.003 | 9842.0 | 12492.0 | 1.0 |
| cutpoints[cutpoint\_1] | -1.332 | 0.687 | -2.594 | -0.013 | 0.004 | 0.003 | 23825.0 | 26829.0 | 1.0 |
| cutpoints[cutpoint\_2] | 0.358 | 0.669 | -0.874 | 1.638 | 0.004 | 0.003 | 24525.0 | 27352.0 | 1.0 |
| cutpoints[cutpoint\_3] | 1.342 | 0.668 | 0.071 | 2.564 | 0.004 | 0.003 | 24360.0 | 26786.0 | 1.0 |
| cutpoints[cutpoint\_4] | 1.965 | 0.668 | 0.704 | 3.198 | 0.004 | 0.003 | 24185.0 | 27207.0 | 1.0 |
| cutpoints[cutpoint\_5] | 2.730 | 0.670 | 1.436 | 3.943 | 0.004 | 0.003 | 24138.0 | 26952.0 | 1.0 |
| cutpoints[cutpoint\_6] | 3.945 | 0.678 | 2.693 | 5.219 | 0.004 | 0.003 | 24373.0 | 28324.0 | 1.0 |
| field[Arts & Hum] | 0.640 | 0.453 | -0.061 | 1.449 | 0.004 | 0.003 | 13346.0 | 16884.0 | 1.0 |
| field[Life & Biomed] | -0.099 | 0.357 | -0.715 | 0.553 | 0.003 | 0.002 | 19574.0 | 15796.0 | 1.0 |
| field[Natural Science] | 0.103 | 0.376 | -0.528 | 0.810 | 0.003 | 0.002 | 19710.0 | 15852.0 | 1.0 |
| field[Social Science] | 0.100 | 0.373 | -0.542 | 0.784 | 0.003 | 0.002 | 19658.0 | 17247.0 | 1.0 |
| field[Tech & Engineering] | -0.096 | 0.376 | -0.815 | 0.554 | 0.003 | 0.002 | 23346.0 | 16593.0 | 1.0 |
| seniority[0-10] | 0.247 | 0.438 | -0.507 | 1.064 | 0.004 | 0.003 | 18732.0 | 15980.0 | 1.0 |
| seniority[11-20] | -0.317 | 0.390 | -1.018 | 0.397 | 0.003 | 0.002 | 18797.0 | 15662.0 | 1.0 |
| seniority[21-30] | -0.047 | 0.392 | -0.739 | 0.678 | 0.003 | 0.002 | 17569.0 | 14786.0 | 1.0 |
| seniority[31-40] | 0.306 | 0.413 | -0.393 | 1.075 | 0.003 | 0.002 | 17238.0 | 15733.0 | 1.0 |
| seniority[>40] | 0.600 | 0.452 | -0.140 | 1.428 | 0.004 | 0.003 | 15623.0 | 16455.0 | 1.0 |

## sampling stats¶

In [13]:

```
#R-hat (we used three chains) and ESS

az.summary(trace, var_names = ['gender','field','seniority','s_field', 's_seniority'])[['r_hat', 'ess_bulk']].T
```

Out[13]:

|  | gender[female] | gender[male] | field[Arts & Hum] | field[Life & Biomed] | field[Natural Science] | field[Social Science] | field[Tech & Engineering] | seniority[0-10] | seniority[11-20] | seniority[21-30] | seniority[31-40] | seniority[>40] | s\_field | s\_seniority |
| --- | --- | --- | --- | --- | --- | --- | --- | --- | --- | --- | --- | --- | --- | --- |
| r\_hat | 1.0 | 1.0 | 1.0 | 1.0 | 1.0 | 1.0 | 1.0 | 1.0 | 1.0 | 1.0 | 1.0 | 1.0 | 1.0 | 1.0 |
| ess\_bulk | 20470.0 | 20647.0 | 13346.0 | 19574.0 | 19710.0 | 19658.0 | 23346.0 | 18732.0 | 18797.0 | 17569.0 | 17238.0 | 15623.0 | 9893.0 | 9842.0 |

In [14]:

```
#see here: https://docs.pymc.io/en/v3/pymc-examples/examples/diagnostics_and_criticism/sampler-stats.html

#print number of divergences, ideally 0
print(f'divergences: {trace.sample_stats["diverging"].values.sum()}')

#print the acceptance rate
print(f'mean acceptance rate: {trace.sample_stats["acceptance_rate"].values.mean()}')

#compare the overall distribution of the energy levels with the change of energy between successive samples. Ideally, they should be very similar
az.plot_energy(trace, figsize=(6, 4));
```

```
divergences: 0
mean acceptance rate: 0.9664439650786583
```

## Results¶

In [16]:

```
# plot difference between coefficients female and male

fig, ax = plt.subplots(figsize = (5,2))

no_half = trace.posterior['qrp'][:,:,0]
half = trace.posterior['qrp'][:,:,1]

az.plot_posterior(half - no_half, round_to = 3 ,ax=ax)

plt.show()
```

In [17]:

```
# for table 3 in the paper: get the description of the difference

az.summary(half - no_half)
```

Out[17]:

|  | mean | sd | hdi\_3% | hdi\_97% | mcse\_mean | mcse\_sd | ess\_bulk | ess\_tail | r\_hat |
| --- | --- | --- | --- | --- | --- | --- | --- | --- | --- |
| qrp | -0.124 | 0.164 | -0.432 | 0.183 | 0.001 | 0.001 | 41670.0 | 27993.0 | 1.0 |

In [18]:

```
# generate posterior predictive samples
# we generate them for the entire sample set to HALF == 1 (other characteristics intact) and HALF == 0 (idem)

ppcs = {}
with success_hyp5:
    for i in range(2):
        pm.set_data({"Q": np.repeat(i, len(df))})
        ppc = pm.sample_posterior_predictive(trace, progressbar = False)
        ppcs[i] = ppc
```

```
Sampling: [y]
Sampling: [y]
```

In [20]:

```
#posterior predictive counts of the various success categories for qrp vs no_qrp

no_qrp = np.random.choice(np.ravel(ppcs[0].posterior_predictive['y'].values), size = 20000, replace=True)
qrp = np.random.choice(np.ravel(ppcs[1].posterior_predictive['y'].values), size = 20000, replace=True)

ppcdf = pd.DataFrame([qrp, no_qrp], index = ['qrp', 'no_qrp']).T
data = ppcdf.stack().reset_index().rename(columns = {0:'success', 'level_1':'QRPs'})
# save data to make figures for paper in other notebook

with open('...', 'wb') as handle:
    pickle.dump(data, handle, protocol=pickle.HIGHEST_PROTOCOL)

sns.countplot(data = data,
              x = 'success',
              hue = 'QRPs')

plt.show()
```

In [ ]:

```

```
